# Machine-intelligent multimodal algebot for intracavitary chemotherapy

**DOI:** 10.1038/s41565-026-02195-0

**Published:** 2026-06-22

**Authors:** Lin Lin, Haohui Li, Qi Zhou, Qi Ding, Xiang Sun, Jing Hu, Zhangli Zhao, Yaning Qin, Tiancheng Jiang, Honghao Ye, Xiaozhen Wu, Honghan Liu, Lu Huang, Chenghao Huang, Yahui Gao, Jinyao Tang, Xing Ma, Xiaohui Yan

**Affiliations:** 1 State Key Laboratory of Vaccines for Infectious Diseases & Fujian Engineering Research Center of Molecular Theranostic Technology, Xiang An Biomedicine Laboratory, School of Public Health, Xiamen University, Xiamen, China; 2 Institute for Neuroscience and Cardiovascular Research & The ZJE Institute, Edinburgh Medical School, The University of Edinburgh, Edinburgh, UK; 3 Sauvage Laboratory for Smart Materials & School of Integrated Circuits, Harbin Institute of Technology (Shenzhen), Shenzhen, China; 4 National Institute of Diagnostics and Vaccine Development in Infectious Diseases, Department of Laboratory Medicine, School of Public Health, Xiamen University, Xiamen, China; 5 Key Laboratory of Ministry of Education for Coastal and Wetland Ecosystems & School of Life Sciences, Xiamen University, Xiamen, China; 6 Department of Chemistry, The University of Hong Kong, Hong Kong, China; 7 State Key Laboratory of Synthetic Chemistry, The University of Hong Kong, Hong Kong, China

**Keywords:** Drug delivery, Biomedical engineering, Drug delivery

## Abstract

Intracavitary drug instillation is a crucial therapeutic strategy for treating bladder cancer. However, current methods are limited in efficacy due to insufficient tumour targeting and drug penetration across tissue barriers in pathophysiological conditions. Here we devise biohybrid magnetic algae microrobots with hierarchical nanoporous structure and develop an ‘algebot’-mediated, non-contact convective transport strategy to synergistically integrate targeted carrier transport, selective drug release and ultrafast tissue penetration. Our approach leverages machine-intelligent image feedback for autonomous navigation, magnetite-endowed multimodal control for reconfigurable swarming and flow-tuned convective diffusion for on-demand therapeutic delivery. We exemplify this approach with doxorubicin-loaded magnetic *Coscinodiscus granii* evaluated in a murine model of bladder tumour, demonstrating an over tenfold increase in drug permeation and substantially reduced tumour burden to less than 3% compared with conventional intravesical instillation in a preclinical trial of 1-week therapy without inducing systemic toxicity. Our drug delivery system offers a non-invasive solution to overcome complex biological barriers, advancing the efficacy and safety of intracavitary chemotherapy.

## Abstract (web summary)

A deep learning-guided image-feedback system enables non-invasive real-time navigation and spatiotemporally controlled intravesical drug release from magnetic biohybrid microrobots in a murine bladder tumour model, enhancing tissue penetration and therapeutic efficacy.

## Main

Intracavitary malignancies present a compelling therapeutic opportunity for which locally deployable interventions can offer a broadly translatable paradigm for precision treatment across anatomically accessible cancers. Bladder cancer is among the most prevalent types featuring high incidence, rapid progression and poor prognosis, and non-muscle-invasive bladder cancer accounts for approximately 75% of cases^1–6^. The standard treatment consists of transurethral resection of the bladder tumour followed by the intravesical instillation of therapeutic agents to eradicate residual tumour foci and reduce recurrence^7,8^. Although intravesical administration enables localized drug exposure with minimal systemic toxicity^9^, its efficacy is limited by poor penetration across the urothelial mucus layer and tumour extracellular matrix^10,11^, low lesion selectivity^12,13^ and rapid drug loss during bladder voiding^14,15^. These factors restrict drug retention and accumulation at the tumour site, thereby limiting the therapeutic efficacy of intravesical delivery.

Current efforts to improve intravesical delivery have primarily followed two approaches: external field-assisted instillation and carrier-mediated instillation. External field-based methods, including electromotive drug administration, radio-frequency-induced thermochemotherapy and low-energy shock-wave-enhanced chemotherapy, can enhance drug transport across bladder barriers, but require specialized instrumentation and offer limited control over lesion-specific delivery^16,17^. On the other hand, carrier-based systems, including surface-engineered nanocarriers^18–21^, magnetic composites^22,23^ and in situ forming hydrogels^24,25^, can prolong bladder residence and increase local drug exposure, yet remain constrained by factors such as inadequate tissue penetration, limited targeting capacity, instability in the bladder environment and adverse effects associated with prolonged carrier retention^10,26^. Therefore, the above strategies have yet to sufficiently integrate active targeting, efficient barrier penetration and controllable local release.

**Fig. 1:**
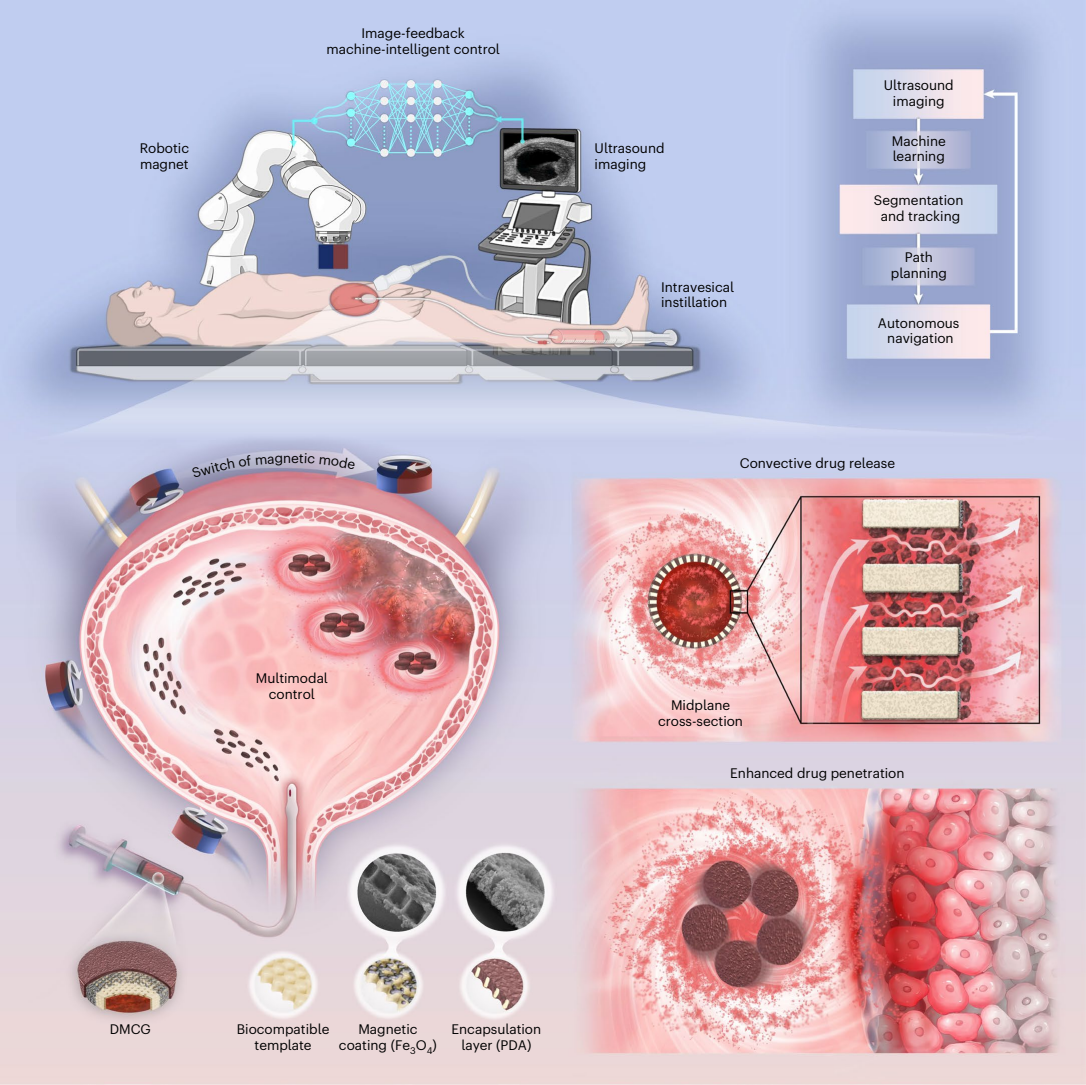
Proof of concept for targeted delivery, selective release and ultrafast penetration of chemotherapeutic drugs for intracavitary cancer therapy with drug-loaded magnetic *C. granii* microrobots. Top: workflow for applying machine-intelligent multimodal microrobots with active navigation and targeted drug delivery towards efficient intracavitary chemotherapy. Bottom left: schematic of the chemical composition, magnetic control, in vivo navigation and localized therapy of DMCG. Bottom right: convective release of drug molecules encapsulated within the hierarchical micro-nanostructured shell of DMCG and their enhanced penetration into malignant tumours. Top illustration created in BioRender; Lin, L. https://BioRender.com/kfr9u4q (2026).

Recent advances in nanotechnology and autonomous systems offer a sophisticated toolkit for overcoming the formidable biological barriers associated with bladder cancer^27–29^. Micro- and nanorobotic systems, in particular, provide a promising framework for intracavitary chemotherapy by enabling active, directed motion within confined luminal environments. However, the translation of these systems remains hindered by a lack of autonomous tumour targeting or reliance on direct mechanical interaction with the urothelium, which can compromise safety and operational robustness. Here we report a drug-loaded microrobot featuring a nanoporous architecture that exploits fluidically induced convection^30–33^ as a non-contact mechanism to facilitate deep tissue penetration and local transport. To achieve high-fidelity targeting, the platform is integrated with a deep learning-based imaging-feedback control system for real-time navigation and spatiotemporally controlled drug release (Fig. 1 and Supplementary Video 1). Using the microalga *Coscinodiscus granii* as a structural template, we leverage its naturally evolved hierarchical porosity for high-capacity drug loading and scalable synthesis. This autonomous microrobotic strategy establishes a versatile platform for targeted intravesical delivery, offering a level of control over transport and release kinetics that is currently unattainable with existing carrier systems.

## Fabrication of drug-loaded magnetic *C. granii*

**Extended Data Fig. 1:**
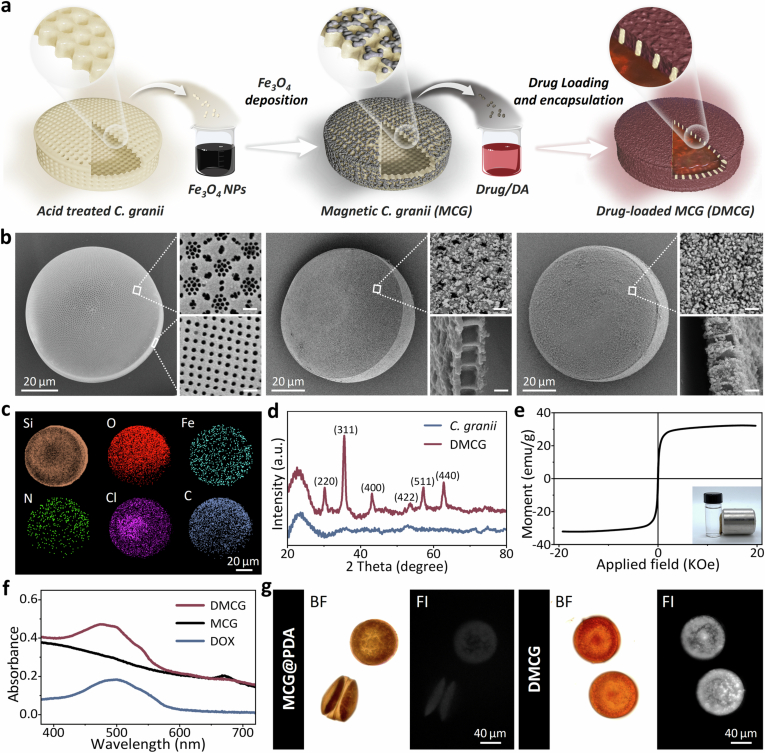
Fabrication and characterization of drug-loaded magnetic *C. granii* (DMCG) microrobots. **a**, Schematic illustration of the fabrication process of DMCG microrobots. First, *C. granii* undergo acid treatment to eliminate its intracellular matrix and form a SiO_2_ shell with porous-hollow morphology. Subsequently, Fe_3_O_4_ nanoparticles of approximately 10–30 nm in diameter are electrostatically adsorbed onto the surface of acid-treated *C. granii* for 12 h (tunable for higher magnetization) to yield magnetized algae, ie., magnetic *C. granii* (MCG). Finally, MCGs are co-incubated with a drug solution for internal loading, followed by addition of polydopamine (PDA) to form an encapsulation layer and seal off the nanopores on the MCG surface for secure drug packaging. **b**, Scanning electron microscopy (SEM) images of *C. granii*, MCG and DMCG. The insets (scale bar 500 nm) show enlarged views of the open (*C. granii*), partially closed (MCG) or fully closed (DMCG) nanopores on the valve face and/or girdling band. Cross-sectional views of the vacant (MCG) and filled (DMCG) microchambers are also shown. **c**, Element mapping of DMCG. **d**, X-ray diffraction (XRD) spectrum of DMCG. **e**, Magnetic hysteresis loop of DMCG. **f**, Ultraviolet-visible absorbance spectra of DOX, MCG and DMCG. **g**, Bright-field and fluorescence images of MCG and DOX-loaded DMCG. Representative images from three independently prepared samples with similar results. Source data

To engineer microrobots for intravesical delivery, we prioritized propulsion, drug loading and biocompatibility^34,35^. Building on prior biohybrid microrobot designs^35–37^, we selected the diatom species *C. granii* as a natural template as its hierarchical porous silica shell is ideal for drug encapsulation and controlled release. As in Extended Data Fig. 1a, drug-loaded magnetic *C. granii* (DMCG) microrobots were fabricated via sequential frustule hollowing, magnetic functionalization (MCG as the intermediate product) and drug encapsulation. This facile process fabricates a porous-hollow microrobot architecture with surface-bound magnetite nanoparticles for magnetic control and a polydopamine (PDA) sealing layer for drug packaging (Extended Data Fig. 1b and Supplementary Fig. 1).

Our structural and compositional analyses confirmed the successful assembly of DMCG as intended. Microscopy showed the preservation of the porous shell architecture after processing, uniform surface decoration by Fe_3_O_4_ nanoparticles and pore sealing after PDA coating (Extended Data Fig. 1b and Supplementary Fig. 1). Elemental mapping and diffraction analysis further verified the integration of the silica scaffold, magnetic component and polymer coating (Extended Data Fig. 1c,d). DMCG exhibited superparamagnetic behaviour with a saturation magnetization of 32.3 emu g^−1^, desirable for magnetic actuation (Extended Data Fig. 1e). Using doxorubicin (DOX) as a model cargo, we confirmed the capacity for drug loading by spectroscopy and fluorescence imaging, with a maximum loading efficiency of 27.95% (Extended Data Fig. 1f,g and Supplementary Fig. 2). These characterization data evidence DMCG as a magnetically responsive, drug-loadable microrobotic platform.

## Magnetic actuation and hydrodynamic control

**Fig. 2:**
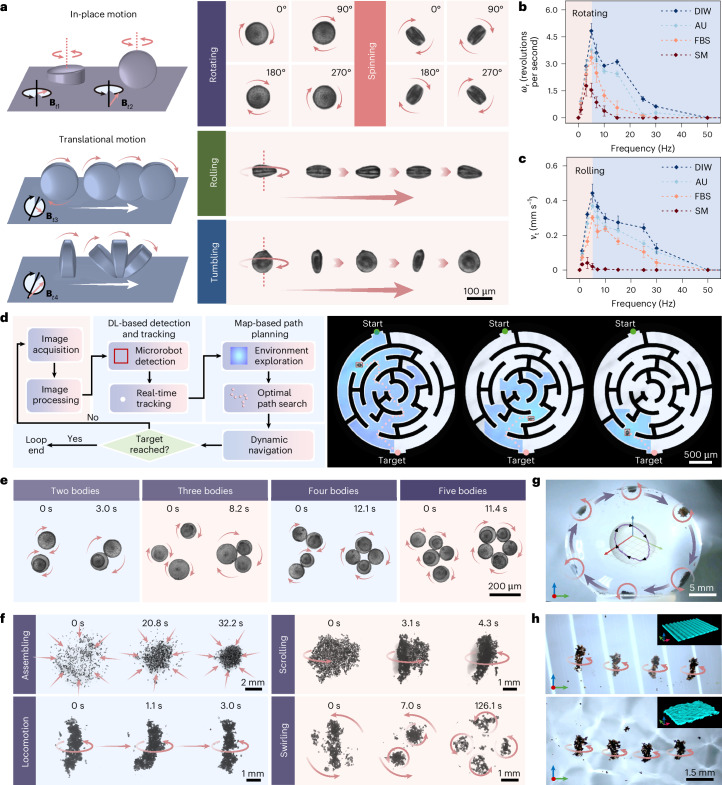
Motion regime, navigation strategy, multibody dynamics and reconfigurable swarming of DMCGs. **a**, Schematic and time sequence of the rotating, spinning, rolling and tumbling motion regimes. **b**,**c**, Angular and translational velocities of DMCGs in varying biofluids versus the magnetic field rotation frequency in the rotating (**b**) and rolling (**c**) regimes. Data are presented as mean ± s.d. (*n* = 5 independent samples per group). DIW, deionized water; AU, artificial urine; FBS, fetal bovine serum; SM, simulated mucus. **d**, Left: workflow for active exploration and autonomous navigation of DMCGs based on real-time image feedback, map-based path planning and closed-loop magnetic control. Right: time sequence of a DMCG’s automated navigation in a complex maze based on a breadth-first search algorithm. **e**, Rotation-induced multibody assembly of DMCGs, shown here the formation of two-, three-, four- and five-body configurations. **f**, Swarm control and reconfigurable patterns of large numbers of DMCGs for on-demand operations. DMCGs dispersed in a suspension were promptly assembled into a compact swarm, followed by scrolling and rolling for fast translation; on arrival at the target, the swarm was twisted to disassemble into smaller swirling clusters through a switch of the magnetic control mode. **g**, Gravity-resisting locomotion of a DMCG swarm inside a 3D hollow quartz sphere. **h**, Robust locomotion of DMCG swarms on a grooved surface (top) and a rough terrain (bottom), respectively. Source data

A tailored magnetic actuation strategy is required to drive the wheel-like DMCG, for which rotating magnetic fields (RMFs) provide precise control and robust responsiveness^38,39^. We established a programmable RMF framework that enabled switching between rotational and translational motion regimes, including in-place rotating/spinning with local revolution and net-displacement rolling/tumbling with translational movement (Fig. 2a and Supplementary Figs. 3 and 4). These motion regimes were also reproduced on ex vivo bladder tissue (Supplementary Video 2). DMCG’s motility was quantified in deionized water and representative biological media, including artificial urine, fetal bovine serum and simulated mucus. In all tested media, the angular (up to 4.8 revolutions per second in rotating) and translational (up to 0.45 mm s^−1^ in rolling) velocities of DMCG increased with the RMF frequency up to a step-out threshold, beyond which motion became asynchronous and declined^40^ (Fig. 2b,c and Supplementary Fig. 5); the step-out occurred at approximately 5 Hz in deionized water, artificial urine and fetal bovine serum, but shifted to approximately 3 Hz in simulated mucus. The overall DMCG motility decreased with increasing medium viscosity, with planar rotating/rolling remaining more robust than spinning/tumbling at higher frequencies beyond 30 Hz.

RMF programming further enabled directional steering and path editing by mapping time-varying sequences, via which DMCGs tracked predefined trajectories on both Petri dish and biological substrates (Supplementary Fig. 6 and Supplementary Video 3). We then integrated real-time imaging with deep learning-based feedback control for autonomous navigation, enabling path planning in complex maze environments with improved positional precision relative to manual control, reaching approximately 5 μm compared with approximately 20 μm for path editing (Fig. 2d, Supplementary Figs. 7 and 8 and Supplementary Video 4). Given that practical intravesical delivery probably requires multiple DMCGs, we also examined the collective dynamics, which are commonly encountered in drug delivery systems^41,42^. Under magnetic actuation, DMCGs formed synchronized and reconfigurable assemblies arising from coupled magnetic and hydrodynamic interactions^43^, without substantial loss in angular velocities (Fig. 2e and Supplementary Video 5). To extend control to larger workspaces, we implemented a nested rod–shell magnet setup (nRSM; Supplementary Fig. 9) that not only reproduced the principal motion modes of the coil-based platform (Supplementary Figs. 10 and 11) but also enabled reversible swarm compaction, transport and redistribution in a controllable manner via adjusting its distance and rotation frequency (Fig. 2f, Supplementary Figs. 12–18 and Supplementary Video 6). The swarms also remained operable under gravity-resisting and complex-surface conditions (Fig. 2g,h, Supplementary Figs. 19 and 20 and Supplementary Video 6), indicating excellent adaptability across harsh environments encountered in vivo, for example, rugae-like folds on the bladder wall.

**Fig. 3:**
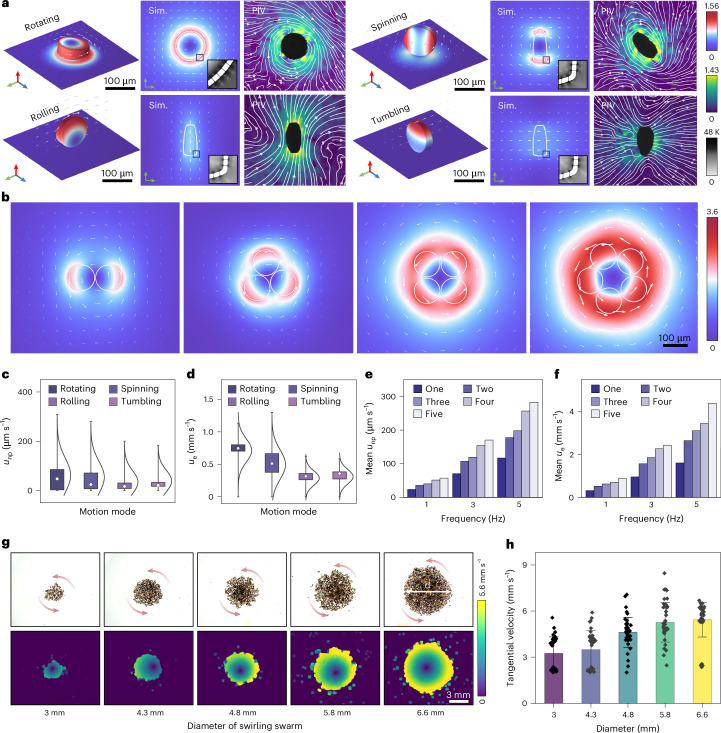
Flow disturbance and secondary fluidic effects arising from the motion of DMCGs. **a**, Numerical simulation (Sim.) and PIV results of the velocity field (mm s^−1^) in distinct motion regimes. Insets: flow shear rate (s^−1^) around nanopores in the DMCG shell. **b**, Simulation results of the velocity field (mm s^−1^) under multibody rotation modes. **c**,**d**, Flow velocities within the nanopores ${{{u}}}_{{\mathrm{np}}}\,$ (**c**) and in the external region ${{{u}}}_{{\mathrm{e}}}$ (**d**) of a moving DMCG under different motion regimes. Velocities were read from all finite-element mesh nodes in the indicated regions. For **c**, *n* = 4,693, 4,682, 4,684 and 4,686 nodes; for **d**, *n* = 10,678, 10,675, 10,664 and 10,666 nodes. Boxes span the 25th to 75th percentiles, white dots indicate the median, and whiskers extend to the minimum and maximum values. **e**,**f**, Mean flow velocities within the nanopores (**e**) and around the external region (**f**) of a single DMCG or DMCG multibody assembly in rotating regime versus varying rotation frequency and number of assembling units. **g**, Size-dependent tangential velocity of swirling DMCG swarms: (top) microscope images; (bottom) optical flow-based velocity contour measured at a rotation frequency of 3 Hz. **h**, Quantification of tangential velocity versus swarm size for the swirling swarms in **g**. Velocities were extracted from the corresponding experiment videos by optical flow analysis. Colour bar indicates tangential velocity (mm s^−^^1^). Each point represents one video frame. Bars indicate the mean and error bars indicate the s.d. (*n* = 40 video frames per group). Source data

To define how these motion regimes could be harnessed to facilitate drug delivery, we quantified the fluid disturbances generated by DMCGs using particle image velocimetry (PIV) and computational fluid dynamics (Supplementary Tables 1 and 2)^44,45^. The computational fluid dynamics simulations agreed well with the PIV experiments in both flow patterns and velocity magnitudes (Fig. 3a and Supplementary Fig. 21). The different motion regimes produced distinct flow signatures: rotating and spinning generated localized vortical flows around the DMCGs, whereas rolling and tumbling produced propulsive streams propagating along the DMCG translation direction (Fig. 3a). Comparatively, rotating generated the strongest near-surface shear around the nanoporous shell, whereas the other modes produced weaker pore-adjacent shear even in regions of elevated local velocity (Fig. 3a and Supplementary Fig. 22). The rotational flow was further amplified in multibody assemblies, with peak velocities up to multiple times higher than for a single DMCG (Fig. 3b and Supplementary Video 7). Quantitative analysis of the pore-scale and external flow confirmed that rotating perturbed both surrounding fluid and nanopore flow more strongly than spinning, tumbling or rolling (Fig. 3c,d). Further parametric simulations showed that the flow intensity increased with rotation frequency and assembly size (Fig. 3e,f). Consistent with this trend, the tangential velocity increased progressively from a single DMCG to multibody assemblies and larger swirling swarms with up to subcentimetres per second (Fig. 3g,h). These results identify distinct hydrodynamic modes for multimodal control suited to on-demand transport and release operations.

## Convective drug release and barrier penetration

**Fig. 4:**
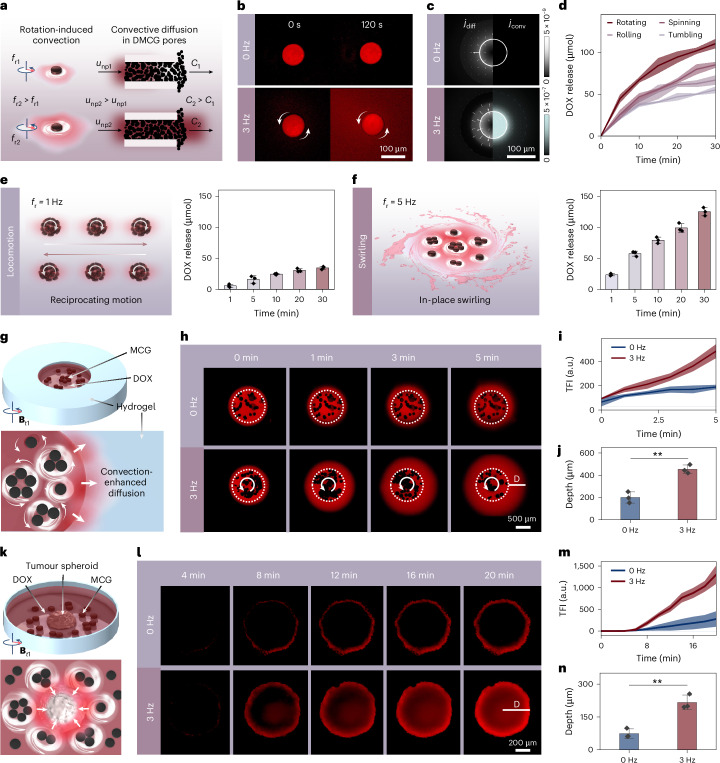
Convection-enhanced drug release and barrier penetration of DMCGs. **a**, Schematic of the convective diffusion mechanism for drug release from the nanoporous DMCGs. **b**, Time-lapse fluorescence imaging of drug release by a DOX-loaded DMCG in the rotating regime (3 Hz) versus static control (0 Hz). **c**, Simulation of convective and diffusive fluxes (*j*, nmol m^−2^ s^−1^) from a DMCG in the rotating regime (3 Hz) versus static control (0 Hz) for 30 s. **d**, Drug release over 30 min in a DMCG solution in different DMCG motion regimes (rotating, spinning, rolling and tumbling) at an RMF of frequency 5 Hz. **e**,**f**, Schematic (left) and cumulative DOX release over 30 min (right) from DMCG swarms under scrolling locomotion mode at 1 Hz (**e**) and in-place swirling mode at 5 Hz (**f**). Data in **e** and **f** represent mean ± s.d. from *n* = 3 independent experiments; error bars indicate s.d. **g**, Schematic of convection-enhanced drug penetration into a hydrogel matrix by rotating MCGs in a free-DOX solution. **h**–**j**, Time-lapse fluorescence imaging of DOX penetration into the hydrogel wall with rotating (3 Hz) versus stationary MCGs (**h**), with total fluorescence intensity (TFI) measured above the entire microwell (**i**) and depth of penetration (DOP) perpendicular to the hydrogel interface (**j**). Data in **i** and **j** represent mean ± s.d. from *n* = 3 independent hydrogel matrices; error bars/bands indicate s.d. **k**, Schematic of convection-enhanced drug penetration into a tumour spheroid by rotating MCGs in a free-DOX solution. **l**–**n**, Time-lapse fluorescence imaging of DOX penetration into the tumour spheroid with rotating (3 Hz) versus stationary MCGs (**l**), with TFI measured above the spheroid (**m**) and DOP into the tumour (**n**). Data in **m** and **n** represent mean ± s.d. from *n* = 3 independent tumour spheroids; error bars/bands indicate s.d. *P* values in **j** and **n** were determined by two-tailed Welch’s *t*-test (**j**, *P* = 0.0029; **n**, *P* = 0.0058). Statistical significance is indicated by *P ≤ 0.05, **P ≤ 0.01, ***P ≤ 0.001. Source data

On the basis of the advection–diffusion equation that governs the solute transport in an incompressible fluid, the release of an encapsulated drug (for example, DOX) from the DMCG core into the external environment with concentration ${C}_\mathrm{d}$ can be described by ${\partial C}_\mathrm{d}/\partial t={D}_\mathrm{d}{\nabla }^{2}{C}_\mathrm{d}-{\bf{u}}\cdot \nabla {C}_\mathrm{d}$. The speed of drug release is, therefore, co-determined by the diffusive flux and the convective flux across the nanopores on the DMCG shell. Although the former relies on the intrinsic diffusion coefficient of DOX molecules ${D}_\mathrm{d}$, the latter is determined by local fluid velocities due to flow perturbation by DMCG motion. Given that the nanopore velocities **u**_np_ are positively correlated with the rotation frequency *f* (Fig. 3e), we hypothesized that by increasing *f*, the drug release through the nanopores can be accelerated (Fig. 4a).

**Extended Data Fig. 2:**
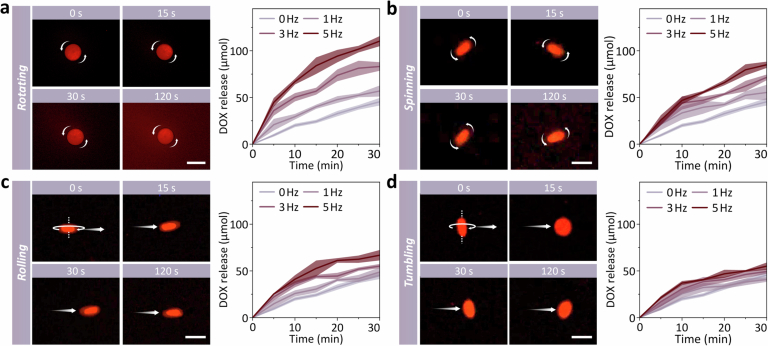
Comparative drug release from DMCGs under different motion regimes. **a-d**, Single DMCGs in rotating (**a**) spinning (**b**) rolling (**c**), and tumbling (**d**), motion regimes under a rotating magnetic field of varying operational frequencies. Left, time-lapse (0–120 s) fluorescence images of DOX release from a single DMCG at 3 Hz. White arrows indicate the movement direction of DMCG. Right, cumulative molar amount of DOX released over 30 min in a 5 mL suspension of dispersed DMCGs (1 mg mL^−1^) under the corresponding motion regimes at 0, 1, 3, 5 Hz. Data in **a-d** present mean ± s.d. from n = 3 independent experiments per group; error bands indicate s.d. Scale bar: 100 μm. The experiments show that rolling/tumbling exhibits much lower release compared with rotating/spinning at identical field frequencies. At 5 Hz, the release of DOX from DMCGs after 30 min in the rotating regime (110.32 μmol) is 129.8%, 165.2%, and 200.4% of that in spinning (84.99 μmol), rolling (66.78 μmol), and tumbling (55.05 μmol) regimes, respectively. Source data

We next examined whether the DMCG-induced flows could practically accelerate drug release. Because the rotating mode produced the strongest pore-adjacent flow and shear (Fig. 3a,c), we expected that it would enhance transport across the nanoporous shell. Indeed, fluorescence imaging and bulk assays showed that the rotating DMCG released more DOX than stationary controls, with release increasing by 25.3%, 83.2% and 143.1% at 1, 3 and 5 Hz, respectively (Fig. 4a,b and Extended Data Fig. 2a). To elaborate the origin of this enhancement, we extended the simulation model to consider convection-driven solute transport through the porous shell. The simulations showed that increasing the rotation frequency accelerated drug depletion from the DMCG core and increased outward convective fluxes across the shell (Fig. 4c, Supplementary Figs. 23 and 24 and Supplementary Video 8). Under the tested conditions, convective transport dominated over diffusion with one to two orders higher magnitude and scaled positively with actuation frequency. Multibody assemblies further enhanced release, although the kinetics became more complex with the assembly size (Supplementary Fig. 24c,d).

The drug release behaviour across motion regimes was also compared. At matched field frequencies, the rotating and spinning modes produced substantially higher DOX release than the rolling and tumbling modes (Fig. 4d and Extended Data Fig. 2b–d), consistent with their stronger local flow fields (Fig. 3a,c). At the swarm level, this regime dependence enabled functional switching between locomotion mode for transport and swirling mode for local release, under the combined effects of altered body size, specific surface area and drug concentration gradient. Accordingly, in-place swirling produced much higher release than reciprocating locomotion over the same interval, with total release over 30 min reaching 362.4% of that in the locomotion mode (Fig. 4e,f). These results show that DMCG release can be regulated by both actuation mode and collective state.

We then investigated whether DMCG-induced convection could improve transport across physical model barriers relevant to intravesical delivery in bladder cancer, where permeability is restricted by the mucus-like luminal interface and the urothelial barrier with tight junctions^46–48^. To address this with in vitro assays, we used two simplified barrier-mimicking systems: an agarose hydrogel matrix to represent the hydrated diffusion boundary barrier (Fig. 4g) and a tumour spheroid matrix to represent the cellular environment under cancerous conditions (Fig. 4k).

In the hydrogel assay, actuated magnetic *C. granii* (MCG) in free-DOX solution notably enhanced DOX penetration relative to the non-actuated control, showing a 155% increase in total fluorescence and approximately 300 μm larger penetration depth into the hydrogel after 5 min (Fig. 4h–j and Supplementary Figs. 25 and 26). Computational fluid dynamics simulations reproduced this frequency-dependent improvement and showed increased convective transport at the matrix interface, particularly for multibody assemblies (Supplementary Figs. 27 and 28 and Supplementary Video 9). For the tumour spheroid assay, rotating MCG increased DOX transport towards the spheroid interior, producing a 368.6% increase in fluorescence intensity and approximately 150 μm larger tissue penetration depth over 20 min than non-actuated controls (Fig. 4l–m and Supplementary Figs. 29 and 30). Further experiments showed that DMCGs remained largely non-adhesive to the tumour surface and preserved swarm integrity near solid boundaries (Supplementary Figs. 31–33). These results indicate that DMCG-induced convection can indeed enhance transport across model barriers and into tumour-like tissue in a robust manner.

## In vitro machine-intelligent intravesical delivery

**Fig. 5:**
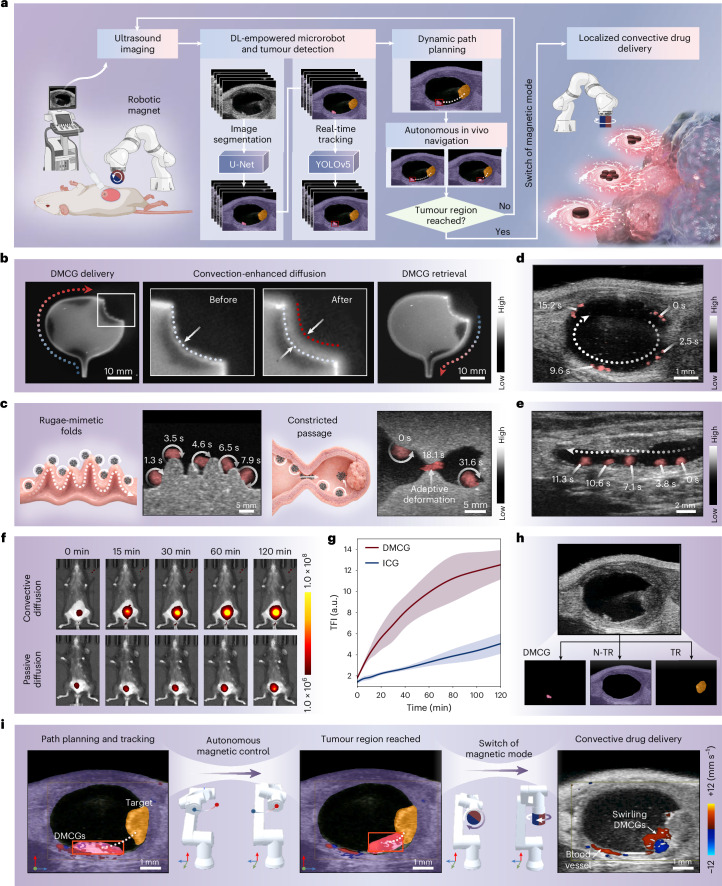
Validation of DMCG intracavitary drug delivery strategy with in vitro and in vivo bladder tumour models. **a**, Strategy of intravesical therapy with DMCGs empowered by deep learning (DL)-based closed-loop control. **b**, In vitro test in a hydrogel bladder tumour model without (before) and with (after) convection-facilitated drug penetration at the target site. Colour bar indicates fluorescence. **c**, In vitro validation of DMCG swarm navigation in phantom bladders with rugae-mimetic mucosal folds or constricted passages preceding a tumour site. Colour bar indicates ultrasound signal. **d**, Ultrasound imaging (superimposed time sequence) of the time-lapse locomotion of a DMCG swarm in a filled mouse bladder (C57BL/6 mice). Colour bar indicates ultrasound signal. **e**, In vivo validation of DMCG swarm navigation in an unfilled rat bladder (SD rat). Colour bar indicates ultrasound signal. **f**,**g**, Fluorescence image sequence (**f**) and quantification of the TFI (**g**) over time for high-concentration free-ICG solution versus PBS-suspended DMCGs loaded with an equivalent-dose ICG instilled into the mouse bladder, comparing DMCG-induced convective diffusion with free-ICG passive diffusion. Colour bar indicates radiant fluorescence (**f**). Data represent mean ± s.d. (*n* = 3 biologically independent mice); error bands indicate s.d. **h**, Segmentation of DMCGs in the mouse bladder tumour model. TR, tumour region; N-TR, non-tumour region. **i**, Autonomous navigation, tumour targeting and mode switch of DMCG swarm in the bladder tumour model. Convective flows are captured by Doppler ultrasound imaging. Colour bar indicates Doppler flow velocity. Illustration in **a** created in BioRender; Lin, L. https://BioRender.com/kfr9u4q (2026). Source data

Having established the controlled actuation, release and barrier penetration of DMCGs, we validated it in an image-feedback intravesical delivery workflow, before which its low cytotoxicity and ultrasound contrast was also verified (Supplementary Figs. 34 and 35). We implemented a robotic magnet system (RMS) in which our devised nRSM was mounted for deep learning-based closed-loop control of DMCG navigation and local drug release in bladder-mimetic models (Fig. 5a, Supplementary Figs. 36–40 and Supplementary Table 3). In a hydrogel bladder tumour phantom, DMCGs formed a stable swarm, navigated to the target site and were then switched to local actuation for convective release (Fig. 5b and Supplementary Video 10). Using preinstilled indocyanine green (ICG) as a model cargo, this strategy was found to increase the penetration depth by 3.6 fold within 40 min relative to passive diffusion alone (Supplementary Figs. 41 and 42). The DMCG swarms could also be redirected for retrieval after treatment.

**Extended Data Fig. 3:**
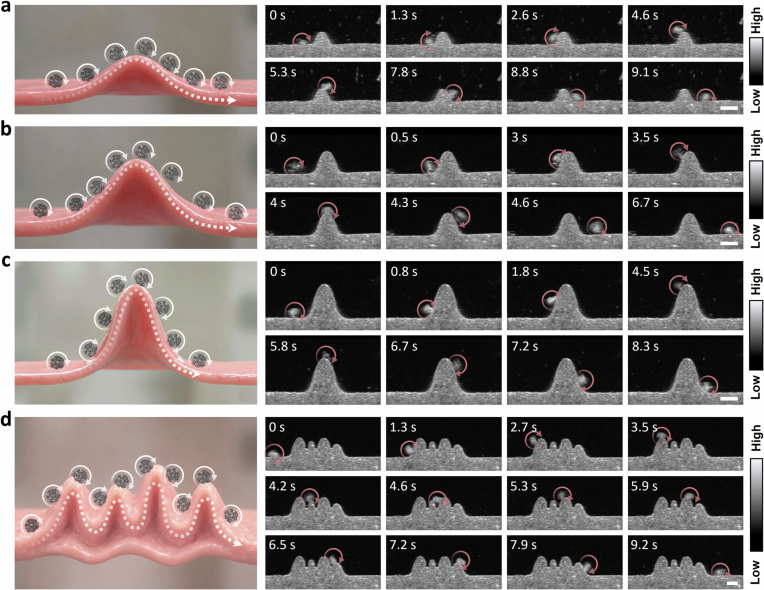
DMCG swarm locomotion in bladder phantoms of different folding morphologies. **a-d**, Schematic illustration (left) and time-lapse ultrasound images (right) of the DMCG swarm locomotion across (**a**) low-rise fold, (**b**) medium-rise fold, (**c**) high-rise fold, and (**d**) rugae-mimetic folds. Red arrows indicate the rolling direction of the swarm. Scale bar: 5 mm. Under real-time ultrasound monitoring, the DMCG swarms traversed all tested folds within *ca*. 10 s. During the process, no interruption, jamming, or trajectory deviations were observed.

**Extended Data Fig. 4:**
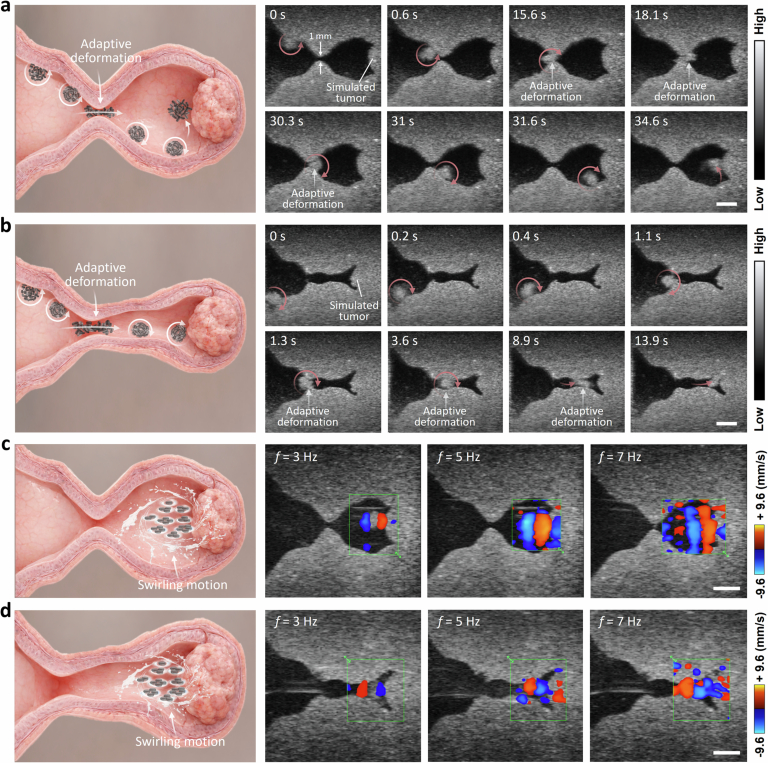
DMCG swarm navigation and swirling motion in highly-folded bladder tumor phantoms. Schematic illustration (left) and corresponding ultrasound images (right). **a**, DMCG swarm squeezing through a fold-induced constricted neck preceding the tumor site. **b**, DMCG swarm navigating a fold-induced confined cavity toward the tumor site. Red arrows indicate the rolling direction of the swarm. **c–d**, Representative Doppler ultrasound images illustrating the swirling motion and induced flow fields of the DMCG swarm within the phantom under different rotation frequency of the magnetic field (3, 5 and 7 Hz). Scale bar: 5 mm. Ultrasound imaging shows that when passing through exceedingly constricted passages *ca*. 1 mm in diameter, the DMCG swarms can smoothly traverse via adaptive deformation. Furthermore, the swarm motion states and flow-field distributions under different magnetic field frequencies can be monitored in Doppler ultrasound mode.

The performance of DMCGs in more complex lumen geometries designed to mimic bladder folding at different filling states was also examined. Under real-time ultrasound monitoring, DMCG swarms smoothly traversed various low-, medium- and high-rise folds, as well as rugae-like undulating surfaces, within approximately 10 s and without interruption or loss of control (Fig. 5c (left) and Extended Data Fig. 3). In tumour phantoms containing either a constricted passage (~1 mm in diameter) or a confined cavity, the swarms adapted their morphology to pass through narrow openings and remained detectable by Doppler ultrasound during actuation (Fig. 5c (right) and Extended Data Fig. 4). These experiments support the feasibility of image-feedback swarm navigation and localized drug release in geometrically constrained intravesical environments.

## In vivo tumour targeting and preclinical therapy

**Extended Data Fig. 5:**
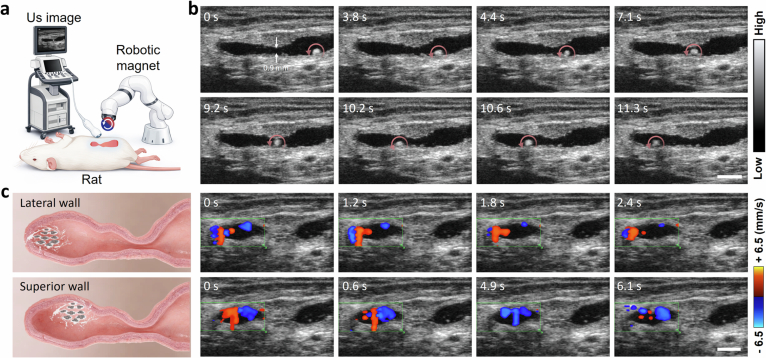
*In vivo* ultrasound monitoring of RMS-guided DMCG navigation and swirling motion in unfilled rat bladders. **a**, Schematic illustration of the robotic magnet system and DMCG manipulation in the rat bladder under ultrasound monitoring. **b**, Time-lapse ultrasound images illustrating the locomotion of DMCG swarm through a narrow, unfilled bladder cavity. Red arrows indicate the rolling direction of the swarm. **c**, Doppler ultrasound images illustrating localized swirling motion of the DMCG swarm at targeted regions of the bladder, including the lateral and upper bladder walls, respectively. Scale bar: 5 mm. Under guidance of the RMS magnetic control system, DMCG swarms could navigate the narrow unfilled bladder cavity, successfully traversing a *ca*. 0.9 mm narrow region and switching to swirling motion after reaching the target region. Illustration in **a** created in BioRender; Lin, L. https://BioRender.com/kfr9u4q (2026).

Next, we evaluated DMCG delivery in vivo under ultrasound image-feedback autonomous control. After transurethral instillation, DMCG swarms could be assembled and steered within a filled mouse bladder (Fig. 5d), and remained controllable in the more constrained cavity of an unfilled rat bladder (Fig. 5e), where they traversed narrow regions and switched to localized swirling at the target site (Extended Data Fig. 5a,b). Doppler ultrasound time-lapse imaging demonstrated localized stability, regional selectivity (lateral or upper) and prominent flow-field distributions of the swarm during swirling motion (Extended Data Fig. 5c). These findings establish that DMCGs remain controllable in living bladder environments across different cavity states.

**Extended Data Fig. 6:**
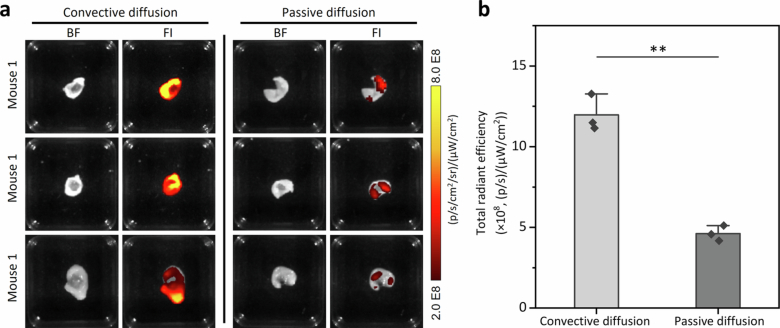
ICG fluorescence of incised tissues of ICG-loaded DMCG-treated mouse bladder (convective diffusion) vs free-ICG treated bladder (passive diffusion) after PBS flushing. **a-b**, Fluorescence imaging (**a**) and quantification of the total fluorescence intensity (**b**). Data are mean ± s.d. (n = 3 biologically independent mice); error bars indicate s.d. P values were determined by two-tailed Welch’s t-test (P = 0.00317). *P ≤ 0.05, **P ≤ 0.01, ***P ≤ 0.001. The bladder tissue was excised and thoroughly rinsed with PBS to remove unbound luminal ICG, after which the DMCG group still exhibited significantly higher residual fluorescence intensity compared with the free-ICG group. Quantitative analysis shows that the total fluorescence intensity of the convective diffusion group is 259% that of the passive diffusion group. Source data

**Extended Data Fig. 7:**
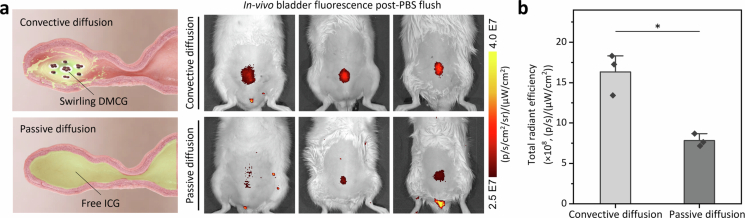
*In vivo* fluorescence of DMCG convection–enhanced drug diffusion in unfilled rat bladders. **a**, Schematic illustration (left) and in vivo fluorescence images (right) of the residual ICG distribution in the bladder after 30 min of DMCG–induced convective diffusion and passive diffusion of a free ICG solution, followed by PBS rinsing. **b**, Quantitation of the total fluorescence intensity in the two groups. Data are mean ± s.d. (n = 3 biologically independent mice); error bars indicate s.d. P values were determined by two-tailed Welch’s t-test (P = 0.0218). *P ≤ 0.05, **P ≤ 0.01, ***P ≤ 0.001. The results for unfilled rat bladders here are consistent with the ICG test for filled mouse bladders, presenting a total fluorescence intensity in the experimental group ca. 208.5% of that in the control group. Source data

**Extended Data Fig. 8:**
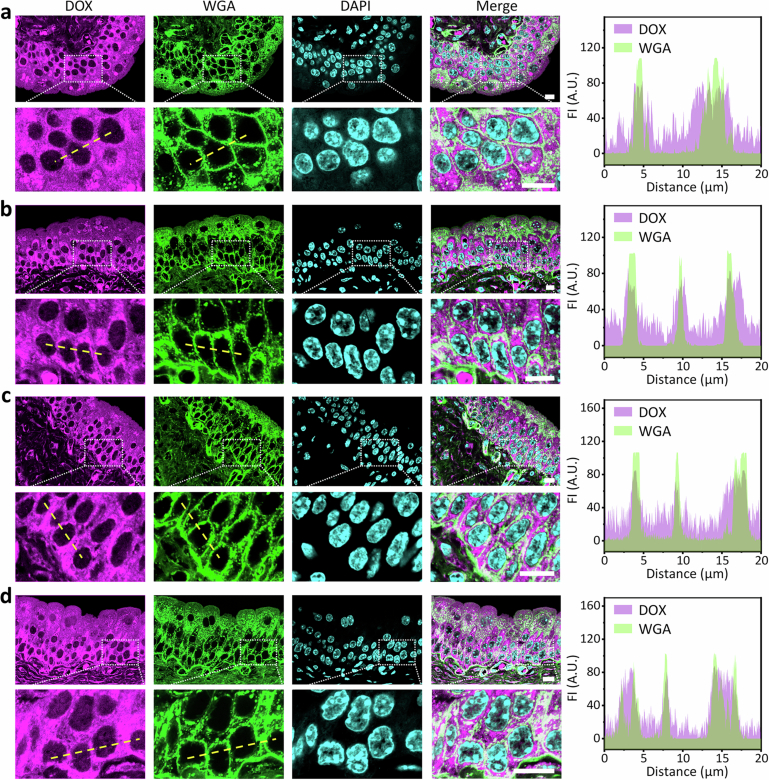
Microscopic fluorescence distribution and line-scan quantification of DMCG-facilitated DOX permeation across urothelial layers. (**a–d**, left) Overview of the urothelium (top) and high-magnification view of the intermediate layer (bottom). From left to right, single-channel fluorescence images of DOX (red), WGA (green), DAPI (blue), and the merged images, respectively. (**a–d**, right) Line-scan fluorescence intensity profiles along the region indicated by the yellow dashed line. Representative images from four independently prepared samples with similar results. Scale bar: 10 μm. DOX turns out to present a layer-dependent distribution in the urothelium with prominent signals in the umbrella and intermediate layers, but relatively low intensity in the basal layer. Further intra-layer analysis indicates that in the umbrella layer, the fluorescence intensity of DOX shows no significant difference between the apical membrane region and the underlying cytoplasm with an overall diffusive distribution. Therefore, even with coverage by the glycosylated barrier, the drug still appears to form a continuous diffusion state within the umbrella layer, providing desired initial conditions for further cross-layer diffusion. Source data

Whether DMCGs could enhance intravesical diffusion in vivo was also examined. In mice, bladders receiving ICG-loaded DMCGs showed a faster rise in fluorescence than bladders receiving an equal dose of free ICG, and the total fluorescence at 2 h reached 247.8% of that in the free-ICG group after rinsing to remove unbound luminal dye (Fig. 5f,g, Supplementary Fig. 43 and Extended Data Fig. 6). A similar advantage was observed in unfilled rat bladders after aspiration and rinsing (Supplementary Fig. 44 and Extended Data Fig. 7), indicating that convection-enhanced transport is maintained under physiologically constrained conditions. Likewise, DOX-loaded DMCGs increased urothelial permeation in mouse, where fluorescence imaging (Extended Data Fig. 8, left) and line-scan quantification (Extended Data Fig. 8, right) demonstrated local DOX prominence at the intercellular junction interface between cell membranes, suggesting that permeation may not be confined solely to transcellular routes because the auxiliary contribution of paracellular pathways cannot be excluded. Across all histological evaluations, no disruption of urothelial structure or obvious morphological damage were observed, supporting the DMCG-mediated delivery strategy for maintained urothelial structural integrity and enhancing interfacial drug delivery and tissue permeation efficiency as a non-invasive approach.

**Fig. 6:**
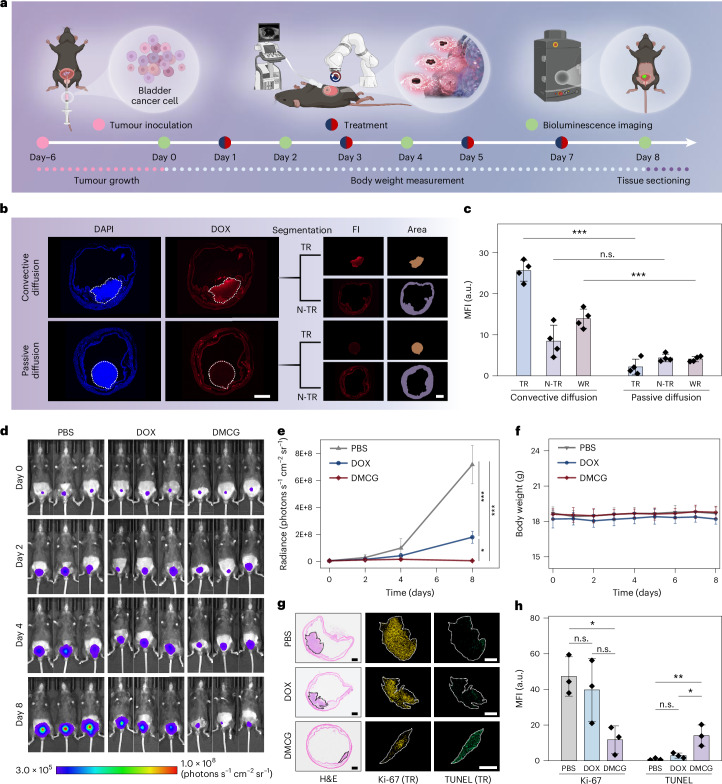
In vivo bioluminescence imaging and histological evaluation of 1-week preclinical intracavitary chemotherapy. **a**, Schematic of the preclinical protocol, in which tumour inoculation (pink dot), bioluminescence imaging (green dots) and treatment (blue/red dots) procedures are marked on designated days. **b**,**c**, Fluorescence images (**b**) and quantification of the mean fluorescence intensity (MFI) of DOX permeation in bladder tumour tissue slices after one DMCG treatment (**c**), corresponding to the preliminary in vivo evaluation conducted earlier (Fig. 5i) before the 1-week therapy. Scale bars, 1 mm. Data are presented as mean ± s.d. (*n* = 4 biologically independent mice); error bars indicate s.d. *P* values were determined by two-way repeated-measures ANOVA with Šidák’s multiple comparisons test. (TR, *P* < 0.0001; N-TR, *P* = 0.0659; whole-bladder region (WR), *P* < 0.0001). **d**, Representative bioluminescence images on days 0, 2, 4 and 8 of the PBS (blank), DOX (control) and DMCG (experimental) groups. **e**, Quantitative analysis of the bioluminescence intensity observed in **d**. Data are presented as mean ± s.d. (*n* = 3 biologically independent mice); error bars indicate s.d. *P* values were determined by two-way repeated-measures ANOVA with Šidák’s multiple comparisons test. On day 8, pairwise comparisons showed significant differences among all the treatment groups (PBS versus DOX, *P* = 0.0133; PBS versus DMCG, *P* < 0.0001; DOX versus DMCG, *P* < 0.0001). **f**, Body weight variation of mice during the treatment period. Data are presented as mean ± s.d. (*n* = 3 biologically independent mice); error bars indicate s.d. **g**, Haematoxylin and eosin (H&E) staining of the whole-bladder region (WR), Ki-67 immunofluorescence staining and TUNEL staining in the tumour region (TR) of the different groups. Scale bars, 500 μm. **h**, Quantitative analysis of Ki-67 and TUNEL fluorescence intensity among the PBS, DOX and DMCG groups. Data are presented as mean ± s.d. (*n* = 3 biologically independent mice); error bars indicate s.d. *P* values were determined by one-way ANOVA with Tukey’s multiple comparisons test. Ki-67: PBS versus DOX, *P* = 0.7621; PBS versus DMCG, *P* = 0.0341; DOX versus DMCG, *P* = 0.0827. TUNEL: PBS versus DOX, *P* = 0.7625; PBS versus DMCG, *P* = 0.0087; DOX versus DMCG, *P* = 0.0189. Statistical significance in **c**, **e** and **h** is indicated by *P ≤ 0.05, **P ≤ 0.01, ***P ≤ 0.001. Illustration in **a** created in BioRender; Lin, L. https://BioRender.com/kfr9u4q(2026). Source data

Using an orthotopic bladder tumour model, we then evaluated DMCG’s performance of tumour targeting and tissue permeation. Under ultrasound guidance, DMCGs were guided to the tumour in the locomotion mode and switched to the swirling mode for localized actuation, generating strong local Doppler flow signals with notable secondary flows of ~3.8 mm s^−1^ (Fig. 5h,i and Supplementary Video 11); the same protocols were implemented later for a preclinical therapy (Fig. 6a). Histological analysis showed substantially stronger and tumour-selective DOX accumulation with DMCG-assisted delivery than free DOX, with mean fluorescence intensity increasing by 1,083.6% in tumour regions and the tumour-to-non-tumour fluorescence ratio increasing from 0.56 to 3.6 (Fig. 6b,c and Supplementary Figs. 45–47). These animal data evidence that image-feedback DMCG actuation can enhance in vivo diffusion, enable localized tumour targeting and boost tissue permeation within the bladder.

Finally, we examined whether the drug gains could translate into therapeutic benefit with a 1-week preclinical therapy using four alternate-day intravesical treatments (Fig. 6a). Serial bioluminescence imaging showed a marked suppression of tumour burden in the DMCG group relative to phosphate-buffered saline (PBS) and free DOX (Fig. 6d,e). By the end of treatment, bioluminescence in the DMCG group was reduced to 0.59% of the PBS group and 2.36% of the free-DOX group, suggesting a more than 40-fold increase in therapeutic efficacy. Throughout the therapy, the animal body weight remained stable, and subsequent major-organ histology and serum biochemistry showed no evident treatment-related toxicity (Fig. 6f and Supplementary Figs. 49 and 50). Haematoxylin and eosin staining revealed minimal residual tumour after DMCG treatment, whereas sizeable tumours persisted in the control groups (Fig. 6g, left). DMCG-treated tumours also showed increased terminal deoxynucleotidyl transferase dUTP nick end labelling (TUNEL) staining and reduced Ki-67 staining, consistent with enhanced apoptosis and reduced proliferation (Fig. 6g,h and Supplementary Fig. 48). Together, these data support a therapeutic advantage of DMCG-assisted intravesical delivery without detectable systemic toxicity under the tested conditions.

## Conclusion

In the present study, we have devised a machine-intelligent biohybrid microrobot of nanoporous channels with a flow-responsive hydrodynamic switch for focused drug delivery across biological barriers, and demonstrated the intravesical therapy of malignant bladder tumour. By enabling image-feedback closed-loop magnetic control in vivo through the simultaneous processing of high noise-to-signal ratio biomedical images with robust deep learning-based segmentation and detection algorithms, precise tumour targeting and ultrafast drug penetration is realized owing to the multimodal control and convective transport capability of the microrobots. Their gentle rolling/tumbling modes featuring low-level flow disturbance can be applied for the fast transport of drug payloads with minimal leakage, whereas the rotating/spinning mode featuring strong rotational flows can be applied for localized drug release once they arrive on the target lesion site.

The outcome of our study represents a practical advance in intravesical therapy, which often falls short in selective lesion targeting or effective barrier penetration and is typically of minimal synergistic benefits due to time-limited passive diffusion. Owing to its unique hierarchical micro-nanostructure, the magnetic algebot DMCG as a novel drug delivery system overcomes existing limitations and substantially improves the precision as well as efficacy of intravesical therapy. A comparative analysis with conventional strategies for treating bladder cancer shows the superior performance of DOX-loaded DMCGs (Supplementary Table 4) versus techniques such as radio-frequency-induced thermochemotherapy^49^, electromotive drug administration^50^, dimethyl sulfoxide^51,52^ and various nanocarriers^53–55^ or nanomotors^28,56–59^, where DMCGs exhibit a substantial improvement. In particular, DMCGs enhance the overall drug permeation in the whole bladder by 242.3% and the tumour-region-specific permeation by 1,083.6% with only 30-min treatment. This, by far, surpasses the performance of common strategies, for example, 173%, 943% by radio-frequency-induced thermochemotherapy over 1 h and 742%, 120% by DOX-nanocarrier over 72 h. This superior efficacy of DMCGs is attributed to its targeted delivery and controlled release capabilities absent in other approaches. Furthermore, DMCG stands out as a non-invasive therapeutic agent as the treated urothelium remains intact and no mechanical penetration, tissue puncture, luminal tearing or histological damage is involved in the process of convection-enhanced transurothelial drug transport. Our findings, therefore, imply the potential of DMCGs as a non-invasive therapy for bladder cancer.

Current clinical paradigm of bladder cancer therapy is primarily the transurethral resection of the tumour followed by intravesical immunotherapy or chemotherapy instillation. However, critical challenges arise for this protocol for patients suffering from severe diabetes or deficient immunity. The algebot-enabled ultrafast convective chemotherapy reported here may be considered a conservative treatment for such patients to inhibit the rapid progression of bladder tumour without risking surgery complications. For patients undergoing transurethral resection, our strategy can still contribute as an auxiliary treatment modality to reduced operation window or incidents by enhancing the selectivity and efficacy of chemotherapy with controllable intravesical dosing and obeying the normal or shortened voiding cycles. To realize the translation of the proposed strategy, we will focus on refining automated image feedback to simultaneously tune the real-time navigation and controlled drug release of DMCG under complex physiological conditions. Future research should also include developing high-anisotropy nanoparticles, novel magnetic composites and/or hierarchical structural designs to elevate the robustness and precision of actuation and control of algebot variants for on-demand intracavitary chemotherapies in different organs beyond the bladder (for example, abdominal and uterine environments). Furthermore, we would delve into the swarming behaviour of algebots for embedded physical intelligence and the mechanistic evolution of convective drug diffusion for providing solid theoretical basis to optimize the drug delivery strategy. We will also evaluate the pharmacokinetics and longitudinal therapeutic outcome of such intravesical therapy, and monitor potential side effects using bladder tumour models of larger animals (for example, rabbits and piglets) incorporating live imaging and histological assessments. These subsequent efforts will lay a solid foundation for the clinical translation of algebot-based intracavitary chemotherapy, thereby stepping closer towards a future in which efficient and minimally invasive therapy provides versatile stratified medicine for cancer care.

## Methods

### Fabrication of MCG and DMCG

*C. granii* species were sourced from the Freshwater Algae Culture Collection, and then cultivated in 1-l flasks with artificial seawater enriched using f/2-Si media (pH 8.3) and maintained at 23 °C under a 12-h light/12-h dark cycle with an illuminance of 2,000 lx. After collecting, *C. granii* cells were sieved through a 75-μm nylon mesh and washed with deionized water. The filtered cells were exposed to a 1-M HCl solution at a ratio of 2 mg ml^−1^ for 12 h. The acid-treated *C. granii* was then sieved, washed with deionized water and introduced into the Fe_3_O_4_ NP suspension (3 mg ml^−1^) at a ratio of 1 mg ml^−1^. After 12 h of incubation at room temperature with gentle agitation, the mixture underwent sieving and washing processes for purified MCG. The prepared MCGs were mixed with a DOX/Tris-HCl solution (1 mg ml^−1^, pH 8.4) at a ratio of 0.5 mg ml^−1^ and gently shaken for 12 h for drug loading. Subsequently, dopamine was added to the solution at a ratio of 2 mg ml^−1^, and the mixture was shaken for 4 h to facilitate the formation of a PDA coating on the surface of MCG, yielding DOX-loaded DMCGs.

### Magnetic actuation and motion analysis of DMCGs

There are two magnetic actuation systems in this study. The first is a custom-built tri-axial Helmholtz coil system equipped with a customized coil-driving device for programmable RMFs to characterize the motion of DMCGs in distinct regimes (Supplementary Sections 3 and 4). The second is an nRSM setup designed in-house for flexible operation in a larger workspace and versatile swarm control in vivo (Supplementary Section 8). The motion and swarming behaviour of DMCGs was observed and recorded using a camera (MER-503-36U3C, Daheng Imaging). ImageJ plug-in Trackmate was used to analyse the motion of DMCGs. The flow velocity field induced by DMCG motion was measured using PIV (Supplementary Section 11).

### Numerical simulation of fluid flow and solute transport

Simulation studies were performed using COMSOL Multiphysics 6.1 (Supplementary Section 12). The fluid flow generated by DMCG motion was modelled using the laminar flow model (equations (25)–(27) in Supplementary Section 13). To account for the nanoporous structure of DMCG, the laminar flow model was coupled with a porous media model (equations (28)–(31) in Supplementary Section 13). Drug release and penetration induced by DMCG rotation were simulated using a convective diffusion model (equations (32) and (33) in Supplementary Section 13) and its variant for porous media (equations (34) and (35) in Supplementary Section 13). The magnetic field was modelled according to equation (36) in Supplementary Section 13. The rotational motion of DMCG was implemented using the dynamic mesh method for rotating domains in COMSOL.

### Drug release experiment with DOX-loaded DMCG

Here 5 ml of PBS (pH 7.4) was placed in a glass beaker, maintained at 25 °C under ambient conditions. DOX-loaded DMCGs (5 mg, DOX concentration of 100 μg ml^−1^ in suspension) were introduced and gently dispersed to obtain a homogeneous suspension. For single-DMCG-induced release, the coil-based magnetic actuation system (Supplementary Fig. 3) was used to generate RMFs (denoted as B_1_–B_4_), inducing four distinct motion modes of individual DMCGs. For each motion mode, the actuation frequency was set to 0, 1, 3 or 5 Hz and identical magnetic flux density was maintained. At designated time points (5, 10, 15, 20, 25 and 30 min), DMCGs were magnetically sedimented to the bottom of the container and 200 μl of supernatant (4% of the total release volume) was withdrawn under light-protected conditions to minimize DOX photodegradation. An equal volume of fresh PBS was immediately replenished to maintain a constant release volume and approximate near-sink conditions. All experiments were performed with *n* = 3 independent samples. Absorbance of the collected supernatant samples was measured at 488 nm using a multifunctional microplate reader (SPARK, Tecan). For DMCG-swarm-induced release, experiments were conducted under identical DMCG mass, loading concentration, buffer volume and environmental conditions using the RMS (Supplementary Figs. 36 and 37). The swarms were actuated in the locomotion (1 Hz) and swirling (5 Hz) regimes corresponding to the motion classifications defined above. Supernatant aliquots were collected at 1, 5, 10, 20 and 30 min following the same sampling and calculation protocol described for the single-body assays.

### Preparation of hydrogel matrix and tumour spheroid

The hydrogel matrix and tumour spheroid are intended as two physical mimics of bladder tumour barriers. A 5% agarose solution was prepared and microwaved for 1 min to ensure complete dissolution. Subsequently, 2 ml of the solution was pipetted into each well of a 12-well plate. Twelve cylindrical plastic moulds, each with a diameter of 1 mm, were inverted and gently pressed into the agarose solution in each well to a depth of 1 mm. The plates were then allowed to stand for 1 h to ensure complete hydrogel solidification. Once solidified, the moulds were removed to yield a hydrogel matrix containing microwells. The hydrogel was then immersed in an excess of PBS solution and weighed every 10 min to monitor water uptake. Saturation was confirmed when the weight of the hydrogel stabilized, and then it was deemed ready for the drug penetration experiments. If saturation was not achieved with one attempt, the hydrogel was reimmersed into PBS to repeat the previous process. For the preparation of tumour spheroids, each well of a 96-well plate was filled with a 1% agar solution. MTB-2 G3 cells (5,000–8,000 per well) were added, and after centrifugation at 300*g* for 5 min, the plate was incubated for approximately 4 days to promote the formation of tumour spheroids.

### In vitro assessment of drug penetration in bladder-barrier mimic matrices

Convective drug penetration in hydrogel microwell: the hydrogel microwell was exposed to an RMF (**B**_*t*3_ = 10 mT, *f*_r_ = 0 and 3 Hz; Supplementary Section 4), and was filled with a 30-μl mixture containing 0.1 mg ml^−1^ of MCG and 100 μg ml^−1^ of DOX. Specific fluorescence signals of DOX were then captured using a fluorescence microscope, and images were captured within an interval of 30 s to assess DOX diffusion. Convective drug penetration in tumour spheroid: 3 ml of cell culture medium containing a singular tumour spheroid was first added to a Petri dish, which was kept still for 6 h under cell culture conditions to facilitate adhesion of the tumour spheroid to the substrate. Subsequently, the culture medium was removed and the Petri dish was exposed to the same magnetic field used in the hydrogel microwell test (**B**_*t*3_ = 10 mT, *f*_r_ = 0 and 3 Hz). Then, 1 ml of a mixture containing MCG and DOX, with a mass-to-volume ratio of 0.2 mg ml^−1^ (100 μg ml^−1^), was introduced into the Petri dish. The subsequent observation of DOX diffusion in tumour spheroids followed the protocol used in the above hydrogel experiments.

### In vitro assessment of drug delivery in artificial bladder tumour model

The preparation of the hydrogel bladder tumour model used a protocol similar to that of the hydrogel microwell demonstrated above. Here the cylindrical moulds were replaced with three-dimensional (3D) printed bladder tumour moulds. A 5% agarose solution was heated and poured into a container that can embed the tumour moulds. Right after pouring, the tumour moulds were slowly inserted into the agarose solution, until they achieved good stability and full immersion. After complete solidification of the hydrogel, the moulds were gently removed to create a hydrogel cavity with a hump, which was intended to mimic the bladder tumour. The as-prepared hydrogel was then immersed in an excess of PBS solution, and its weight was monitored periodically to make the hydrogel absorbance reach saturation before being used for subsequent experimental procedures. The DMCGs were prepared by mixing MCGs with an ICG solution (100 μg ml^−1^), using a mass-to-volume ratio of 0.5 mg ml^−1^. In the experimental group, 2 ml of the aforementioned suspension was introduced into the hydrogel-mimicking bladder tumour model. An RMS integrating our in-house permanent magnet nRSM was utilized to gather the ICG-loaded MCG swarm at the urethral orifice of the artificial bladder, guiding them along the bladder wall to the simulated tumour region in a controlled scrolling motion. The magnetic field pattern was then altered to induce swirling of the DMCG swarm in the tumour area, which was sustained for 40 min before actively retrieving the DMCG swarm from the model bladder using the RMS. The entire process was monitored and recorded under an in-house near-infrared region II imaging platform. In the control group, 2 ml of ICG solution (100 μg ml^−1^) was added to the hydrogel bladder tumour model, and the passive diffusion process was documented over 40 min.

### Ultrasound-guided DMCG actuation in folded/constrained bladder phantoms

Folded bladder-mimetic hydrogel phantoms were fabricated using 3D printed moulds containing predefined low-, medium- and high-rise fold as well as rugae-mimetic undulating geometries and constricted passage structures (minimum constriction, ~1 mm). Agarose hydrogel (4% w/v) containing silica microparticles (0.5% w/v, 10-μm diameter) was prepared by first dispersing silica particles in deionized water under vigorous stirring to obtain a homogeneous suspension. Agarose powder was then added to the suspension, and the mixture was heated to 95–100 °C with continuous stirring until the complete dissolution of agarose was achieved. The resulting particle-laden solution was poured into 3D printed moulds and allowed to cool to room temperature for gelation. DMCG swarms were introduced into the phantom lumen and visualized under real-time B-mode ultrasound imaging. The RMS was applied to actuate and guide the swarm across the folded geometries and narrow-neck constrictions under continuous imaging guidance. To assess flow disturbances generated during actuation, Doppler ultrasound imaging was performed at magnetic actuation frequencies of 1, 3 and 5 Hz. Localized Doppler signal intensity within folded and confined geometries was recorded to evaluate flow generation under spatially restricted conditions.

### Housing conditions of mice and rats and ethical approval for animal experiments

Female C57BL/6 mice and female Sprague–Dawley (SD) rats were housed in a specific-pathogen-free facility under a 12-h light/12-h dark cycle, with controlled ambient temperature (22 ± 2 °C) and relative humidity (50%–60%). Food and water were provided ad libitum. All animal procedures described in subsequent sections were approved by the Animal Care and Use Committee of Xiamen University (accreditation numbers XMULAC20190065 and XMULAC20250009) and conducted in accordance with institutional guidelines.

### Ultrasound-guided DMCG navigation in mouse and rat bladders

Female C57BL/6 mice (6 weeks old) and female SD rats (12 weeks old) were anaesthetized with isoflurane delivered via a gas anaesthesia system and positioned supine on a high-frequency ultrasound imaging platform (Vevo 2100, VisualSonics), with limbs secured to minimize motion artefacts. Following sterile preparation, a 24-G catheter was transurethrally inserted into the bladder lumen. A suspension of DMCGs in PBS (0.1 mg ml^−1^) was instilled intravesically (50 μl). Real-time ultrasound imaging was used to visualize the bladder cavity and monitor the distribution of DMCGs. The RMS was applied to actuate and control DMCGs along the urothelial surface under imaging guidance. Rat experiments were performed using the same anaesthesia protocol, catheterization procedure, magnetic actuation parameters and ultrasound guidance workflow as for mice, unless otherwise specified.

### In vivo evaluation of drug diffusion in mouse and rat bladders

ICG-loaded DMCG (choice of ICG rather than DOX for in vivo fluorescence is detailed in Supplementary Section 24) was prepared according to the above-mentioned loading procedure by incubating 0.50 mg of MCG in 1.0 ml of ICG solution (200 μg ml^−1^), yielding a drug loading of 22.8% (w/w). Unloaded free ICG in the suspension was removed by magnetic separation, and the product was lyophilized. For the in vivo administration of mouse bladders, mice were randomly assigned to the passive diffusion group or the convective diffusion group (*n* = 3). Each mouse in the passive diffusion group received 50 μl of free-ICG solution (200 μg ml^−1^), corresponding to an ICG dose of 10 μg per mouse. To ensure equal ICG dose between groups, each mouse in the convective diffusion group received 43.8 μg of DMCG dispersed in 50 μl of PBS. All samples were instilled into the mouse bladder via the urethra. An RMF (3 Hz) was applied over the bladder centre in the convective diffusion group using the RMS system; no field was applied in the passive diffusion group. Fluorescence distribution in the bladder region was monitored using an in vivo imaging system (Lumina III 2, PerkinElmer) at 0, 5, 10, 15, 20, 30, 40, 60, 100 and 120 min. Immediately after completion of the imaging procedure, the bladder tissues were excised, opened longitudinally and thoroughly rinsed with PBS to remove unbound ICG remaining in the bladder lumen, followed by ex vivo fluorescence imaging of the collected tissues. For in vivo administration of SD rats (*n* = 3 per group), the anaesthesia, catheterization, dosing strategy and magnetic actuation protocol were identical to those used in mice. In vivo fluorescence imaging was performed at 0, 5, 10, 20 and 30 min. Immediately after imaging at 30 min, intravesical fluid or suspension was aspirated, and the bladder lumen was rinsed three times with PBS to remove residual drug within the cavity. In vivo fluorescence imaging was then repeated to assess retained and tissue-associated fluorescence signals.

### Construction of mouse bladder tumour model in vivo

Animals: female C57BL/6 mice, aged 6 weeks, were sourced from the Xiamen University Animal Experiment Center. Cells: MB49-LUC murine bladder cancer cells, labelled with luciferase, were utilized for the study. Mice were anaesthetized using 2% isoflurane at a flow rate of 1.5 l min^−1^ with oxygen. Once fully anaesthetized, the mice were positioned supine on a 37 °C heated pad. The lower abdomen was gently massaged to expel residual urine, followed by depilation and disinfection of the urethral orifice with povidone–iodine solution. A 24-G intravenous catheter was inserted through the urethra into the bladder, and the bladder was flushed with 50 μl of PBS using a 1-ml syringe, a process repeated three times. Subsequently, 50 μl of a PBS suspension containing 3 × 10^6^ MB49-LUC cells was injected into the bladder. Anaesthesia was maintained for 1 h. Two days post-procedure, the establishment of the tumour model was confirmed using small-animal fluorescence (Lumina III 2, PerkinElmer) and ultrasound imaging (Vevo2100, VisualSonics) systems. The maximum tumour size permitted by the institutional ethics committee was 10 × 10 × 10 mm^3^. Tumour size was monitored throughout the study, and this limit was not exceeded in any animal experiments.

### In vivo evaluation of drug penetration in mouse bladder tumour model

Female C57BL/6 mice bearing bladder tumours were randomly divided into convective diffusion and passive diffusion groups two weeks after tumour establishment. Here 50 μl free-DOX solution (800 μg ml^−1^, corresponding to an intravesical therapeutic dose of approximately 2 mg kg^−1^ based on mouse body weight) was designated as the passive diffusion group, whereas DMCG containing an equivalent DOX dose (40 μg) was assigned to the convective diffusion group. DMCG was prepared as described above by incubating 0.50 mg of MCG in 1.0 ml of DOX solution, yielding a drug loading of 27.95%. For in vivo administration, mice in the convective diffusion group (*n* = 4) received approximately 143 μg of DMCG dispersed in 50 μl of PBS, whereas mice in the passive diffusion group received the same volume of free-DOX solution. All formulations were instilled into the mouse bladder via the urethra. In the passive diffusion group, the catheter was sealed to retain the drug in the bladder for 2 h. The convective diffusion group underwent real-time ultrasound imaging guidance, during which a swarm of DMCG particles was directed towards the tumour site using the RMS under guidance of ultrasound imaging. The RMS end-effector switched the magnetic field mode to induce the swarm swirling under an RMF at 3 Hz for 30 min and then the DMCGs were retained for 1.5 h to match the same 2-h voiding cycle. After treatment, bladder contents from both groups were aspirated using a syringe and the bladder was rinsed three times with PBS. Mice were then euthanized, and bladders were collected and fixed. Frozen sections were prepared near the largest tumour surface and stained with DAPI. Fluorescence imaging of DAPI and DOX was performed using an Olympus VS200 automated slide scanning system, maintaining consistent exposure settings across all samples.

### Preclinical trial of intracavitary chemotherapy in mouse bladder tumour model

Bladder tumour models were established in mice as described previously. On day 6 post-tumour implantation (designated as day 0), tumour-bearing mice were randomly divided into three groups: PBS, DOX and DMCG (*n* = 3 per group). On days 1, 3, 5 and 7, mice were anaesthetized and intravesically instilled with the respective formulations, followed by the corresponding procedures. The DOX and DMCG groups received treatments identical to those of the passive diffusion and convective diffusion groups described in the aforementioned drug penetration assay in the bladder tumour model, respectively. The PBS group received 50 μl of PBS per treatment, with other procedures consistent with the DOX group. Mouse body weight was monitored daily throughout the treatment period to evaluate systemic toxicity. Bioluminescence imaging of bladder tumours was performed on days 0, 2, 4 and 8 using an IVIS Lumina III system (PerkinElmer). After the final imaging on day 8, bladder tissues were collected, fixed and sectioned for histological analysis to further evaluate the treatment efficacy. The tissue histology was assessed by haematoxylin and eosin staining, whereas tumour cell apoptosis and proliferation were assessed by fluorescence co-staining with DAPI nucleus staining (blue), Ki-67 immunostaining (yellow) and TUNEL assay (green).

### Statistics and reproducibility

Sample size: no statistical methods were used to predetermine sample sizes, but our sample sizes are similar to those reported in previous publications^57,58^. The group size for each experiment is indicated in the corresponding figure legends or the Methods.

Replication: all experiments were independently repeated at least three times with similar results, unless otherwise stated in the figure legends. Biological and technical replicates were distinguished where appropriate.

Data exclusions: no animals or data points were excluded from the analyses, unless explicitly noted.

Data distribution: for parametric statistical analyses, data distribution was assumed to be normal, but this was not formally tested. To allow the direct assessment of data distribution, individual data points are shown where applicable.

Randomization: animals were randomly assigned to experimental and control groups before treatment. Randomization was performed manually without dedicated randomization software. In vitro experiments did not involve allocation procedures requiring randomization.

Blinding: data collection and analysis were not performed blind to the conditions of the experiments. Potential bias was minimized by using prespecified, uniform acquisition and quantification settings, together with objective outcome metrics.

Statistical tests: all statistical tests were performed using GraphPad Prism 9.4. Two-tailed Welch’s *t*-tests were used for comparisons in Fig. 4j,n and Extended Data Figs. 6b and 7b. Two-way repeated-measures analysis of variance (ANOVA) with Šidák’s multiple comparisons test was used for Fig. 6c,e. One-way ANOVA with Tukey’s multiple comparisons test was used for Fig. 6h. Significance levels are denoted as *P ≤ 0.05, **P ≤ 0.01 and ***P ≤ 0.001.

### Reporting summary

Further information on research design is available in the Nature Portfolio Reporting Summary linked to this article.

## Online content

Any methods, additional references, Nature Portfolio reporting summaries, source data, extended data, supplementary information, acknowledgements, peer review information; details of author contributions and competing interests; and statements of data and code availability are available at 10.1038/s41565-026-02195-0.

## Supplementary information

Supplementary Information Supplementary Sections 1–26, Figs. 1–50, Tables 1–4, Algorithms 1–6 and Methods.

Reporting Summary

Supplementary Video 1 Workflow for applying machine-intelligent multimodal DMCGs towards efficient targeted intracavitary chemotherapy.

Supplementary Video 2 Magnetic control and motion regimes of a single DMCG.

Supplementary Video 3 Directed motion of a single DMCG along predefined paths.

Supplementary Video 4 Autonomous navigation and real-time path planning in complex mazes.

Supplementary Video 5 Multibody dynamics and controlled locomotion of multiple DMCGs.

Supplementary Video 6 Multimodal control and reconfigurable pattern of DMCG swarms.

Supplementary Video 7 Simulation of the flow field perturbed by single-body and multibody rotating DMCGs.

Supplementary Video 8 Simulation of the drug-release concentration field and convective/diffusive fluxes by single-body and multibody rotating DMCGs.

Supplementary Video 9 Simulation of the drug penetration concentration field and convective/diffusive fluxes by single-body and multibody rotating DMCGs.

Supplementary Video 10 In vitro assessment of drug release/penetration efficiency in an artificial bladder tumour model.

Supplementary Video 11 In vivo validation of intracavitary chemotherapy with DMCGs in a mouse bladder tumour model.

## Source data

Source Data Fig. 2 Source data for Fig. 2b,c.

Source Data Fig. 3 Source data for Fig. 3c–f,h.

Source Data Fig. 4 Source data for Fig. 4d–f,i,j,m,n.

Source Data Fig. 5 Source data for Fig. 5g.

Source Data Fig. 6 Source data for Fig. 6c,e,f,h.

Source Data Extended Data Fig. 1 Source data for Extended Data Fig. 1b–d.

Source Data Extended Data Fig. 2 Source data for Extended Data Fig. 2a–d.

Source Data Extended Data Fig. 6 Source data for Extended Data Fig. 6b.

Source Data Extended Data Fig. 7 Source data for Extended Data Fig. 7b.

Source Data Extended Data Fig. 8 Source data for Extended Data Fig. 8.

## Data Availability

All data supporting the findings of this study are available in the Article and its Supplementary Information. Source data are provided with this paper. Source data are also available via Zenodo at 10.5281/zenodo.20009871 (ref. ^59^).

## Code availability

Apart from algorithms and pseudocode made available in the Supplementary Information, custom code developed for the deep learning-based intelligent navigation framework is available at GitHub at https://github.com/linlin-rylynn/Machine-Intelligent-Multimodal-Algebot-for-Intracavitary-Chemotherapy.git.
