# Supplementary material for: Machine-intelligent multimodal algebot for intracavitary chemotherapy
Source: Nat Nanotechnol. 2026 Jun 22;21(7):996–1007. doi: 10.1038/s41565-026-02195-0 (PMC13379317; doi:10.1038/s41565-026-02195-0)
Supplement: Supplementary file 1 — Supplementary Sections 1–26, Figs. 1–50, Tables 1–4, Algorithms 1–6 and Methods. [file 41565_2026_2195_MOESM1_ESM.pdf]

nature nanotechnology

Supplementary information

https://doi.org/10.1038/s41565-026-02195-0

Machine-intelligent multimodal algebot for intracavitary chemotherapy

In the format provided by the authors and unedited

Supplementary Information

Supplementary Methods
Materials
Synthesis of Fe_3O_4 nanoparticles (NPs)
Structural characterization
Measurement of DOX release
Examination of DOX penetration

Supplementary Sections
Section S1. Structure characterization
Section S2. Drug loading efficiency
Section S3. Setup of coil-based magnetic control
Section S4. Programmable rotating magnetic field
Section S5. DMCG motion in biological fluids
Section S6. Locomotion control system
Section S7. Mechanical analysis of DMCG motion, multibody assembly and swarm control
Section S8. Setup and magnetic control of permanent-magnet system
Section S9. Characterization of swarming phase change conditions
Section S10. DMCG locomotion in complex environments
Section S11. Particle image velocimetry of DMCG motion
Section S12. Simulation model and parameters
Section S13. Governing equations of the simulation
Section S14. Fluid velocity and shear rate in DMCG nanopores
Section S15. Convection-enhanced drug release
Section S16. Convection-enhanced drug penetration into hydrogel
Section S17. Convection-enhanced drug penetration into tumor spheroid
Section S18. Interaction of DMCGs with simulated tumor interface
Section S19. Cytotoxicity assay
Section S20. Ultrasound signal intensity of DMCG
Section S21. Robotic magnet system with nRSM mounted
Section S22. Framework of image-feedback machine-intelligent control
Section S23. In vitro assessment of convection-enhanced drug penetration
Section S24. In vivo evaluation of convection-enhanced drug diffusion
Section S25. Evaluation of DOX permeation in bladder-tumor tissue sections
Section S26. Histological examination and biochemical analysis of treated tumor

Supplementary Figures
fig. S1. Supplementary structure characterization.
fig. S2. Characterization of drug loading efficiency for DMCG.
fig. S3. Coil-based magnetic control devices.
fig. S4. Schematic illustration of the programmable rotating magnetic field.
fig. S5. Motion of DMCG in biological fluids of various viscosities.
fig. S6. Diagram of the manual path-editing module for pre-defined locomotion control.
fig. S7. Diagram of automatic path-planning module for autonomous locomotion control.
fig. S8. Precision of DMCG navigation with the two control modules.
fig. S9. Configuration of the permanent magnet setup and measurement of its magnetic flux density at varying distances.
fig. S10. Experimental replication of the four DMCG motion regimes with the permanent magnet system.
fig. S11. Numerical simulation of the four magnetic field modes (B_{t1}, B_{t2}, B_{t3}, B_{t4}).
fig. S12. Multimodal magnetic control of a DMCG swarm for reconfigurable distribution.
fig. S13. Representative images of the parametric study on the DMCG swarm assembling patterns versus operating magnet distance (5–100 mm; 134.7–1.0 mT) and rotation frequency (1–5 Hz).

fig. S14. Heatmap of the DMCG swarm assembling strength versus operating magnet distance (5–100 mm) and rotation frequency (1–5 Hz).
fig. S15. Representative images of the parametric study on the DMCG swarm scrolling-locomotion patterns versus operating magnet distance (10–100 mm; 59.3–1.0 mT) and rotation frequency (0.5–5 Hz).
fig. S16. Transition phase diagram of the DMCG scrolling-locomotion states versus operating magnet distances (10–100 mm) and rotation frequency (0.5–5 Hz).
fig. S17. Representative images of the parametric study on the DMCG swarm swirling patterns versus operating magnet distances (40–120 mm; 8.4–0.4 mT) and rotation frequency (0–7 Hz).
fig. S18. Transition phase diagram of the DMCG swirling states versus operating magnet distance (40–120 mm) and rotation frequency (0–7 Hz).
fig. S19. Gravity-resisting locomotion of DMCG swarms.
fig. S20. Robust locomotion of DMCG swarms on complex 3D surfaces.
fig. S21. Particle image velocimetry (PIV) data of DMCG motion experiments.
fig. S22. Simulation results of the fluid velocity and shear rate in the nanopores of DMCG under four distinct motion modes.
fig. S23. Simulation of the drug concentration field and convective/diffusive fluxes across the surface of single-body and multi-body rotating DMCGs.
fig. S24. Quantification of simulated drug release and convective flux from single-body and multi-body rotating DMCGs.
fig. S25. Experiments of DMCG-mediated convective drug penetration into hydrogel.
fig. S26. Fluorescence intensity profile across the hydrogel wall along the yellow dashed line.
fig. S27. Simulation of the drug concentration field and convective/diffusive fluxes across the boundary of hydrogel wall in the presence of a five-body rotating DMCG assembly.
fig. S28. Quantification of the simulated drug-penetration quantity and convective flux in fig. S27.
fig. S29. Experiments of DMCG-induced convective drug penetration into tumor spheroids.
fig. S30. Fluorescence intensity profile across the tumor spheroid along the white dashed line.
fig. S31. Experiments of magnetically controlled DMCG clusters swirling near the tumor spheroid.
fig. S32. Demonstration of the non-adhesive behavior of DMCG clusters upon collision with the tumor spheroid surface.
fig. S33. Stability of DMCG swirling near a simulated tumor boundary under varying operating conditions.
fig. S34. Evaluation of DMCG cytotoxicity and its motion-induced mechanical damage.
fig. S35. Ultrasound signal intensity of DMCG at different concentrations.
fig. S36. Robotic magnet system (RMS).
fig. S37. Time-lapse sequence of the robotic magnet system (RMS) pose and gait for actuation and multimodal control of the DMCG swarm's reconfiguration and locomotion.
fig. S38. Workflow for machine-learning empowered intelligent control of DMCG microrobots for tumor targeting and drug delivery.
fig. S39. U-Net algorithm for ultrasound image segmentation.
fig. S40. Simulation of the magnetic potential scalar field arising from a rotating permanent magnet across an artificial mouse bladder model.
fig. S41. Assessment of DMCG-mediated drug penetration in a hydrogel-based artificial bladder-tumor model.
fig. S42. Fluorescence intensity profile of ICG penetrating across artificial bladder-tumor.
fig. S43. Supplementary fluorescence images of drug diffusion assessment in mouse bladder.
fig. S44. In vivo fluorescence imaging of ICG diffusion over 30 min and residual fluorescence after PBS rinsing in unfilled rat bladders.
fig. S45. Fluorescence imaging of DOX permeation in tumor-bearing mouse bladder tissues.
fig. S46. Segmentation of DOX fluorescence in tumor/non-tumor regions.
fig. S47. Quantification of DOX permeation in tumor-bearing mouse bladder tissues (based on DOX fluorescence data in fig. S45).
fig. S48. Histological analysis of mouse bladder tumor tissues after treatment.
fig. S49. H&E staining of main organs from the tumor-bearing mice after treatment.
fig. S50. Serum biochemical analysis of hepatic and renal function after the treatment.

Supplementary Tables
Table S1. The geometry construction and model configuration in COMSOL for different simulation scenarios.
Table S2. Main simulation parameters.
Table S3. D-H parameters of the robotic arm.
Table S4. Comparative analysis of strategies for enhancing drug penetration into bladder tumor tissues.

Supplementary Algorithms
Algorithm 1: Closed-loop control of ultrasound-guided navigation
Algorithm 2: Segmentation post-processing (UNet-based)
Algorithm 3: Microrobot and target detection (YOLO-based)
Algorithm 4: BFS-based path planning
Algorithm 5: Magnetic control execution I (Coil-based; image-frame control)
Algorithm 6: Magnetic control execution II (RMS; world-frame pure pursuit)

Supplementary Videos
Video S1. Workflow for applying machine-intelligent multimodal DMCG microrobots toward efficient targeted intracavitary chemotherapy.
Video S2. Magnetic control and motion regimes of single DMCG.
Video S3. Directed motion of single DMCG along predefined paths.
Video S4. Autonomous navigation and real-time path-planning in complex mazes.
Video S5. Multi-body dynamics and controlled locomotion of multiple DMCGs.
Video S6. Multi-modal control and reconfigurable pattern of DMCG swarms.
Video S7. Simulation of the flow field perturbed by single-body and multi-body rotating DMCGs.
Video S8. Simulation of the drug-release concentration field and convective/diffusive fluxes by single-body and multi-body rotating DMCGs.
Video S9. Simulation of the drug-penetration concentration field and convective/diffusive fluxes by single-body and multi-body rotating DMCGs.
Video S10. In vitro assessment of drug release/penetration efficiency in an artificial bladder-tumor model.
Video S11. In vivo validation of intracavitary chemotherapy with DMCG microrobots in a mouse bladder-tumor model.

Supplementary Methods

Materials

Ferric chloride ($FeCl_3$), ferrous chloride tetrahydrate ($FeCl_2 \cdot 4H_2O$), and hydrogen chloride (HCl) were sourced from Acros Organics (Thermo Fisher Scientific, USA). Sodium hydroxide (NaOH, ≥98%, pellets) was procured from Aladdin (China). Agarose was obtained from BioFroxx (China). Phosphate-Buffered Saline (PBS) and Fetal Bovine Serum (FBS) were procured from Gibco (Thermo Fisher Scientific Inc., USA). Artificial urine was purchased from ACMEC (China). Porcine stomach mucin (type III) was sourced from Sigma-Aldrich (USA). RPMI-1640 medium and DMEM/F12K medium were procured from iCell Bioscience (China). Penicillin/streptomycin and agarose were purchased from Shanghai Yuanye Biotechnology (China). The cell counting kit-8 (CCK-8) assay kit was acquired from Merck Life Science (USA). Doxorubicin (DOX), indocyanine green (ICG), and 4',6-diamidino-2-phenylindole (DAPI) were sourced from Aladdin (China). All chemical reagents were used without further purification.

Synthesis of Fe_3O_4 nanoparticles (NPs)

Fe_3O_4 NPs were synthesized via co-precipitation. In an ultrasonic bath (KQ500-DE, 40 kHz, 500 W/L), a 0.5 M NaOH solution was added dropwise to a 50°C mixture of $FeCl_3$ (0.008 M) and $FeCl_2$ (0.016 M), yielding a distinctive black suspension. Following a 60-minute sonication period, the suspension underwent three cycles of centrifugation (10,000 rpm, 15 min) and subsequent redispersion in DI water each time, resulting in positive-charged Fe_3O_4 NP suspensions.

Structural characterization

SEM images and EDX mapping were acquired using a field emission scanning electron microscope (SUPRA55 SAPPHIRECARL, ZEISS, Germany). XRD spectra were obtained with an X-ray diffractometer (XRD-7000, Shimadzu, Japan). Magnetic properties were measured using a vibrating sample magnetometer (7404, Lake Shore, USA). Fluorescence spectra were recorded using a multimode microplate reader (SPARK, Tecan, Switzerland). Bright-field and fluorescence microscopy images were captured with a fluorescence microscope (Observer7, Zeiss, Germany).

Measurement of DOX release

DOX concentration at each time point was determined from a pre-established absorbance–concentration calibration curve (**fig. S2A**), and the cumulative molar mass of released DOX was calculated accordingly. Cumulative drug release was corrected for dilution introduced by sequential sampling according to: $M_t = C_t V + \sum_{i=1}^{t-1} C_i V_s$, where M_t is the cumulative amount of DOX released at time t, C_t is the measured concentration at time t, V is the total release volume (5 mL) and V_s is the sampling volume (200 µL).

Examination of DOX penetration

To examine DOX penetration across the urothelial barrier, excised bladders of DMCG-treated mice were embedded, cryosectioned and subjected to staining with wheat germ agglutinin (WGA, green) to delineate the superficial umbrella cell layer and cell membranes, and DAPI (blue) to label nuclei. Sections were imaged using a laser scanning confocal microscope (LSM 980, Carl Zeiss AG, Oberkochen, Germany) equipped with a 40× objective. Three-channel fluorescence imaging was performed over the urothelial region using the following excitation/emission configurations: DAPI (excitation 405 nm, emission collected at 410–480 nm), WGA (excitation 488 nm, emission 490–520 nm) and DOX (excitation 546 nm, emission 550–650 nm). Sequential scanning with spectrally separated detection windows was applied to minimize channel crosstalk. Identical imaging parameters were maintained across all samples.

Section S1. Structure characterization

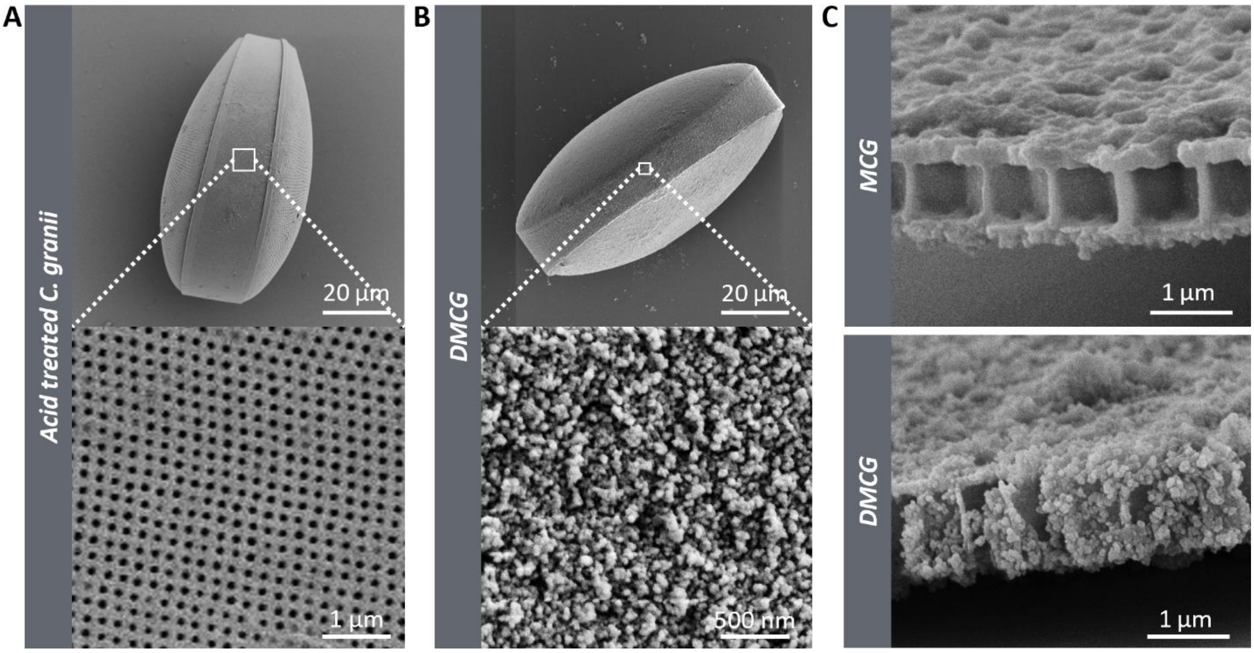

fig. S1. Supplementary structure characterization. Scanning electron microscopy (SEM) images of **(A)** acid-treated *C. granii* and **(B)** DMCG, with high-magnification views of the girdling band surface displayed on the bottom. **(C)** SEM images of the cross-sectional view of MCG and DMCG shell.

The supplementary data here complements the structure characterization presented in the main text (**Extended Data Fig. 1b**). The acid-treated *C. granii* shell show sieve-like nanopores on the front (**Extended Data Fig. 1b**) and lateral sides, with uniform pore size and distribution (**fig. S1A**). These nanopores are further filled and encapsulated by polydopamine (PDA) nanoparticles on both the front (**Extended Data Fig. 1b**) and lateral (**fig. S1B**) sides of the acid-treated *C. granii*. As illustrated in **fig. S1C**, the *C. granii* shell (*ca.* 1 μm thick) is composed of distinct inner, middle and outer layers. The nanopores distributed on the outer and inner surfaces are bridged by a middle layer (about 1 μm^3) consists of chamber-like compartments. The middle layer (**fig. S1C**) and outer layer (**Extended Data Fig. 1b**) of the DMCG are densely packed with PDA nanoparticles. Such porous-hollow hierarchical micro-nanostructure not only indicates large storage capacity for secure drug encapsulation, but also on-demand switch on-off channels for drug release.

Section S2. Drug loading efficiency

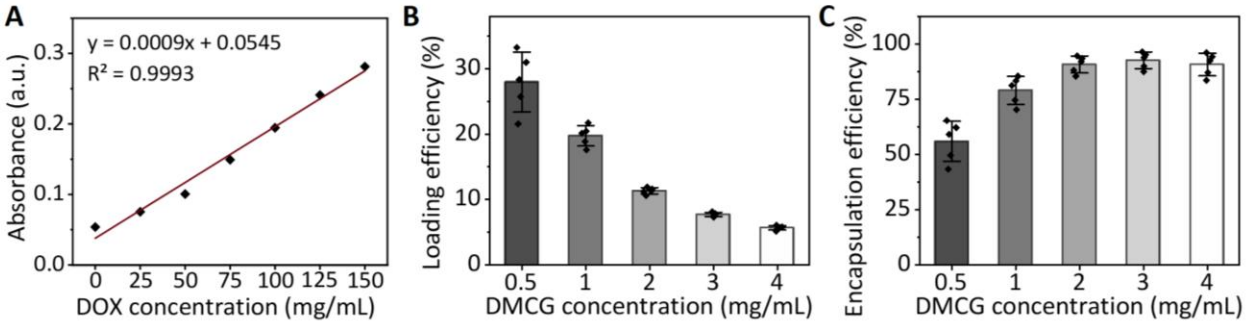

fig. S2. Characterization of drug loading efficiency for DMCG. (A) Standard absorbance curve of DOX versus solution concentration. **(B)** Loading efficiency of DOX in DMCG versus concentration. **(C)** Encapsulation efficiency of DOX in DMCG versus concentration. Data are presented as mean ± s.d. from n = 5 independent samples.

Curve fitting was performed for the absorbance of DOX standard solutions at 480 nm, yielding the standard curve equation: y = 0.0009x + 0.0545, where the absorbance is denoted y and DOX concentration x. After drug loading, the DOX-loaded DMCGs were magnetically separated, with the volume and concentration of the remaining DOX solution measured to calculate the total mass of DOX successfully loaded in DMCG M_d. The drug loading and encapsulation efficiency can be calculated as follows:

$$\text{loading efficiency} = \frac{(M_d - M_s)}{M_C} \times 100\% \quad (1)$$

$$\text{encapsulation efficiency} = \frac{(M_d - M_s)}{M_d} \times 100\% \quad (2)$$

where M_s is the mass of DOX in the supernatant (calculated through the standard absorbance curve) and M_C is the total mass of DMCG in the original solution before drug loading.

Section S3. Setup of coil-based magnetic control

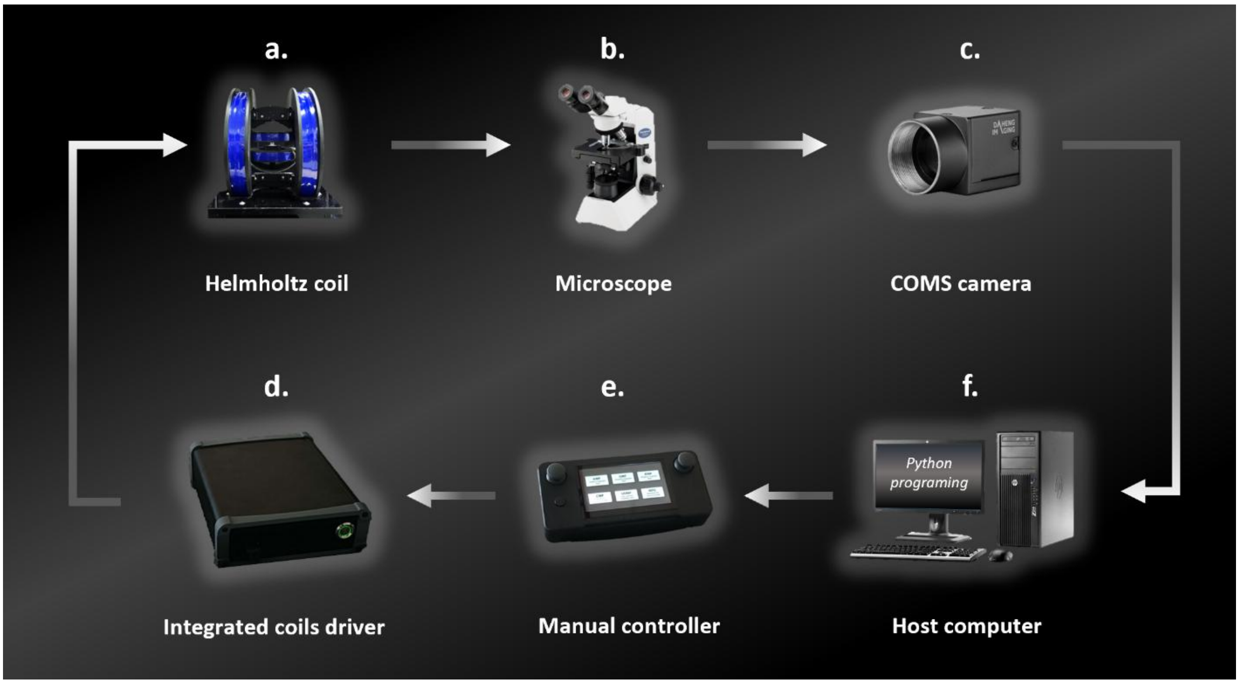

fig. S3. Coil-based magnetic control devices.

The main components of the magnetic control setup include: (**a**) three-axis Helmholtz coils; (**b**) microscope; (**c**) complementary metal-oxide-semiconductor (COMS) camera; (**d**) integrated coil driver; (**e**) manual controller; (**f**) host computer. During operation, a CMOS camera connected to the microscope captures footages of the sample placed within the workspace of the Helmholtz coils. The videos are then transmitted via USB 3.0 to a host computer, where they are displayed in real-time using a Python-based graphical interface. The desired magnetic field driving mode can be selected through a custom-developed manual controller, which is then transmitted to the integrated coil driver to generate the corresponding magnetic field time series for controlling the motion of the samples under observation.

Section S4. Programmable rotating magnetic field

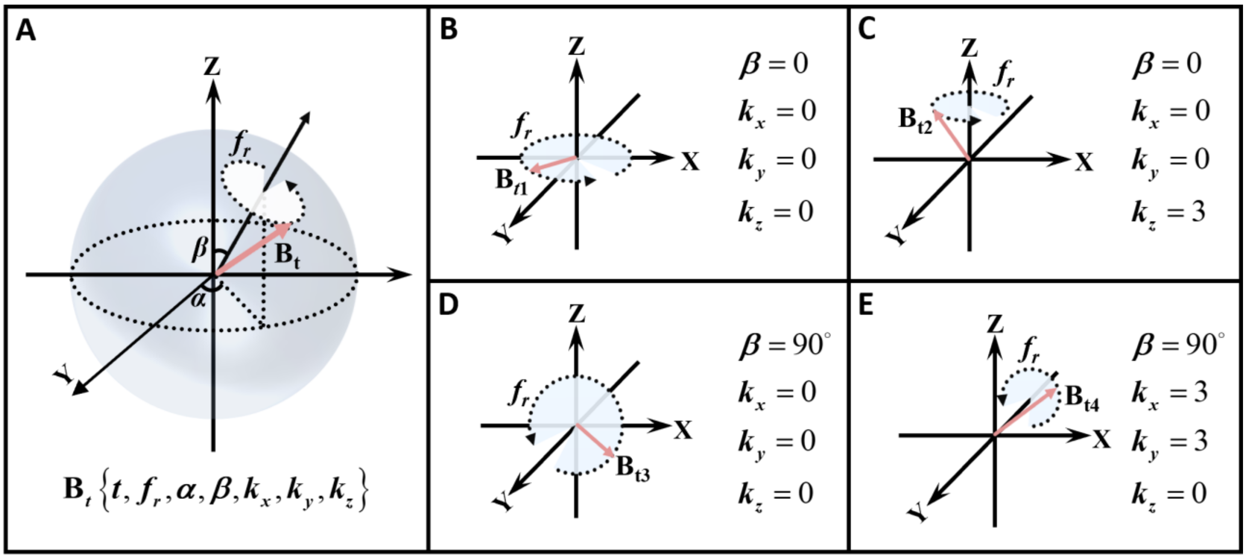

fig. S4. Schematic illustration of the programmable rotating magnetic field. (A) Spherical coordinate system for describing the three-dimensional rotating magnetic field $\mathbf{B_t}$. Four typical magnetic field modes for the distinctive motion regimes of DMCG can be achieved through different combination of control parameters: **(B)** $\mathbf{B_{t1}}$ for rotating, **(C)** $\mathbf{B_{t2}}$ for spinning, **(D)** $\mathbf{B_{t3}}$ for rolling and **(E)** $\mathbf{B_{t4}}$ for tumbling.

The three-dimensional rotating magnetic field is programmed through controlling the parameters f_r, α, β, k_x, k_y, k_z over time t, which represent rotation frequency, yaw angle, pitch angle and spatial parameters for X-, Y- and Z-axis. The governing equation of $\mathbf{B_t}$ is as follows:

$$B_t = [B_x, B_y, B_z]^T = B \begin{bmatrix} \cos\beta \cos\alpha \cos(2\pi f_r t) + \sin\alpha \sin(2\pi f_r t) + k_x \cos\alpha \\ -\cos\beta \sin\alpha \cos(2\pi f_r t) + \cos\alpha \sin(2\pi f_r t) + k_y \sin\alpha \\ \sin\beta \cos(2\pi f_r t) + k_z \end{bmatrix} \tag{3}$$

where $\boldsymbol{B_x}$, $\boldsymbol{B_y}$ and $\boldsymbol{B_z}$ represent the magnetic field component in the x-, y-, and z-directions, respectively. B stands for the magnetic flux density, set as 10 mT by tuning the applied voltage based on coil parameters. The magnetic modes corresponding to the four typical motion regimes.

B_{t1} - rotating, **B_{t2}** - spinning, **B_{t3}** - rolling and **B_{t4}** - tumbling are programmatically tuned via:

$$B_{t1} = B_t(\alpha = \beta = 0, k_x = k_y = k_z = 0) = B \begin{bmatrix} cos(2\pi f_r t) \\ sin(2\pi f_r t) \\ 0 \end{bmatrix} \quad (4)$$

$$B_{t2} = B_t(\alpha = \beta = 0,\ k_x = k_y = 0, k_z = 3) = B \begin{bmatrix} cos(2\pi f_r t) \\ sin(2\pi f_r t) \\ 3 \end{bmatrix} \quad (5)$$

$$B_{t3} = B_t(\beta = 90°,\ k_x = k_y = k_z = 0) = B \begin{bmatrix} sin\,\alpha\, sin(2\pi f_r t) \\ cos\,\alpha\, sin(2\pi f_r t) \\ cos(2\pi f_r t) \end{bmatrix} \quad (6)$$

$$B_{t4} = B_t(\beta = 90°,\ k_x = k_y = 3, k_z = 0) = B \begin{bmatrix} sin(2\pi f_r t) + 3\,cos\,\alpha \\ sin(2\pi f_r t) + 3\,sin\,\alpha \\ cos(2\pi f_r t) \end{bmatrix} \quad (7)$$

Under the magnetic modes **B_{t3}** and **B_{t4}**, DMCG can undergo 2D locomotion motion (**Fig. 2d**) and move from a current position (x_c, y_c) to the target position (x_t, y_t), either along a programmed path or manually controlled using the controller via the following control strategy:

$$\begin{cases} v(f_r) \\ \alpha = arctan\left(\frac{y_t - y_c}{x_t - x_c}\right) \end{cases} \quad (8)$$

Here, $v(f_r)$ can be adjusted through a custom Python program to automatically estimate the resulting translational speed. The locomotion direction is controlled by modulating the yaw angle α of the magnetic field. The locomotion velocity can be adjusted by fine-tuning the value of f_r.

Section S5. DMCG motion in biological fluids

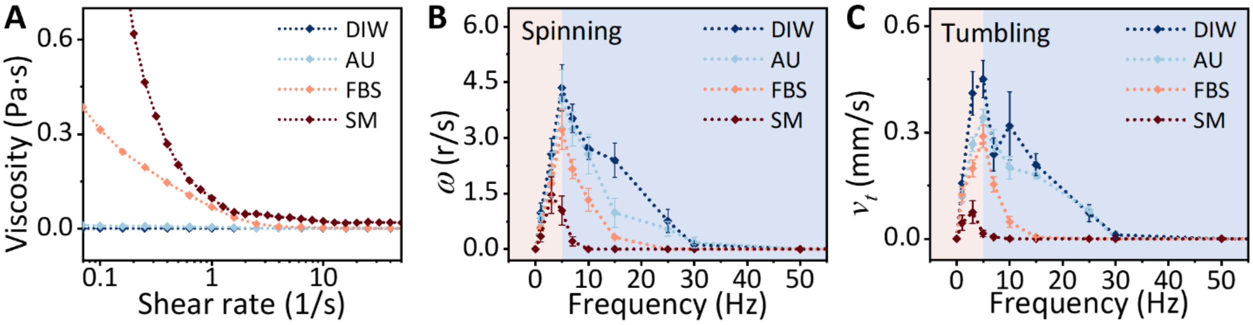

fig. S5. Motion of DMCG in biological fluids of various viscosities. (A) Rheological measurements for deionized water (DIW), fetal bovine serum (FBS), artificial urine (AU) and simulated mucus (SM). DMCG's angular and/or translational velocity versus RMF frequency under the (**B**) spinning and **(C)** tumbling motion regimes in various biological liquids. Data represent mean ± s.d. from n = 5 independent samples.

The flow curves here (**fig. S5A**) for the three biofluidic media characterize their viscosity versus deionized water as $\eta_{\mathrm{sm}} > \eta_{FBS} > \eta_{AU} > \eta_{DIW}$ at varying shear rates. Similar behavior can be observed for the locomotion of DMCG in all tested media, whose angular and/or translational velocities present non-monotonic trends versus the rotation frequency of the magnetic field under spinning (**fig. S5B**) and tumbling regimes (**fig. S5C**) in different biofluidic media. The step-out frequency is approximately 5 Hz for DIW, FBS and AU, but decreases to 3 Hz for SM, in line with the results shown in the main text (**Fig. 2b, c**). In DIW, DMCG can achieve a maximum angular velocity of 4.83 r/s in spinning regime and maximum translational velocity of 0.45 mm/s in tumbling regime.

Section S6. Locomotion control system

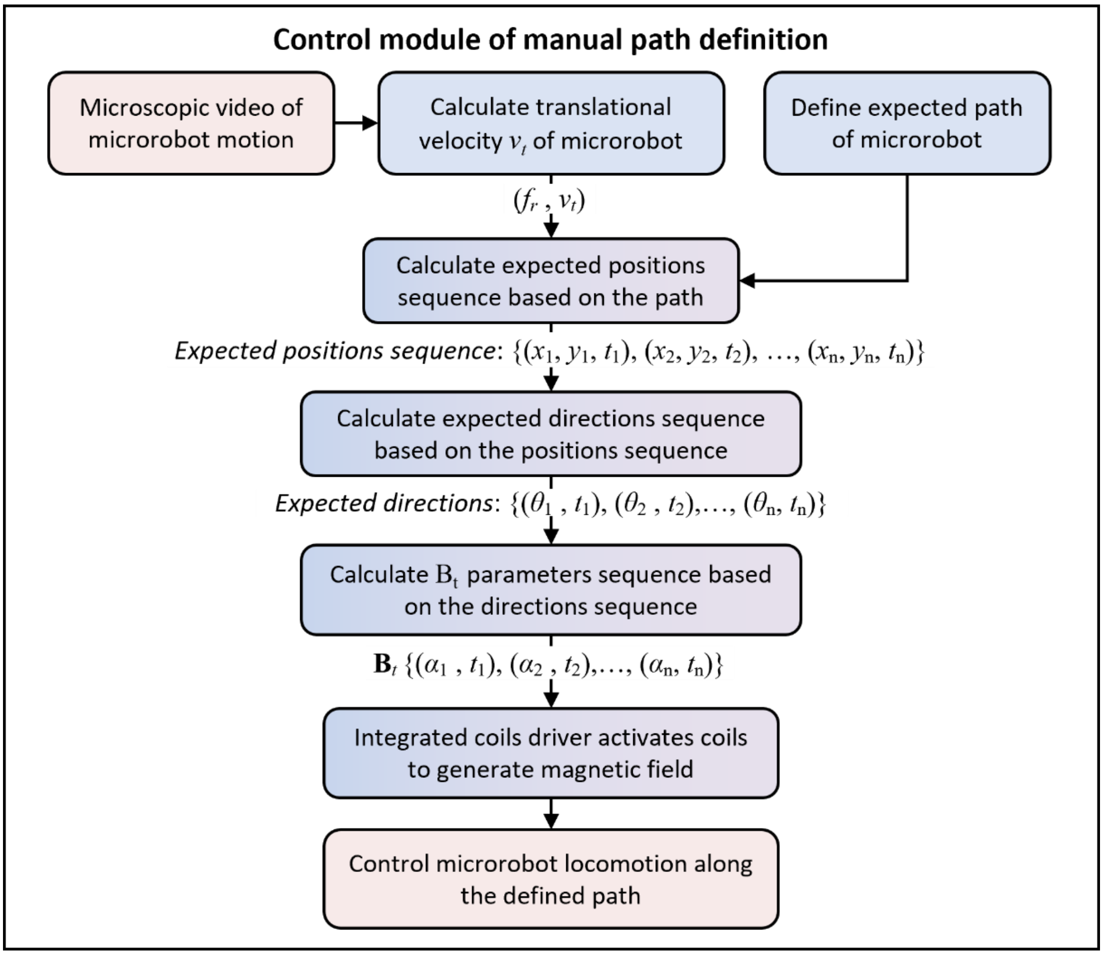

fig. S6. Diagram of the manual path-editing module for pre-defined locomotion control.

In this module, a microscope video capturing the motion of the DMCG is first acquired and processed by a custom Python program, which automatically estimates the translational speed of the DMCG. Following this, the user can manually define the desired motion path through the host computer interface. Once the path is defined, the time sequence of discrete positions along the path, denoted as $\{(x_1, y_1, t_1), (x_2, y_2, t_2), \ldots, (x_n, y_n, t_n)\}$, is computed. This sequence specifies the coordinates and corresponding time instants that the microrobot is expected to reach as it moves along the pre-defined path. Subsequently, the direction sequence $\{(\theta_1, t_1), (\theta_2, t_2), \ldots, (\theta_n, t_n)\}$ is calculated. This sequence determines the orientation of the microrobot at each position, thereby ensuring precise navigation. From the expected direction sequence, the parameter sequence for the magnetic field, $\{(\alpha_1, t_1), (\alpha_2, t_2), \ldots, (\alpha_n, t_n)\}$ can be derived. The computed parameter sequence is then transmitted to the integrated coil driver system, which activates the electric coils to generate the required magnetic field to guide the microrobot in accord with the pre-defined path and directional sequences. The microrobot movement is controlled and monitored as it follows the manually-edited path. No feedback control is involved in the manual path-editing module.

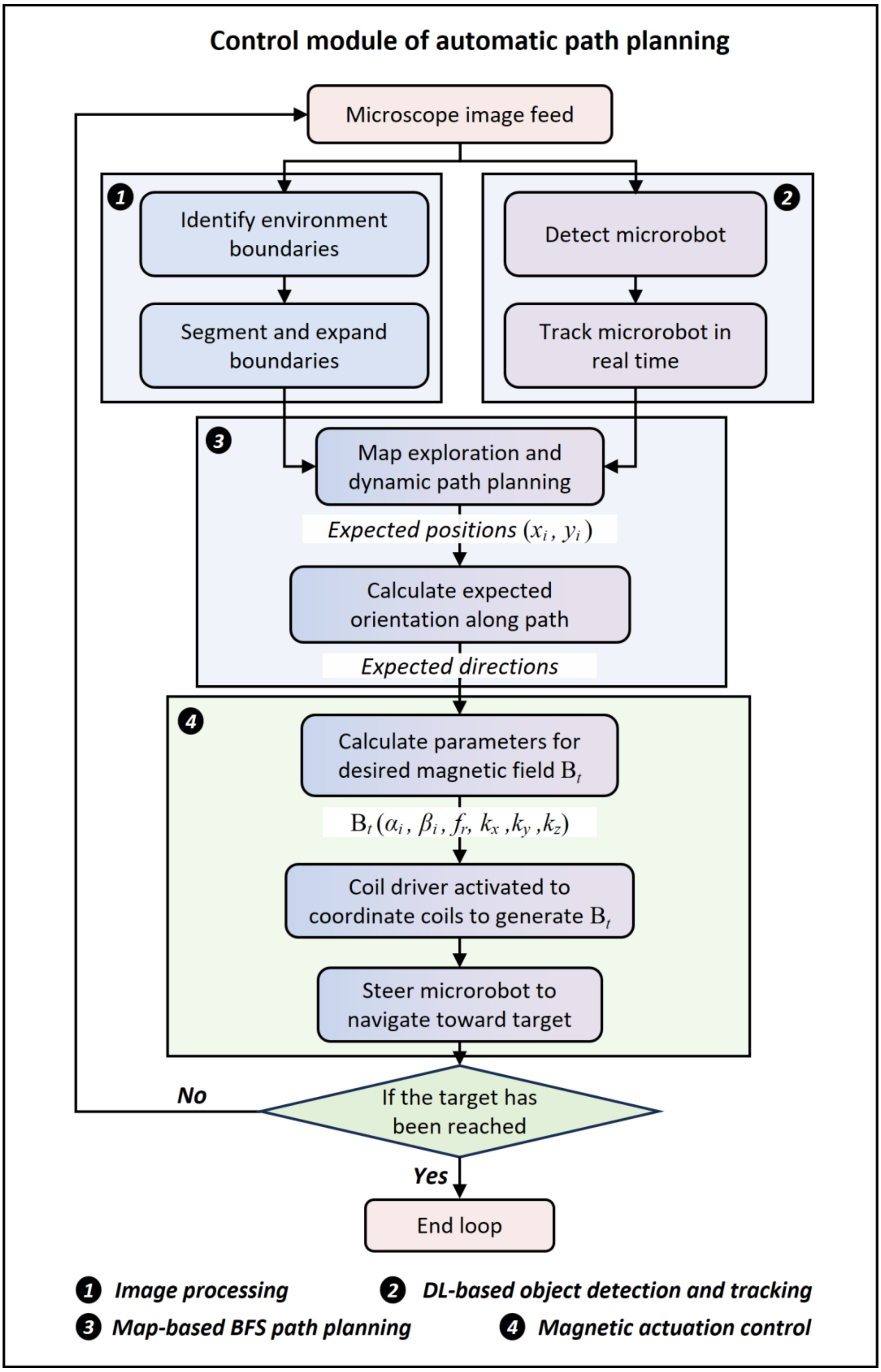

fig. S7. Diagram of automatic path-planning module for autonomous locomotion control.

The autonomous locomotion module begins with feeding live microscope images into the system, which then simultaneously segments the environment boundaries and detects the microrobot position. For boundary segmentation, we employ the Canny edge detection algorithm to delineate where the microrobot can operate. To ensure that the microrobot does not come into contact with the bounding walls, we apply a dilation algorithm to expand the detected boundaries, thereby creating a safety margin around obstacles.

With the expanded boundaries in place, the microrobot is continuously tracked in real-time using a YOLOv5 deep-learning model. The system proceeds to select target locations and plan optimal paths for the microrobot to navigate. Path-planning is then conducted using the Breadth-First Search (BFS) algorithm, which efficiently explores all possible paths to find the shortest and safest route toward the target given the expanded boundaries. This involves calculating the expected positions (x_i, y_i) and directions at each step along the path, based on which the system determines the required magnetic field parameters $\boldsymbol{B_t}$ including the direction angles (α_i, β_i), rotation frequency f_r and spatial parameters $\left(k_x, k_y, k_z\right)$. Once the magnetic field parameters are determined, they are transmitted to the integrated coil driver to activate the coils for producing the required magnetic field to precisely controls the microrobot movement for directing it toward the target. The system continuously monitors the microrobot position and assesses whether the target has been reached. If the target is not yet reached, the system iteratively updates the path and adjusts parameters in real-time, allowing for dynamic navigation with obstacle avoidance until the target is successfully reached.

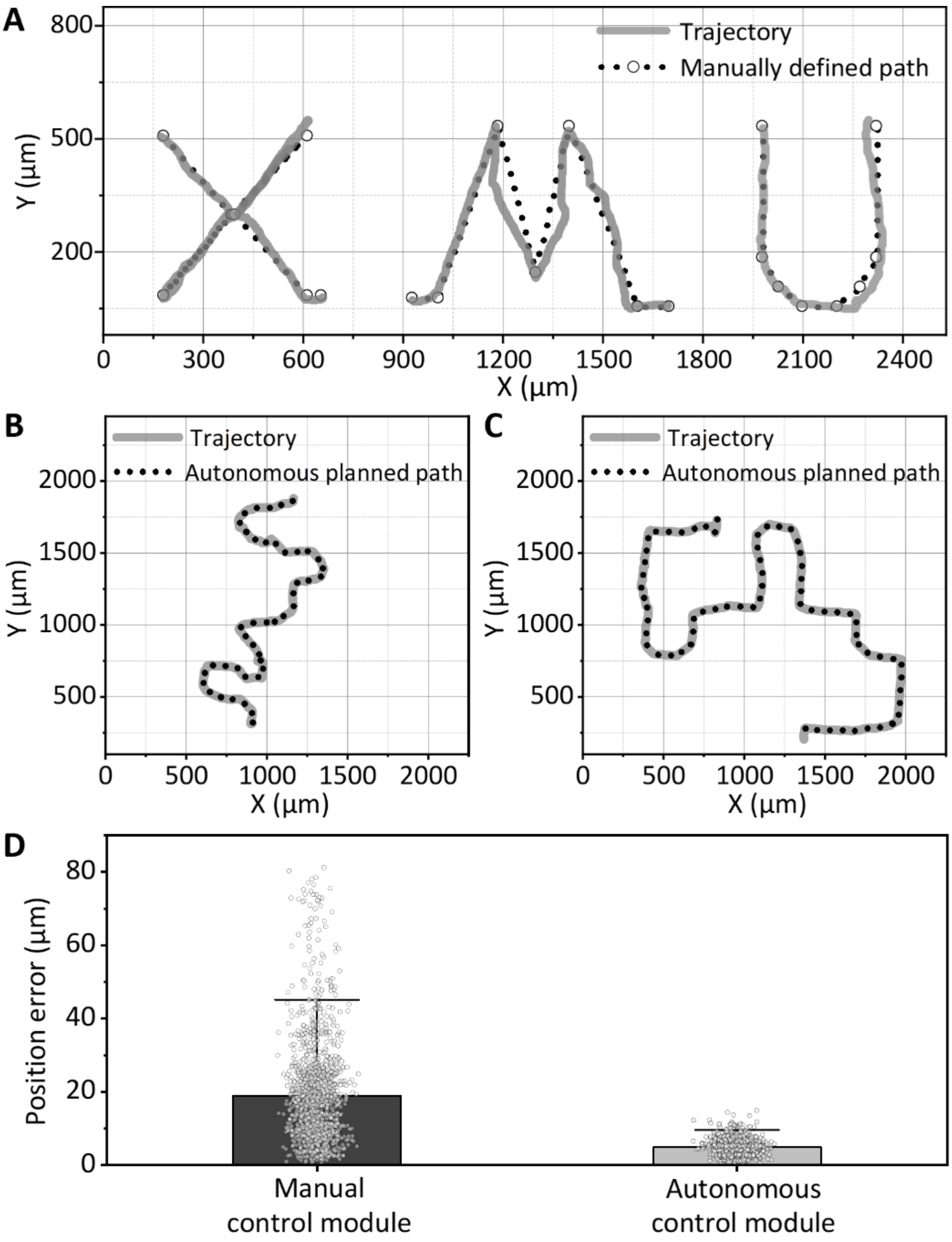

fig. S8. Precision of DMCG navigation with the two control modules. (A) Comparison of the actual trajectory of DMCG with the pre-defined path based on the manual path-editing control module. **(B)** and **(C)** Comparison of the actual trajectory of DMCG with the expected trajectory through real-time planning in the tested mazes (see **Supplementary Video S4**) based on the automatic path-planning control module. **(D)** Statistical analysis of DMCG navigation precision with the two control modes. Note: the dots in (A), (B), (C) are for visual guides only, not the exact expected positions calculated during path planning. Data are presented as mean ± s.d. (n = 1500 time points per group).

Section S7. Mechanical analysis of DMCG motion, multibody assembly and swarm control

DMCG in a fluidic environment experiences several forces and torques that determine its motion.

(1) Magnetic torque T_m:

In the presence of a rotating magnetic field B_t, the magnetic torque T_m driving the rotational motion of DMCG is described by

$$T_m = sin(\theta)\nabla M \times B \tag{9}$$

$$B = \mu_0 \mu H_t \tag{10}$$

$$H_t = \frac{B_t}{\mu_0} \tag{11}$$

where B, H_t, μ and μ_0 denote the magnetic flux density, magnetic field strength, relative permeability and vacuum permeability, respectively. θ is the angle between M and B.

(2) Magnetic force F_m: $F_m = \mu_0(M \cdot \nabla)H$ (12)

(3) Viscous drag force F_d:

When DMCG undergoes motion within a fluid in low-Reynolds flow regime, the viscous drag force F_d opposing the motion across the fluid is described as

$$F_d = 6\pi\eta_l R^2 \omega_r \tag{13}$$

where η_l is the fluid dynamic viscosity. R,ω_r represent the DMCG radius and angular velocity, respectively.

(4) Gravity F_g:

The gravity force due to the weight of the DMCG is described as

$$F_g = \rho g V \tag{14}$$

where g is the gravity-induced acceleration. ρ , V are the DMCG volume and density, respectively.

(5) Buoyancy F_b:

The buoyancy force of DMCG reduces the normal force N of the substrate and is described as

$$F_b = \rho_l g V \tag{15}$$

where ρ_l represent the liquid density.

(6) The normal force N from the substrate:

In the perpendicular direction, the force balance satisfies

$$N = F_g - F_b = g\,V(\rho - \rho_l) \tag{16}$$

(7) Frictional force F_f:

The frictional force resisting in-place or translational DMCG motion on the substrate is written as

$$F_f = f_s\text{N}=f_s g\,V(\rho - \rho_l) \tag{17}$$

where f_s is the friction coefficient between the moving DMCG and the substrate.

(8) Torque balance

Beyond force balance, there is also torque balance for the rotational equilibrium of DMCG. The resistive torque T_d generated by viscous drag force F_d could be estimated by

$$T_d \approx 6\pi\eta_l R^2 \omega_r \quad (18)$$

For rotation at equilibrium, the magnetic torque must balance the total resistive torque:

$$T_m = T_d \quad (19)$$

As the RMF rotation frequency increases, the required angular velocity ω_r of DMCG to sync with the rotating magnetic field also increases, causing increasing T_d proportional to ω_r. If $T_m < T_d$, DMCG will slip with step-out behavior, failing to maintain synchronized motion with the RMF.

(9) Multi-body assembly

The magnetic dipole force between two DMCGs (i, j) at a distance r can be calculated by

$$F_{ij} = m_i \cdot \nabla H_{ij} = \frac{3}{4\pi\mu_0 r_{ij}^4}[(m_i \cdot m_j)r_{ij} + (m_i \cdot r_{ij})m_j + (m_i \cdot r_{ij})m_i - 5(m_i \cdot r_{ij})] \quad (20)$$

where m_i is the magnetic dipole moment of the i-th DMCG, $H_{ij}$the magnetic field imposed by the j-th DMCG on the i-th DMCG, and r_{ij} the normalized direction. The total magnetic force on each DMCG arises from its interactions with other DMCGs in the system. Summation of all interaction forces can therefore estimate the magnetic force acting on the DMCG assembly. The rotational torque can be determined by summing up the individual torques as follows:

$$T_{a,m} = \sum_i (R_i \times \sum_j F_{ij}) \quad (21)$$

where index i refers to a specific DMCG in the assembly, and index j iterates over other DMCGs in the system. R_i denotes the position of the i-th DMCG relative to the assembly center. For DMCG with diameter R, R_i could be derived given the assembly geometric configuration:

$$R_i = \frac{R}{sin(\frac{\pi}{n})} \quad (22)$$

$$T_{a,m} = R_i \sum_i \sum_j F_{ij} \quad (23)$$

where n is the number of assembling units. The viscous drag torque experienced by the DMCG assembly can be further written as

$$T_{a,d} = 6\pi\eta(R + R_i)^3 \omega_r \quad (24)$$

The cubic dependence on R_i infers that as the number of assembling units increases, the viscous drag torque grows significantly. If $T_{a,m} < T_{a,d}$, the DMCG assembly cannot maintain synchronized motion with the RMF and may disassemble into individual rotating bodies or degrade to smaller assembly of fewer units. Optimal design and control of $T_{a,m}$ and ω_r may contribute to stable assembly of designated DMCG configurations.

Section S8. Setup and magnetic control of permanent-magnet system

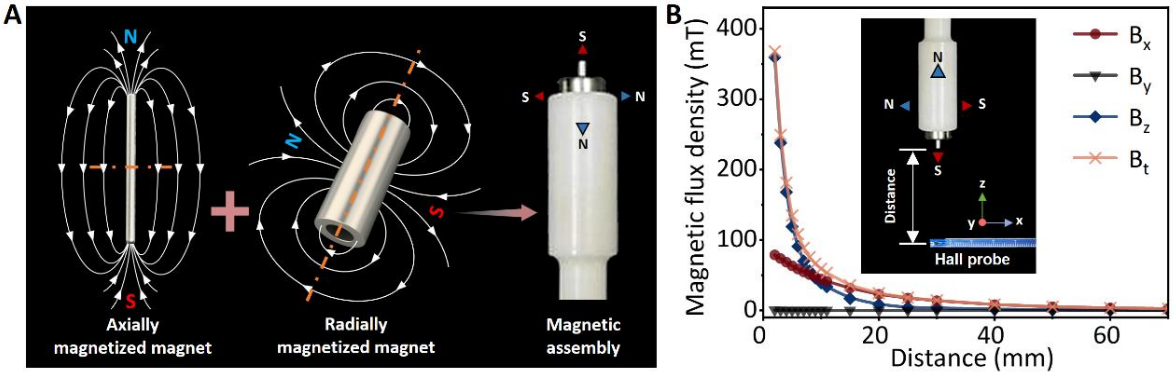

fig. S9. Configuration of the permanent magnet setup and measurement of its magnetic flux density at varying distances. (A) Illustration of the nested permanent magnet setup composed of axially-/radially- magnetized rod/shell magnets. **(B)** Total magnetic flux density ($\mathbf{B_t}$) and directional components ($\mathbf{B_x}$, $\mathbf{B_y}$, $\mathbf{B_z}$) measured at varying distances along the central axis of the magnet.

An axially magnetized rod magnet (length 55 mm, diameter 3 mm, NdFeB) was nested within a radially magnetized shell magnet (length 50 mm, outer diameter 15 mm, inner diameter 8 mm, NdFeB) using a 3D-printed fixture (**fig. S9A**). The goal was to use this combined magnet (*nRSM*) for generating conical-like gradient fields at short distances (conducive to swarm assembly) and quasi-uniform orthogonal fields at large distances (conducive to swarm swirling), given the respective field characteristics of the rod and shell magnets. Magnetic field measurements for nRSM (**fig. S9B**) along its revolution axis showed that the axial component B_z dominants within 0–10 mm but experiences a rapid decay and then vanishing strength beyond 20 mm, whereas the radial component B_x has a gradual decay overall and dominates beyond 20 mm with a profile closely matching that of total field strength $\mathbf{B_t}$.

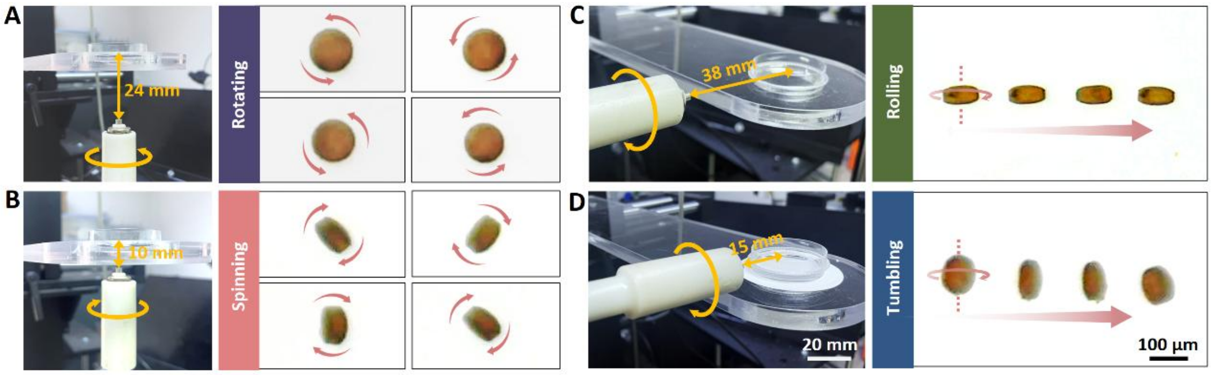

fig. S10. Experimental replication of the four DMCG motion regimes with the permanent magnet system. The left side of each panel shows the relative position, distance d of the magnet to the work plane, and the right side shows the time-lapse DMCG motion under each regime: **(A)** rotating (d = 24 mm); **(B)** spinning (d = 10 mm); **(C)** rolling (d = 38 mm); **and (D)** tumbling (d = 15 mm).

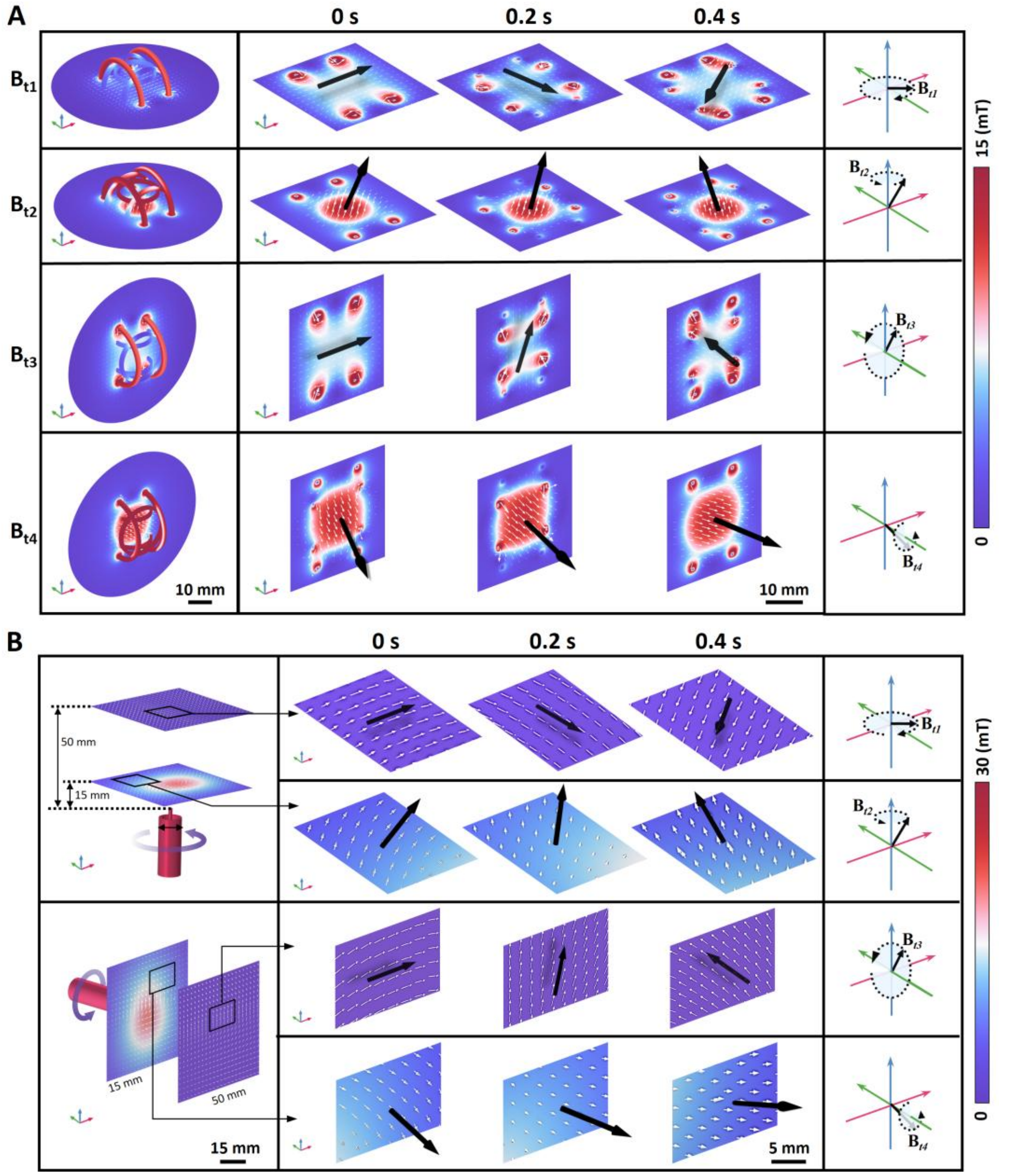

fig. S11. Numerical simulation of the four magnetic field modes (B_{t1}, B_{t2}, B_{t3}, B_{t4}). In each mode, the distribution of magnetic flux density and the direction of magnetic scalar potential are shown for the **(A)** electromagnetic coil system and **(B)** permanent magnet system, respectively.

(1) When the magnet is placed perpendicularly at a fairly large distance from the microrobot plane (e.g., 50 mm), there is minimal horizontal gradient and the magnetic field pattern resembles the $\mathbf{B_{t1}}$ mode of the coil-based system. (2) If the distance is shortened (e.g., 15 mm), the magnetic scalar potential exhibits a conical distribution on the horizontal plane, corresponding to the $\mathbf{B_{t2}}$ mode of the coil-based system. (3) If the magnet is placed horizontally to the microrobot plane instead, its magnetic field structure corresponds to the $\mathbf{B_{t3}}$ or $\mathbf{B_{t4}}$ modes, depending on the operating distance (i.e., 50 mm or 15 mm).

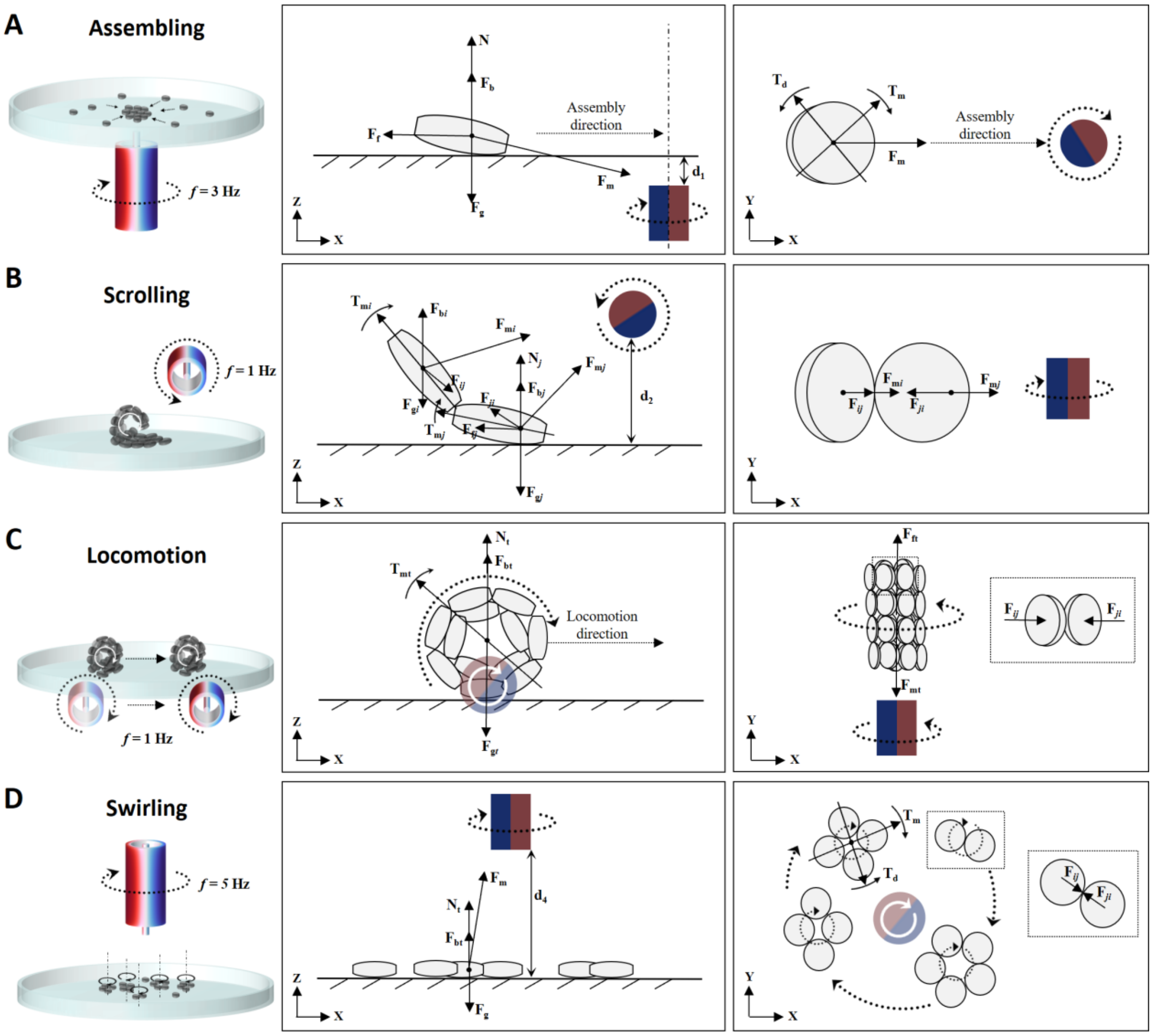

fig. S12. Multimodal magnetic control of a DMCG swarm for reconfigurable distribution. **(A) Assembling** (f = 3 Hz, d_1 = 5 mm, B = 90 mT). By perpendicularly rotating the magnet closely beneath the substrate, a high-gradient magnetic field is generated to rapidly focus dispersed DMCGs with inward motion into a compact vortex-like swarm. **(B) Scrolling** (f = 1 Hz, distance d_2 = 10 mm, B = 70 mT). Once the vortex-like swarm is formed, the magnet is switched to the lateral side of the swarm with lower rotation frequency to gradually scroll the swarm into a wrapped shell. **(C) Locomotion** (f = 1 Hz, distance d_3 = 10 mm, B = 70 mT). Applying a translational displacement on top of the scrolling operation would steer the shell-like swarm into locomotion mode, with synchronized magnetic dipole interactions maintaining the integrity of the swarm during motion. **(D) Swirling** (f = 5 Hz, d_4 = 30 mm, B = 20 mT)**.** Applying a low-strength, low-gradient and high-frequency rotating magnetic field would weaken the magnetic dipole interactions within a vortex-like swarm while enhancing the viscous drag force and resistive torque, leading to disassembly of the compact swarm into multiple swirling units at high speed.

Four operation modes of the swarm (left panel) using a nested rod-shell permanent magnet (nRSM) are illustrated, alongside force analysis with a side view (middle panel) and a top view (right panel). The forces are gravitational force $\mathbf{F_g}$, buoyancy force $\mathbf{F_b}$, frictional force $\mathbf{F_f}$, magnetic gradient force $\mathbf{F_m}$, drag force $\mathbf{F_d}$, surface normal force $\mathbf{N}$, magnetic torque $\mathbf{T_m}$ and drag torque $\mathbf{T_d}$. The magnetic dipole interactions between individual DMCGs contribute to a stable swarm.

Section S9. Characterization of swarming phase change conditions

Parametric experiments on the swarm control for clarifying their operational conditions, with particular emphasis on the phase transition conditions listed below:

(1) Assembling: We first quantified the assembling transition of the microrobot swarm from a dispersed state to aggregated states across a range of rotation frequencies and magnet distances (proxy of field strengths), with the magnet positioned perpendicular to the work surface (**fig. S13,** see operation schematic in **fig. S12**). Integrated grayscale analysis (product of average greyscale value and aggregated area) was performed in predefined target regions and the results were visualized as a heatmap (**fig. S14**). The swarm assembly strength is found inversely correlated with both the frequency and distance. Optimal assembly can be achieved under short magnet distances (5–20 mm) and low rotation frequencies (1–3 Hz). Increasing either parameter leads to a decrease in assembly strength.

(2) Scrolling & Locomotion: Once the DMCGs were aggregated in the assembling mode, the magnet position is adjusted from perpendicular to sidewise with respect to the work surface for scrolling operation (see operation schematic in **fig. S12**), and with additional magnet displacement, locomotion mode. The strength of scrolling swarm versus the magnet distance and rotation frequency presents similar trend to that of the assembling operation (**fig. S15**). As the phase diagram shows (**fig. S16**), the strongest scrolling effect with a stable single coiled-body undergoing rolling motion occurs at frequency <2 Hz, distance < 30 mm. By increasing either frequency or distance, a morphological transition occurs: first to multiple coiled bodies in coordinated motion, and subsequently at higher frequencies (>4 Hz) and/or larger distances (>30 mm), to discrete rolling aggregates with no stable coiled structures.

(3) Swirling: The swirling mode can be achieved by rotating (with or without breaking up) the compact swarm aggregated through either assembling or scrolling modes. In the swirling mode, the magnet is positioned sidewise with respect to the work surface (see operation schematic in **fig. S12**). The number of stable swirling aggregates varies against the magnet distance and ration frequency (**fig. S17**), and the operation conditions are summarized into a phase diagram (**fig. S18**). At very low frequencies (0–1 Hz), a unified swarm is favored. Conversely, high frequencies (>5 Hz) in tandem with moderate magnet distances (40–70 mm) induce a proliferation of swarms (Beyond 20). An intermediate state with moderate numbers of swarms (2 to 20) exists and the transition behaves in a non-monotonic manner with respect to the operating distance. For magnet distances < 70 mm, the number of swirling units increases with both parameters, owing to stronger hydrodynamic breakup under increasing rotational flows at higher frequencies and weaker magnetic dipole attraction under decreasing field strengths at larger distances. If the distances exceed 70 mm, the number decreases instead, presumably due to asynchronized motion under weak field strength and consequent reduction in the fluidic forces required for breaking up the swarm.

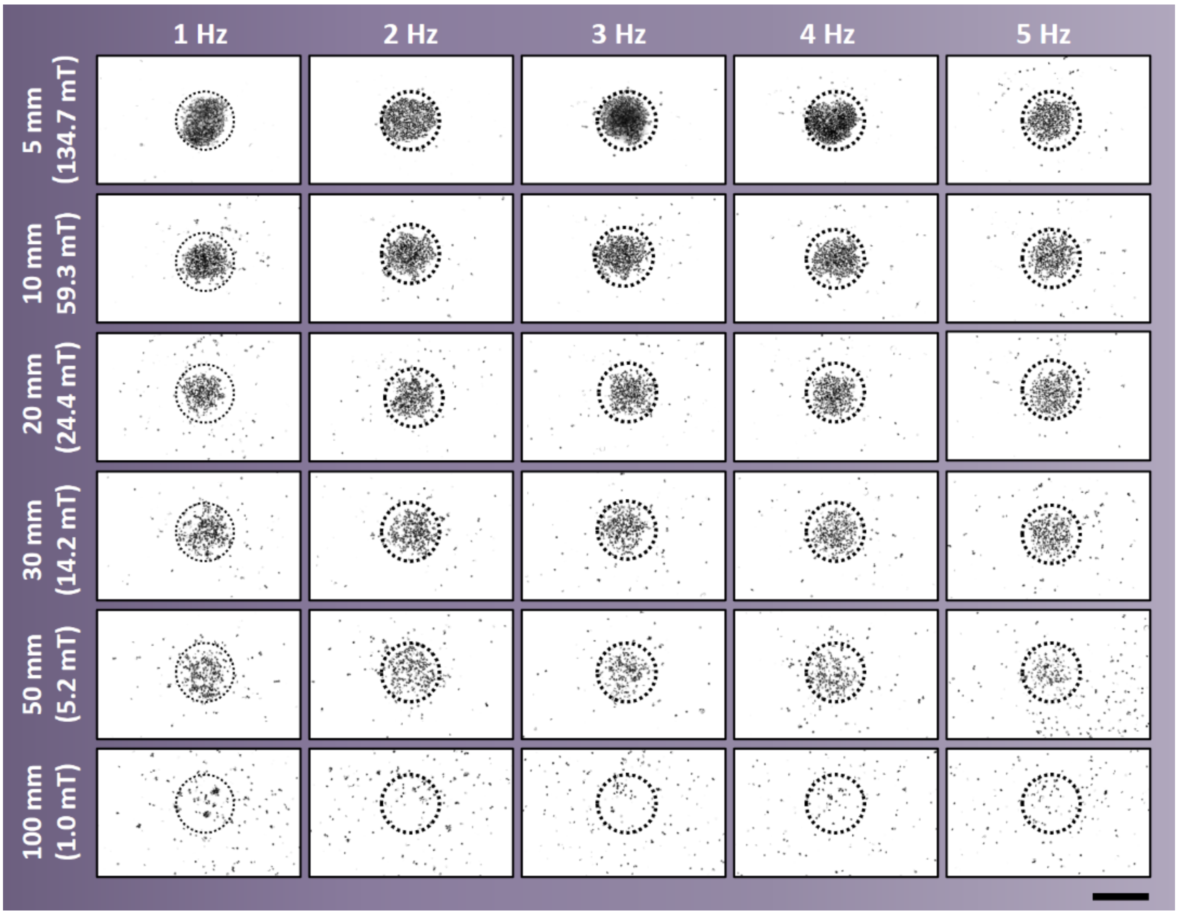

fig. S13. Representative images of the parametric study on the DMCG swarm assembling patterns versus operating magnet distance (5–100 mm; 134.7–1.0 mT) and rotation frequency (1–5 Hz). Dotted circles indicate the target aggregation region. Scale bar, 3 mm.

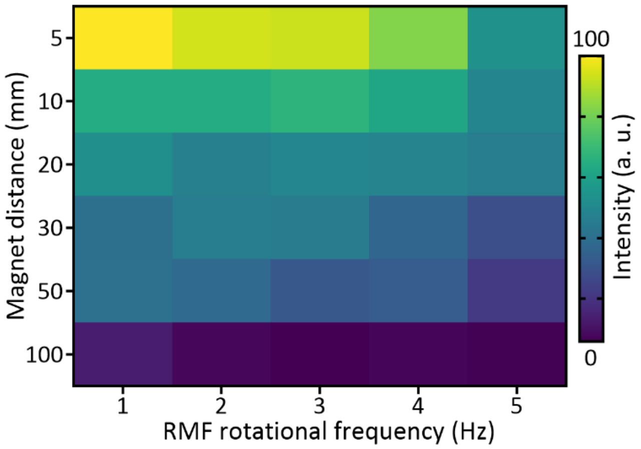

fig. S14. Heatmap of the DMCG swarm assembling strength versus operating magnet distance (5–100 mm) and rotation frequency (1–5 Hz). The color scale (0-100) represents the relative assembly intensity in arbitrary units (a.u.).

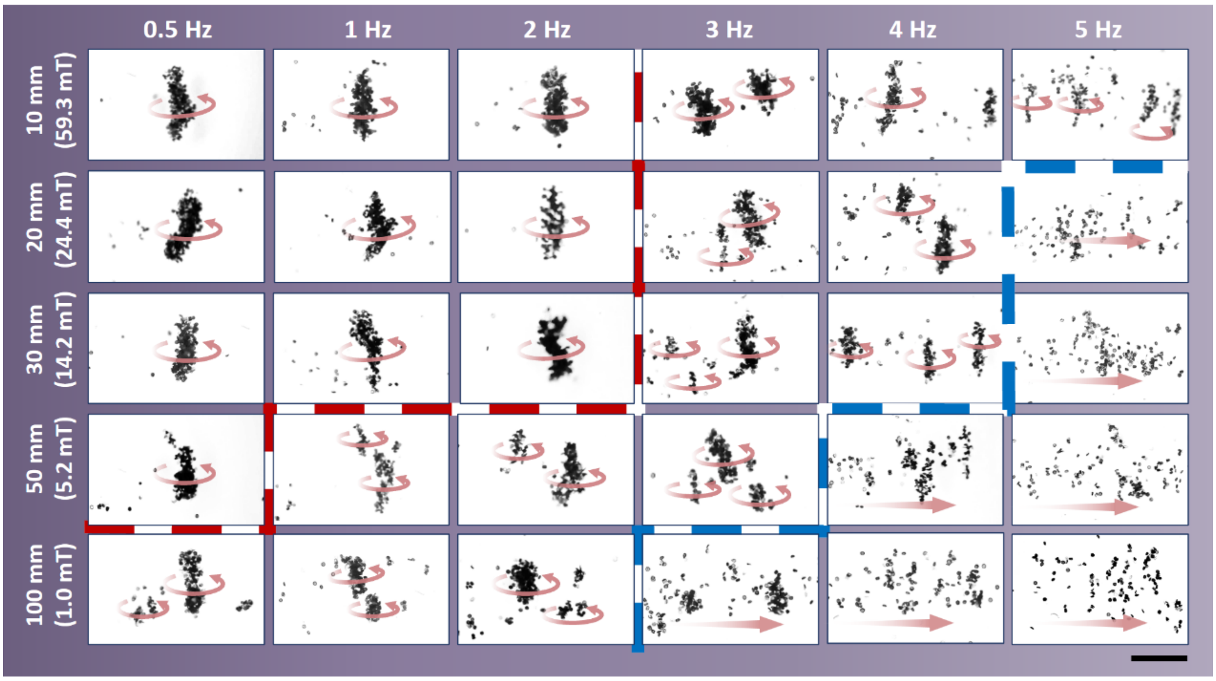

fig. S15. Representative images of the parametric study on the DMCG swarm scrolling-locomotion patterns versus operating magnet distance (10–100 mm; 59.3–1.0 mT) and rotation frequency (0.5–5 Hz). Red and blue lines delineate three transitional states: (i) single curled-swarm body, (ii) multiple curled-swarm bodies, and (iii) discrete DMCG clusters. Scale bar, 1.5 mm.

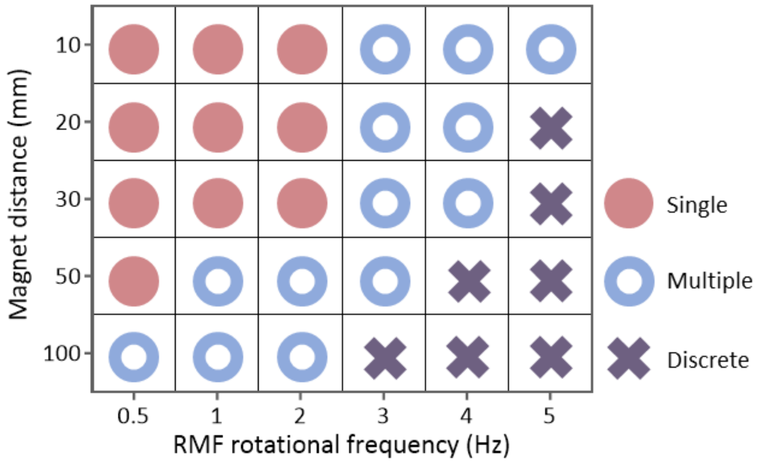

fig. S16. Transition phase diagram of the DMCG scrolling-locomotion states versus operating magnet distances (10–100 mm) and rotation frequency (0.5–5 Hz). The red filled circles, blue hollow circles, and gray crosses denote state (i), state (ii), and state (iii) as described in fig. S15, respectively.

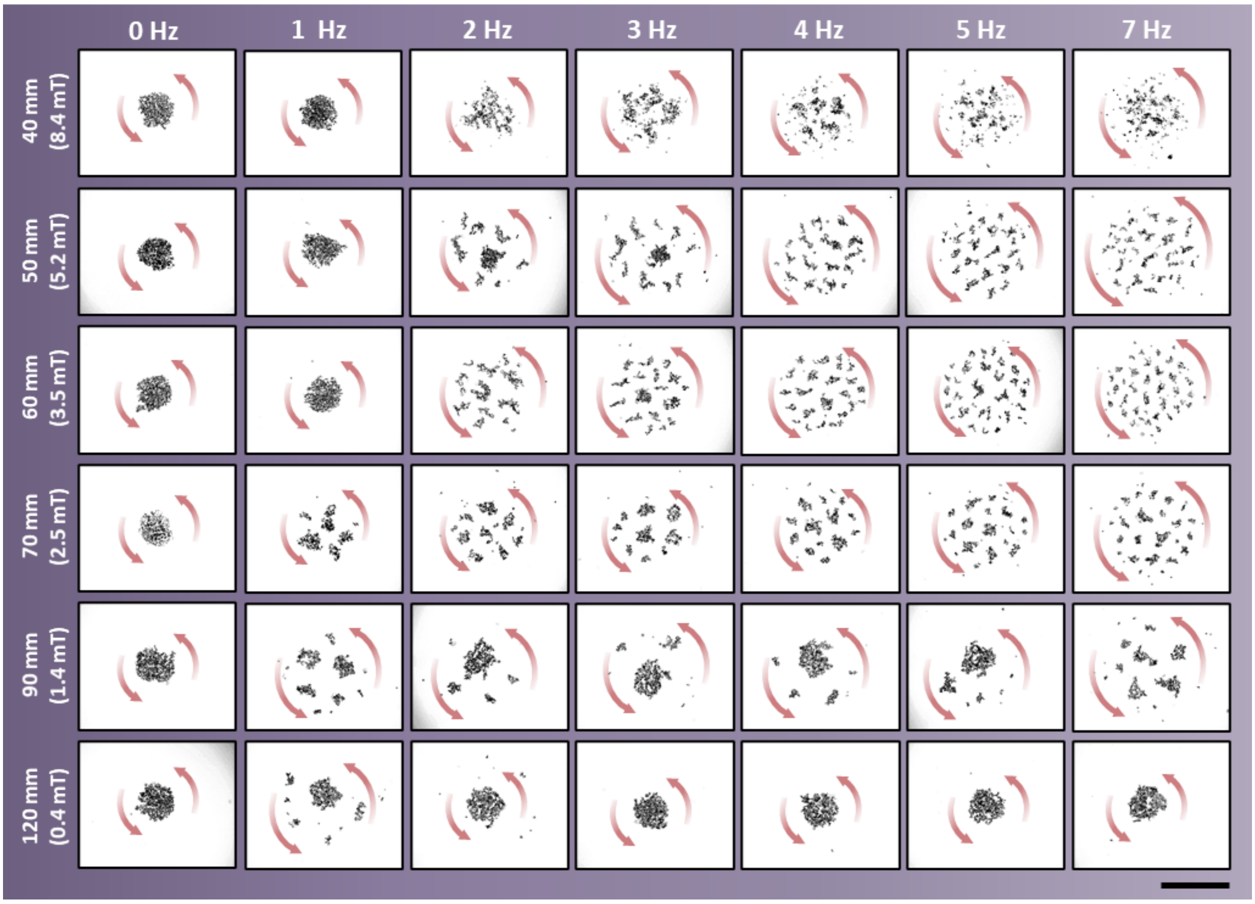

fig. S17. Representative images of the parametric study on the DMCG swarm swirling patterns versus operating magnet distances (40–120 mm; 8.4–0.4 mT) and rotation frequency (0–7 Hz). Scale bar, 3 mm.

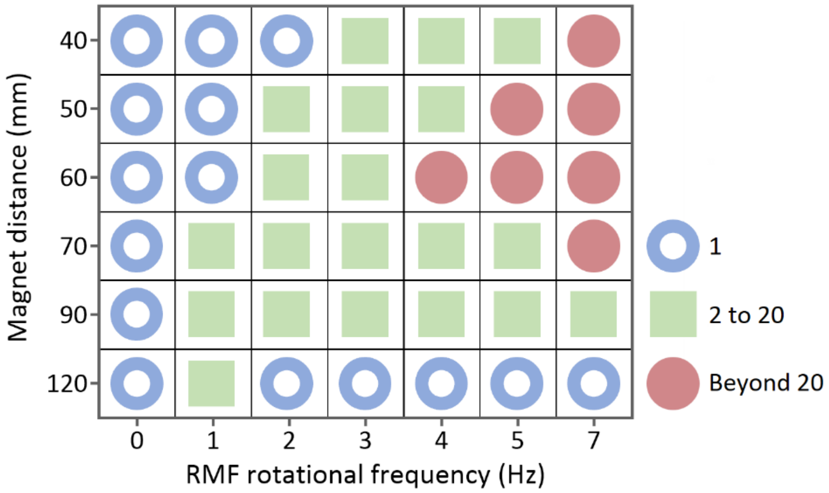

fig. S18. Transition phase diagram of the DMCG swirling states versus operating magnet distance (40–120 mm) and rotation frequency (0–7 Hz).

Section S10. DMCG locomotion in complex environments

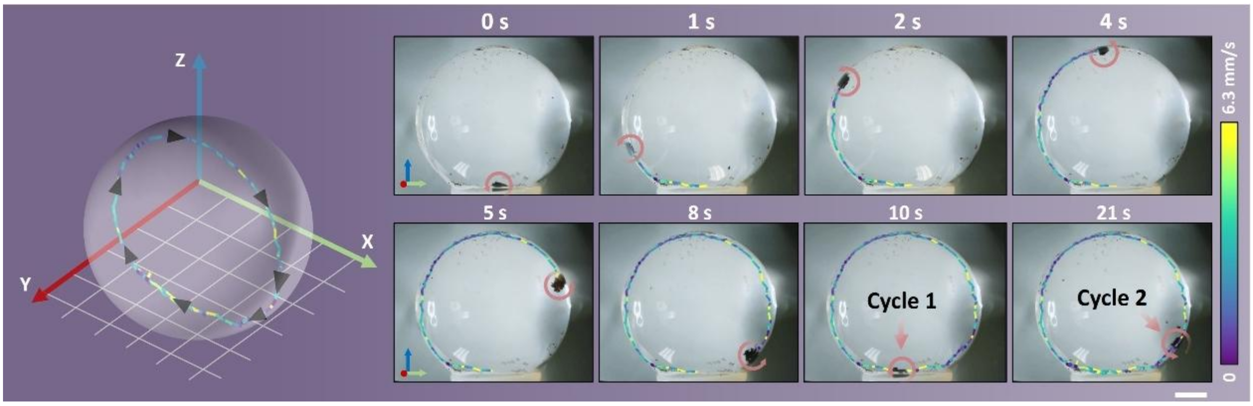

fig. S19. Gravity-resisting locomotion of DMCG swarms. The swarm moves inside a 3D hollow quartz sphere simulating the bladder, where time-lapse sequence demonstrates stable climbing and reproducible locomotion cycles (about 10 s each). Scale bar, 4 mm. Video footage available in **Video S6**.

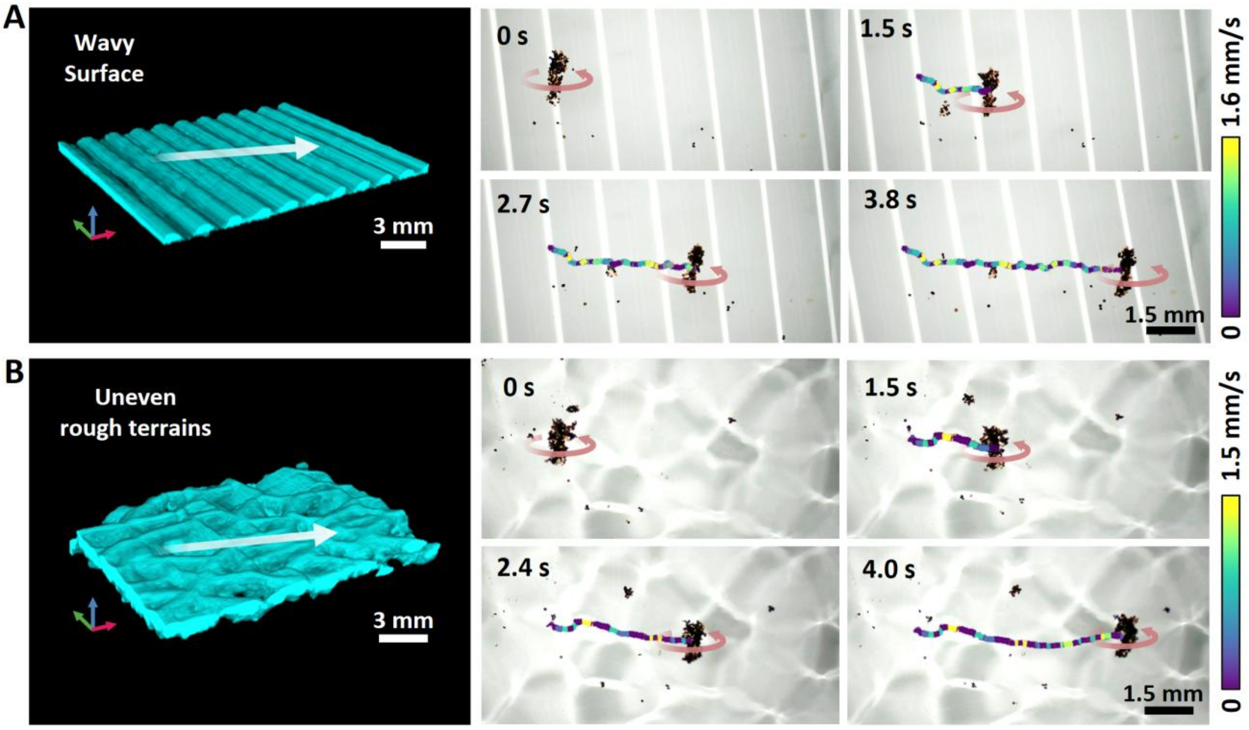

fig. S20. Robust locomotion of DMCG swarms on complex 3D surfaces. (A) Grooved surface. **(B)** Rough terrains. **(left)** 3D-scanned microscopic topography. **(right)** Swarm trajectory and speed. Video footage available in **Video S6**.

Section S11. Particle image velocimetry of DMCG motion

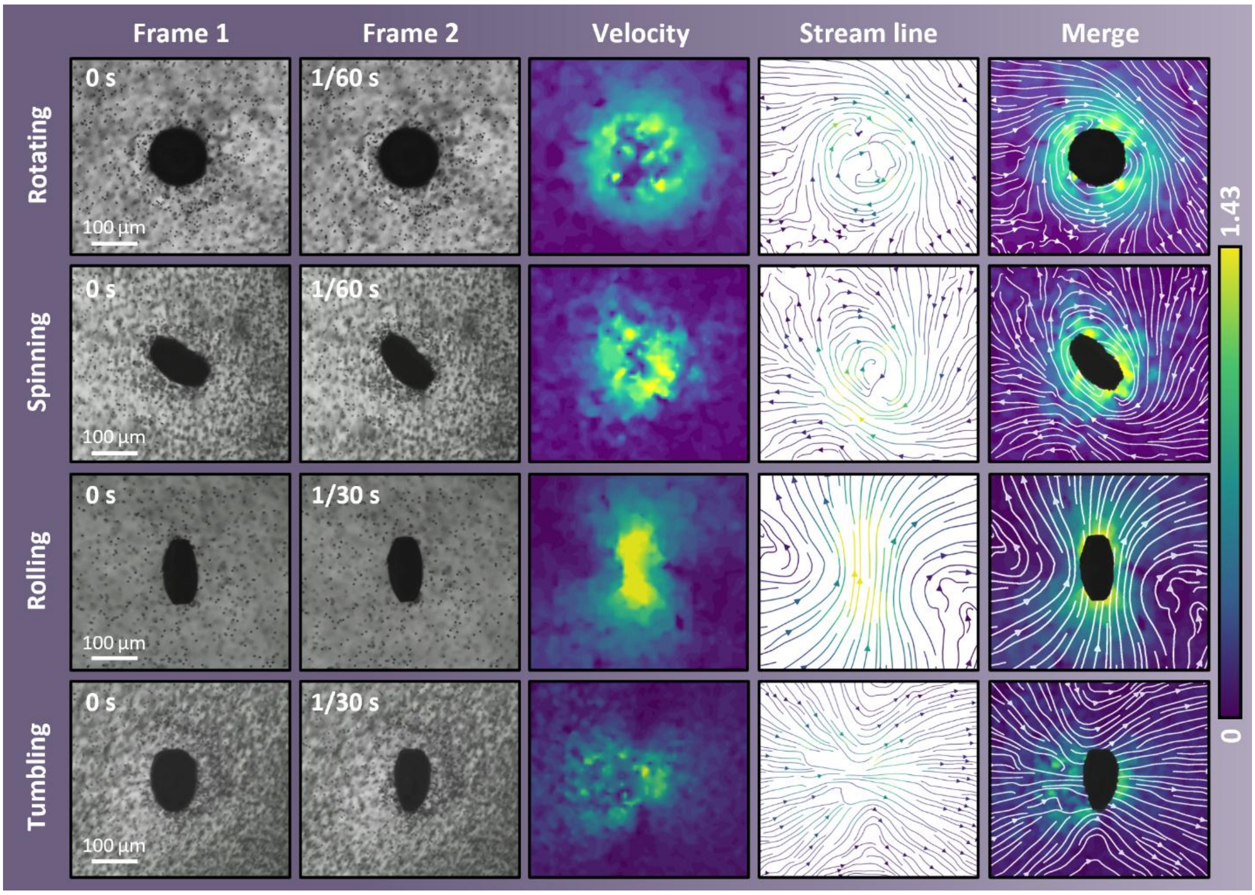

fig. S21. Particle image velocimetry (PIV) data of DMCG motion experiments. From left to right, top to bottom: experimental image, velocity quantification and streamline contour of DMCGs under four distinct motion regimes, namely rotating, spinning, rotating and tumbling (legend unit: mm/s).

PIV experiments were conducted to measure the flow pattern and instantaneous fluid velocities during the motion of DMCGs, following these steps: **1**) 1 µm-sized SiO_2 particles, serving as tracer particles, were added to deionized water (DIW) at a concentration of 0.1 mg/mL. **2**) DMCG was introduced into this tracer fluid. **3**) Apply the magnetic control setup (**fig. S3**) to generate rotating magnetic fields for driving the motion of DMCG under four typical control modes (**fig. S4**, RMF rotation frequency 5 Hz). Videos capturing the movement of the DMCG and the tracer particles were then recorded. Consecutive frames from these videos were selected and imported into a PIV processing program for analysis. The motion of the particles between these frames was used to calculate the velocity field of the fluid.

Section S12. Simulation model and parameters

Table S1. The geometry construction and model configuration in COMSOL for different simulation scenarios.

Scenario	Geometry and domain	Physical model
motion-induced fluid flow (single DMCG)	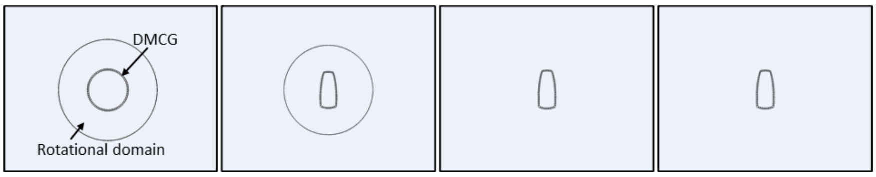	laminar flow, porous media
motion-induced fluid flow (DMCG assembly)	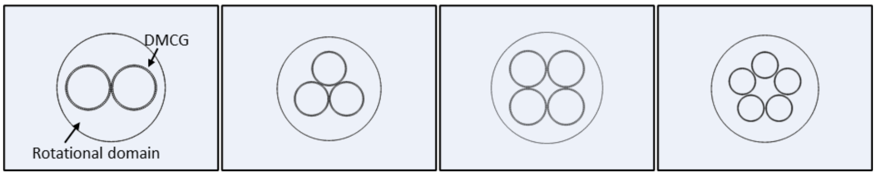	
convection-enhanced drug release	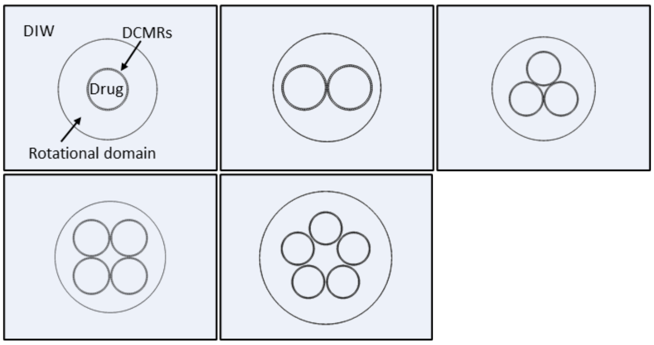	laminar flow, convection-diffusion, porous media
convection-enhanced barrier penetration	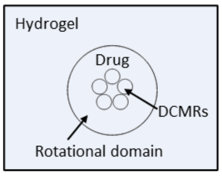	
magnetic field around artificial bladder	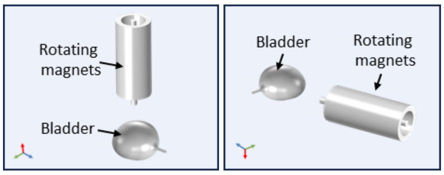	magnetic field

Table S2. Main simulation parameters.

fluid viscosity η_l	fluid density ρ_l	nanopore porosity ε_p	PDA size d_{poly}	hydrogel permeability κ^{-1}	diffusion coefficient D_d	magnetic flux density B	magnetic suscepti-bility χ	rotation frequency f_r
0.001 (Pa s)	1000 (kg/m^3)	0.01	10 (nm)	10^{-12} (m^2)	DIW, 1×10^{-9} (m^2/s); Hydrogel, 1×10^{-11} (m^2/s)	10 (mT)	0.31	0-5 (Hz)

Section S13. Governing equations of the simulation

(1) Laminar flow model

The fluid flow dynamics are governed by the steady-state incompressible Navier-Stokes equations. The momentum conservation is expressed as

$$\rho\frac{\partial t}{\partial u} + \rho(u \cdot \nabla)u = \nabla \cdot (-pI + K) + F \tag{25}$$

where ρ_l is the fluid density, u is the velocity field, p is the pressure, I is the identity matrix, K represents the viscous stress tensor, and F is the external body force per unit volume. The continuity equation for incompressible flow is given by

$$\rho_l \nabla \cdot u = 0 \tag{26}$$

The viscous stress tensor K is defined as

$$K = \eta_l(\nabla\vec{u}_l + (\nabla\vec{u}_l)^T) \tag{27}$$

where η_l is the dynamic viscosity of the liquid.

(2) Flow through porous media

To accurately represent the fluid flow across the porous media formed by nanopores filled with polydopamine particles, a modified version of the Navier-Stokes equations is used:

$$\frac{1}{\varepsilon_p}\rho\frac{\partial \boldsymbol{u}}{\partial t} + \frac{1}{\varepsilon_p}\rho(\boldsymbol{u} \cdot \nabla)\boldsymbol{u}\frac{1}{\varepsilon_p} = \nabla \cdot (-p\boldsymbol{I} + \boldsymbol{K}) - \left(\mu_l \kappa^{-1} + \beta\rho|\boldsymbol{u}| + \frac{Q_m}{\varepsilon_p^2}\right)\boldsymbol{u} + \boldsymbol{F} \tag{28}$$

where ε_p represents the medium porosity, with its reciprocal accounting for the reduced volume available for fluid flow due to the presence of solid particles in the nanopore. β is a coefficient representing the form drag in the porous medium. For the permeability κ^{-1} of the porous media formed by the granular polymers, the Kozeny-Carman equation is used to provide an empirical estimate based on overall porosity and particle size:

$$\kappa = \frac{d_p^2}{180}\frac{\varepsilon_p^3}{(1-\varepsilon_p)^2} \tag{29}$$

where d_p is the effective particle diameter. And the permeability κ^{-1} of the porous media formed by the sponge-like porous hydrogel is hypothetically defined.

The continuity equation is modified as

$$\rho\nabla \cdot u = Q_m \tag{30}$$

where Q_m represents the source term corresponding to mass generation or absorption within the porous medium. The viscous stress tensor K in the porous medium is adjusted and is expressed as

$$\boldsymbol{K} = \mu_l\frac{1}{\varepsilon_p}(\nabla\boldsymbol{u} + (\nabla\boldsymbol{u})^{\mathrm{T}}) - \frac{2}{3}\mu_l\frac{1}{\varepsilon_p}(\nabla \cdot \boldsymbol{u})\boldsymbol{I} \tag{31}$$

were the additional term $\frac{2}{3}\mu_l\frac{1}{\varepsilon_p}(\nabla \cdot \boldsymbol{u})\boldsymbol{I}$ accounts for the dilatational viscosity effects within the porous medium.

(3) Convection-diffusion model

To describe how the concentration of a drug solute evolves over time, taking into account both diffusion and convection, the convection-diffusion equation is written as

$$\frac{\partial C_d}{\partial t} + \nabla \cdot (j_{diff} + j_{conv}) = 0 \quad (32)$$

where $\frac{\partial C_d}{\partial t}$ represents the rate of change in the concentration of drug C_d. The divergence term describes the total flux of drug released into the ambient fluid, where j_{diff} is the diffusion flux and the term $j_{conv} = \boldsymbol{u} \cdot C_d$ is the convective flux accounting for the transport of drug due to the bulk motion of the fluid. According to Fick's law, the diffusion flux j_{diff} is given by

$$j_{diff} = -D_d \nabla C_d \quad (33)$$

where D_d is the diffusion coefficient of the drug. This relationship implies that drug diffuses from regions of higher concentration to regions of lower concentration.

(4) Modified convection-diffusion in porous media

The transport of the drug solute within a porous medium can be described by a modified convection-diffusion equation tailored for porous media:

$$\frac{\partial(\varepsilon_p C_d)}{\partial t} + \frac{\partial(\rho_s C_{s,d})}{\partial t} + \nabla \cdot j_{diff} + \boldsymbol{u} \cdot \nabla C_d = S_d \quad (34)$$

$$j_{diff} = -(D_{D,d} + D_{e,d}) \nabla C_d \quad (35)$$

where $C_{s,d}$ is the concentration of drug in the solid phase, and ρ_s is the density of the solid matrix. S_d represents source or sink terms that may include mass transfer between different phases or any additional sources or sinks of drug. S_d is assumed to be 0 in this study. $D_{D,d}$ and $D_{e,d}$ represent the molecular diffusion coefficient of drug within the porous medium and the effective diffusion coefficient accounting for the porous structure and any additional impediments to diffusion within the medium. Taken together, $(D_{D,d} + D_{e,d})$ constitutes the total diffusion coefficient of the drug to reflect the complexity of diffusion in a porous matrix.

(5) Magnetic field model

The magnetic field model is written as

$$\begin{cases} \nabla \times \boldsymbol{H} = 0 \\ \boldsymbol{H} = -\nabla V_m \\ \boldsymbol{B} = \mu_0 \mu \boldsymbol{H} + \boldsymbol{B}_r \\ \nabla \cdot \boldsymbol{B} = 0 \end{cases} \quad (36)$$

where $\boldsymbol{H}$, V_m, $\boldsymbol{B}$ and $\boldsymbol{B}_r$ are the magnetic field strength, magnetic potential scalar, magnetic flux density and remanent magnetic flux density of the permanent magnets. V_m can be derived from $-\nabla \cdot (\mu_0 \mu \nabla V_m - \boldsymbol{B}_r) = 0$.

Section S14. Fluid velocity and shear rate in DMCG nanopores

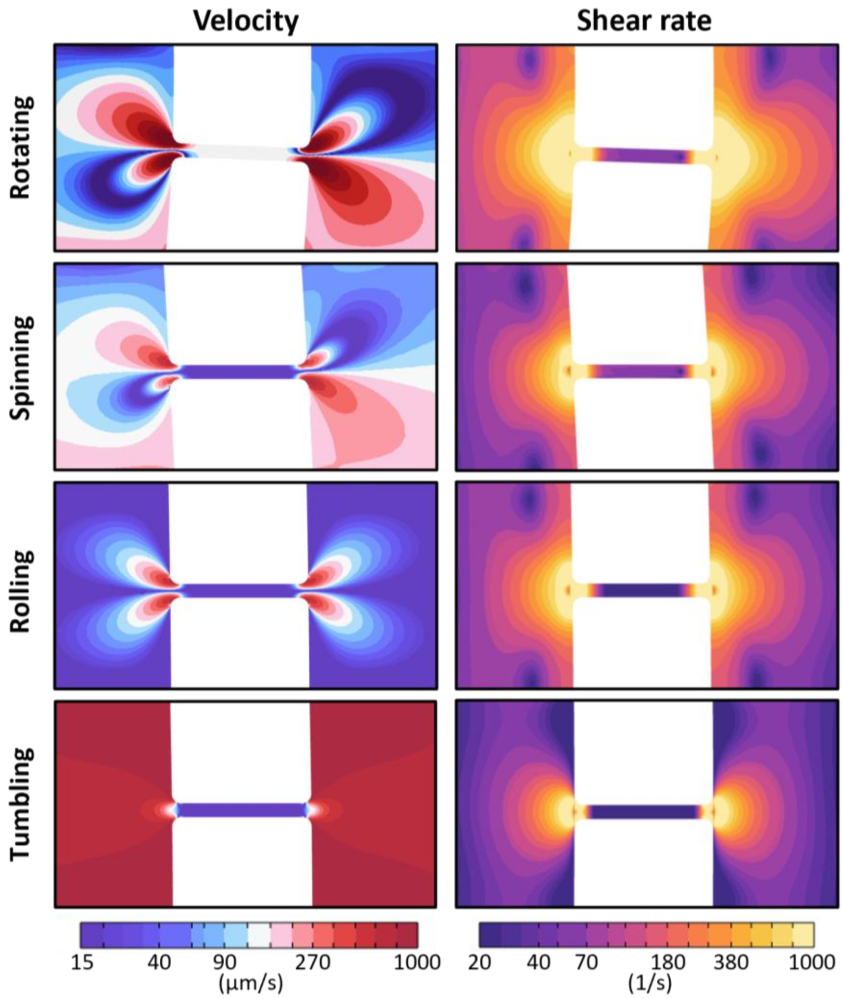

fig. S22. Simulation results of the fluid velocity and shear rate in the nanopores of DMCG under four distinct motion modes.

This figure provides supplementary data for **Fig. 3a** by detailing the fine-scale fluid velocity and shear rate across the DMCG nanopores in rotating, spinning, rolling and tumbling motion regimes.

Section S15. Convection-enhanced drug release

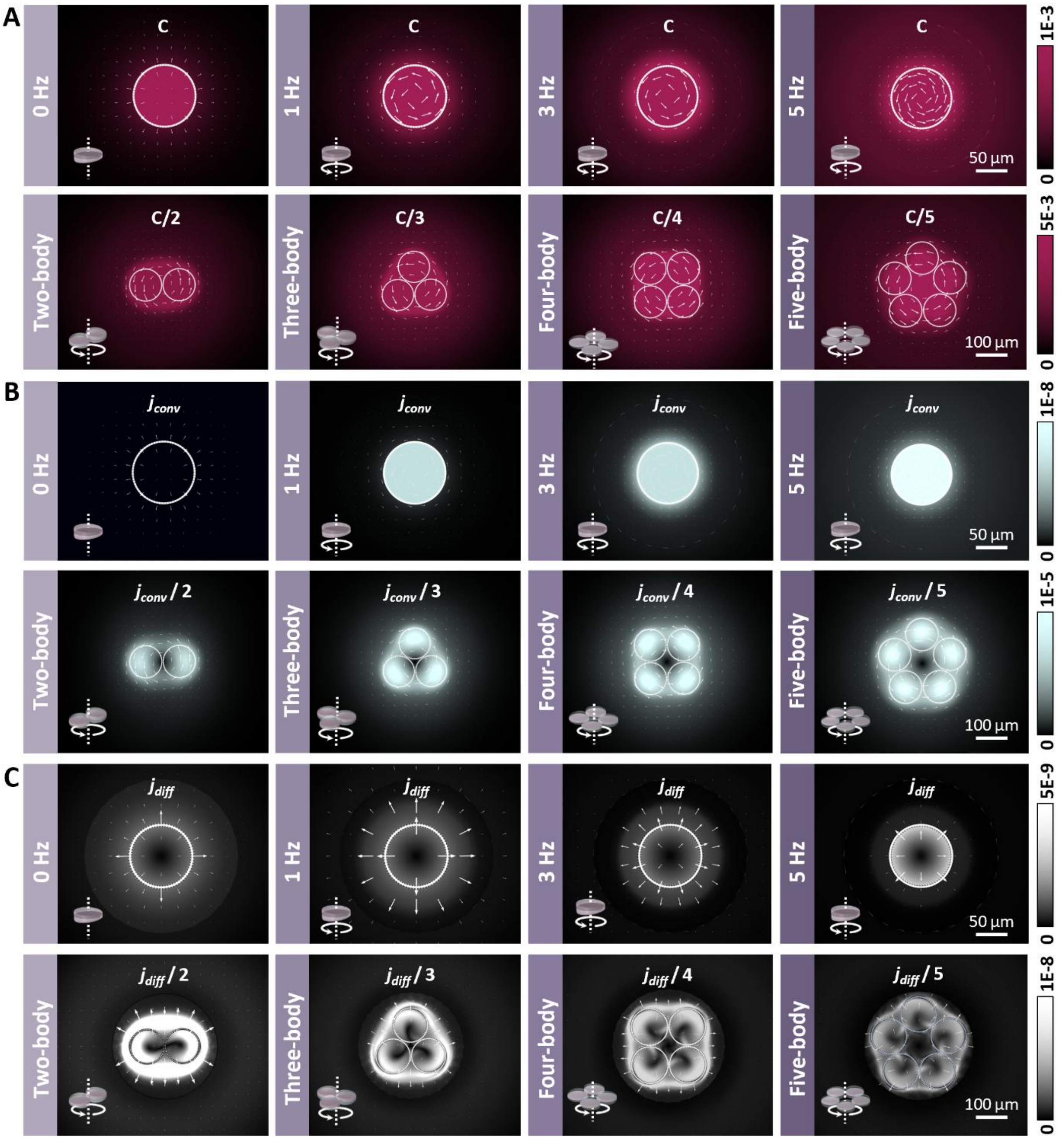

fig. S23. Simulation of the drug concentration field and convective/diffusive fluxes across the surface of single-body and multi-body rotating DMCGs. (A) Drug concentration field (nmol/m³) under varying ration frequencies (0, 1, 3, 5 Hz) and DMCG configurations. Distribution of **(B)** convective flux (nmol/m^2·s) and **(C)** diffusive flux (nmol/m^2·s) across the DMCG nanopores.

This figure provides supplementary data for **Fig. 4c** by illustrating the increased drug release (**fig. S23A**), enhanced convective flux (**fig. S23B**), and suppressed diffusive flux (**fig. S23C**) with increasing DMCG rotation frequency or number of assembling units.

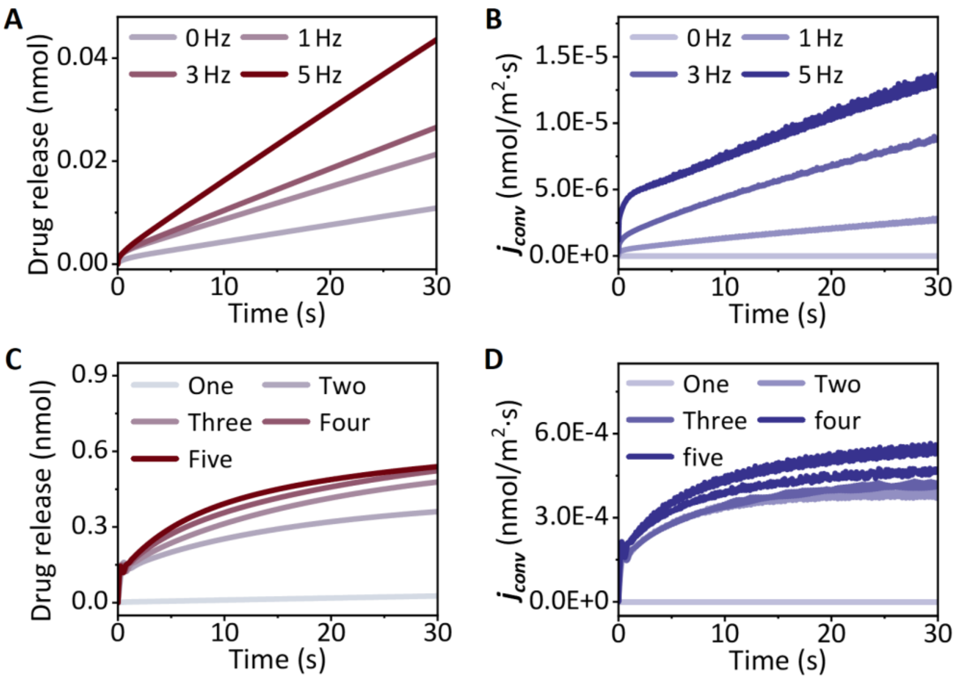

fig. S24. Quantification of simulated drug release and convective flux from single-body and multi-body rotating DMCGs. (**A**) Time-dependent profiles of drug release for single-body DMCG at varying rotation frequencies (0, 1, 3, 5 Hz). (**B**) Convective flux across the nanopores of single-body DMCG rotating at different frequencies. (**C**) Drug release profiles for multi-body DMCGs of different configurations (single versus two-/three-/four-/five-body assembly). (**D**) Convective flux across the nanopores of multi-body DMCGs of different configurations.

Section S16. Convection-enhanced drug penetration into hydrogel

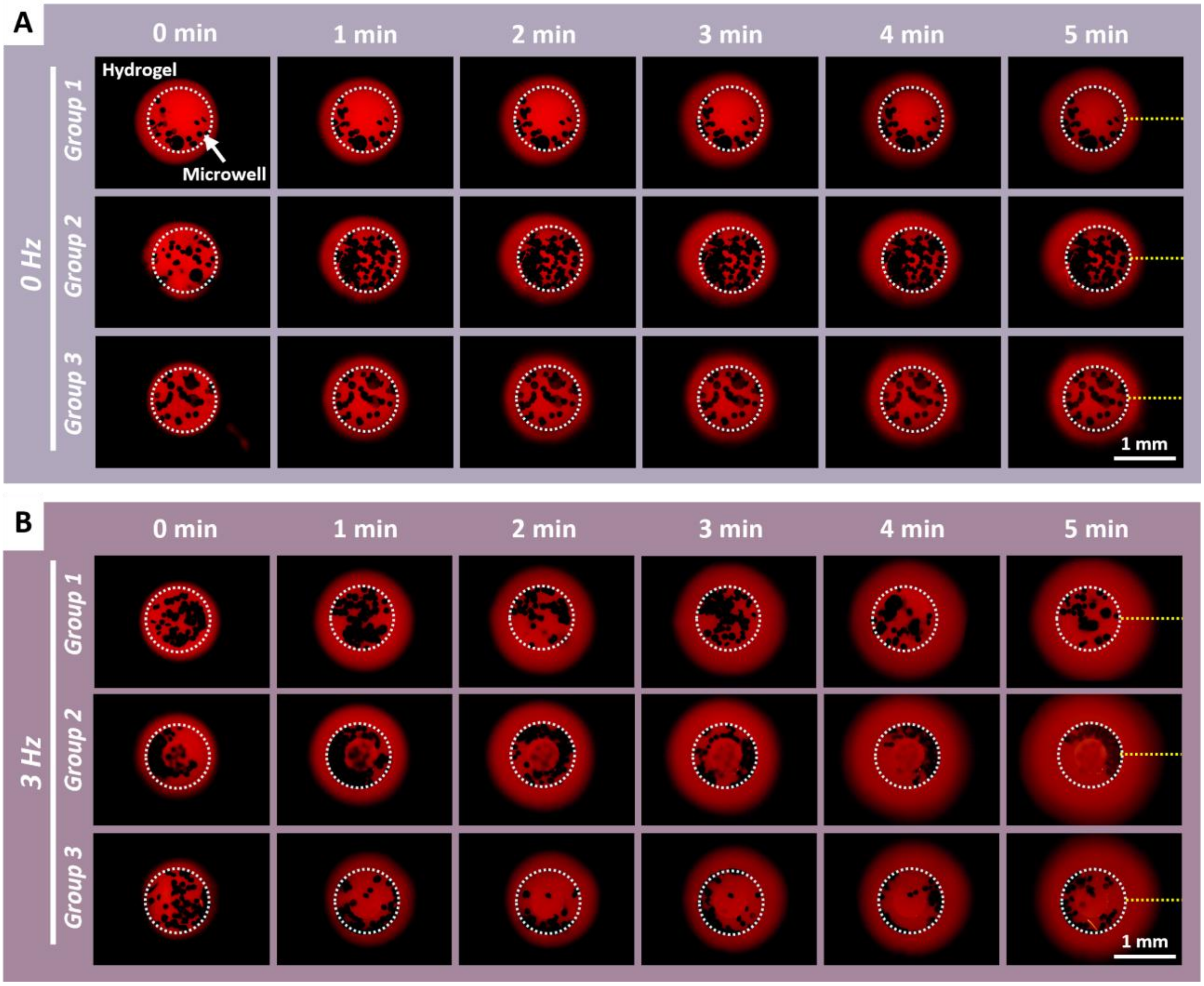

fig. S25. Experiments of DMCG-mediated convective drug penetration into hydrogel. Fluorescence microscopy images depicting the temporal evolution of DOX penetration through the hydrogel boundary in the presence of **(A)** stationary and **(B)** rotating DMCGs (RMF rotation frequency, 3 Hz). n = 3 independently prepared hydrogel microwells per group.

This figure provides supplementary data for **Fig. 4h** by exhibiting the fluorescence outcome of DOX penetration with (**fig. S25A**) and without (**fig. S25B**) DMCG-induced convection effects. The depth of DOX spreading across the hydrogel wall of the microwell is notably larger in the presence of DMCG rotation.

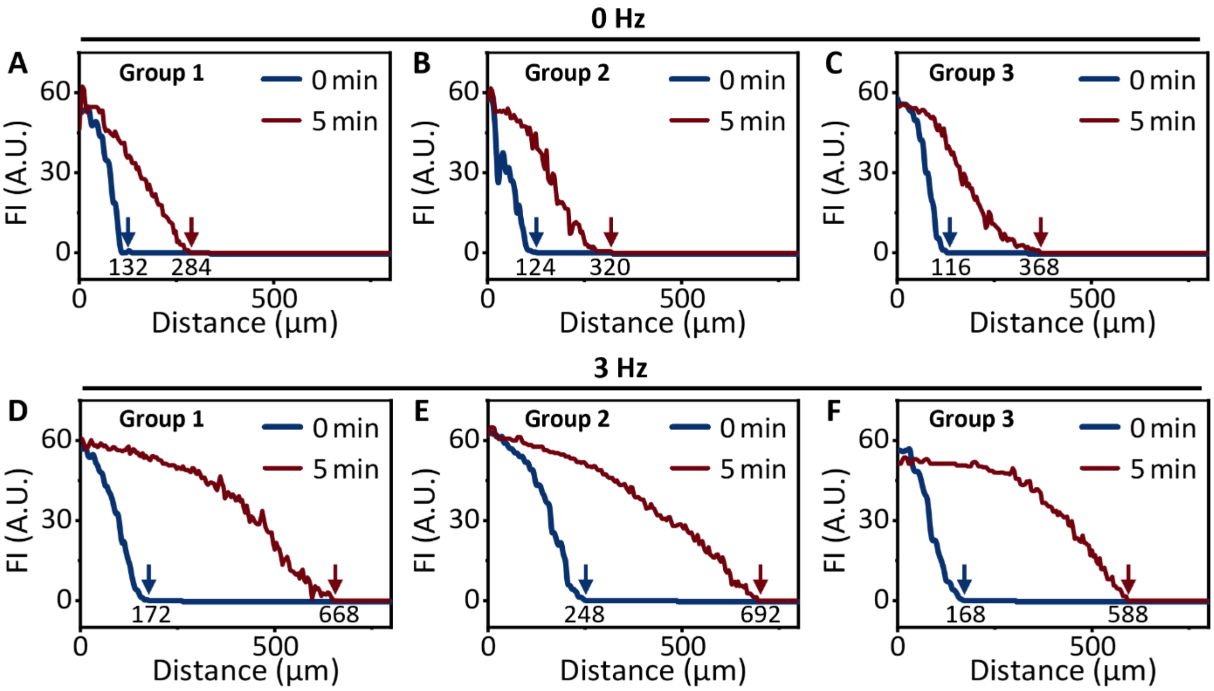

fig. S26. Fluorescence intensity profile across the hydrogel wall along the yellow dashed line. (A), **(B)**, and **(C)** correspond to group 1, 2, and 3 in **fig. S25A**; **(D)**, **(E)**, and **(F)** correspond to group 1, 2, and 3 in **fig. S25B**.

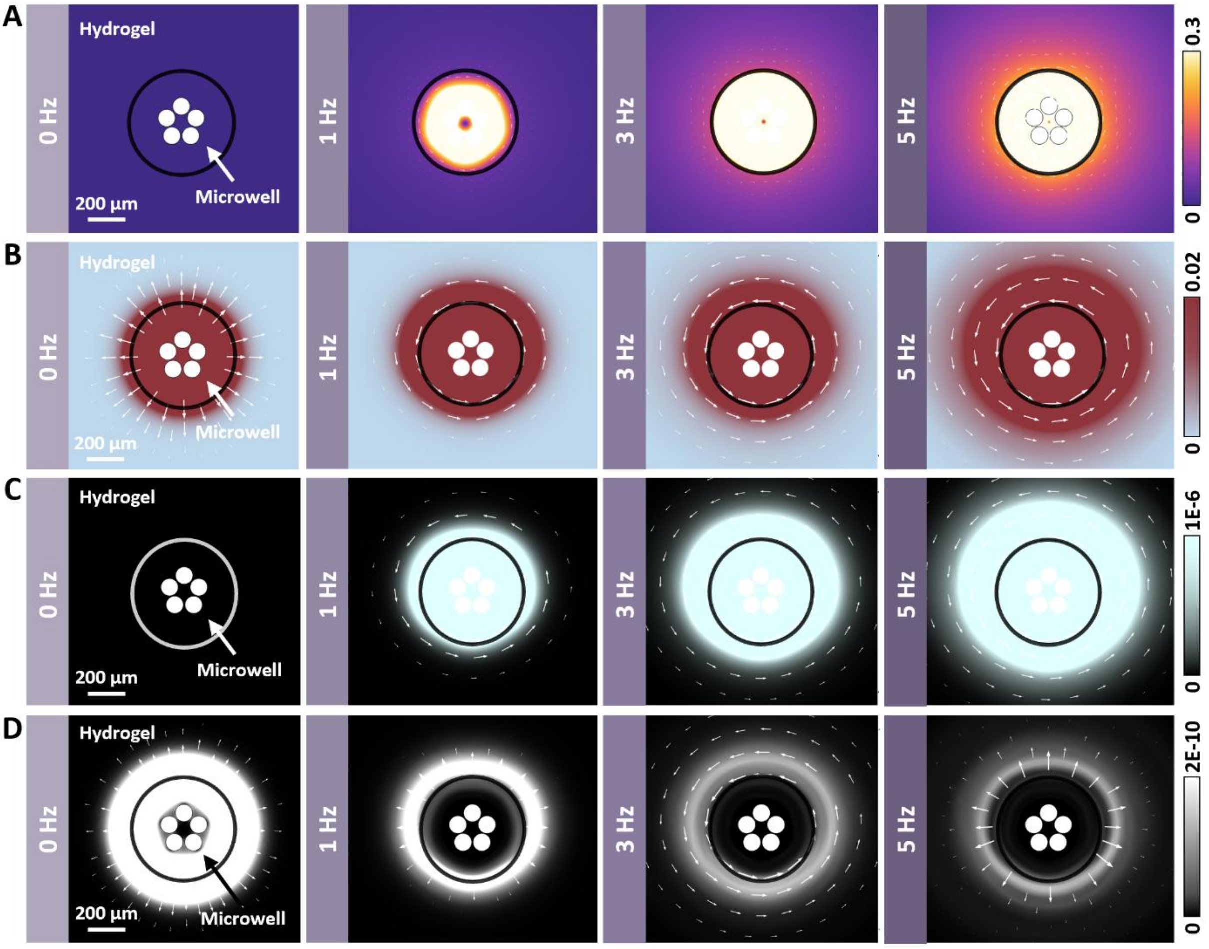

fig. S27. Simulation of the drug concentration field and convective/diffusive fluxes across the boundary of hydrogel wall in the presence of a five-body rotating DMCG assembly. Simulation results of the **(A)** velocity field (mm/s), **(B)** drug concentration field (nmol/m^3), **(C)** convective fluxes (nmol/m^2·s) and **(D)** diffusive fluxes (nmol/m^2·s) with the DMCG assembly rotating at frequencies 0, 1, 3 and 5 Hz.

This figure provides supplementary data for **Fig. 4h** by illustrating the increased flow velocity (**fig. S27A**), accelerated drug penetration (**fig. S27B**), enhanced convective fluxes (**fig. S27C**), and suppressed diffusive fluxes (**fig. S27D**) across the hydrogel wall of the microwell as the rotation frequency of DMCGs increases.

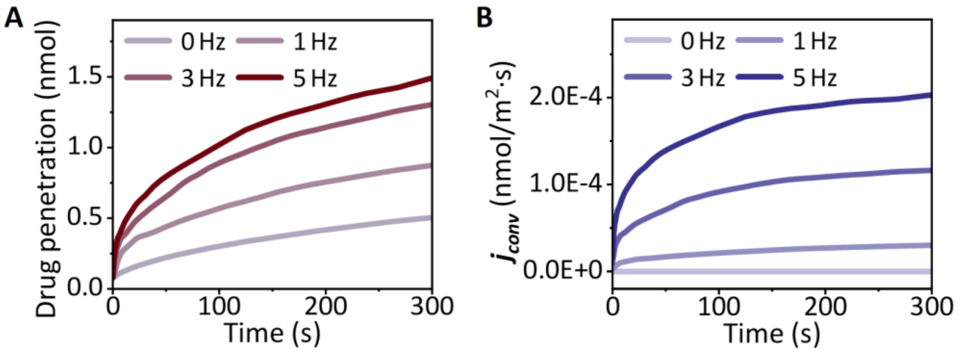

fig. S28. Quantification of the simulated drug-penetration quantity and convective flux in fig. S27. (A) Drug-penetration quantity. **(B)** Convective flux.

Section S17. Convection-enhanced drug penetration into tumor spheroid

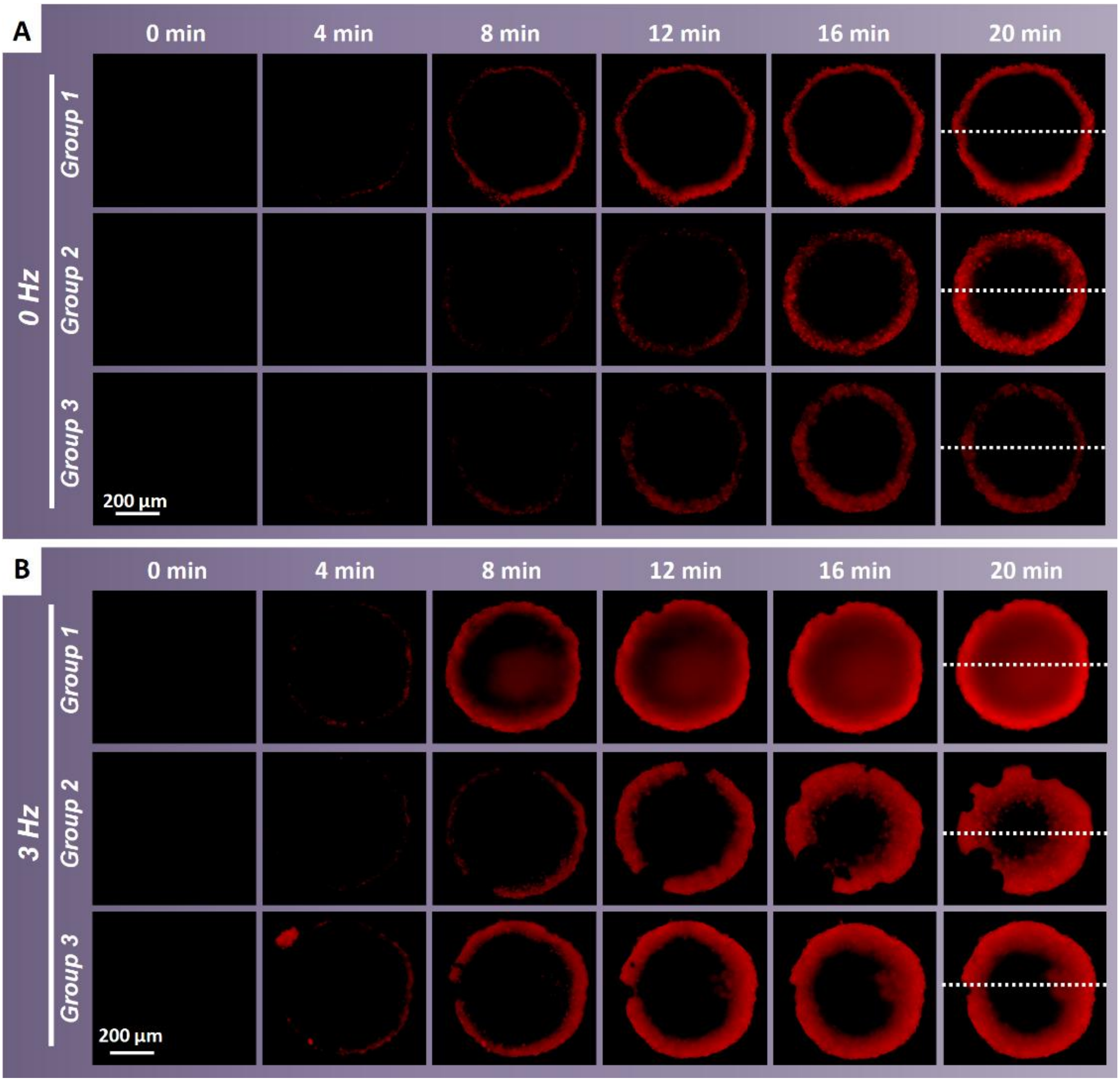

fig. S29. Experiments of DMCG-induced convective drug penetration into tumor spheroids. Fluorescence images depict the temporal penetration of DOX through the tumor spheroid (n=3) in the presence of **(A)** stationary and **(B)** magnetically-actuated DMCGs (rotation frequency, 3 Hz). n=3 independent tumor spheroids per group.

This figure provides supplementary data for **Fig. 4l** by exhibiting the fluorescence outcome of DOX penetration with (**fig. S29A**) and without (**fig. S29B**) DMCG-induced convection effects. The depth of DOX spreading inward the tumor spheroid center is notably larger in the presence of DMCG rotation.

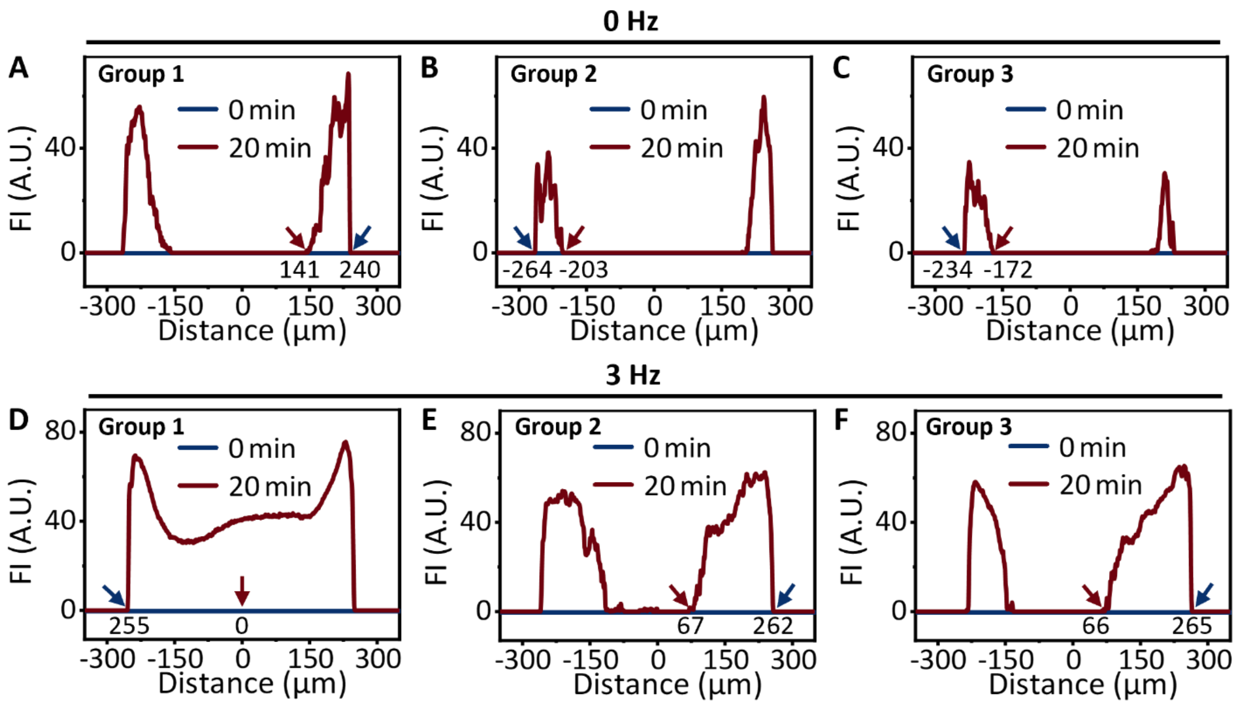

fig. S30. Fluorescence intensity profile across the tumor spheroid along the white dashed line. **(A)**, **(B)**, and **(C)** correspond to group 1, 2, and 3 in **fig. S29A**; **(D)**, **(E)**, and **(F)** correspond to group 1, 2, and 3 in **fig. S29B.**

Section S18. Interaction of DMCGs with simulated tumor interface

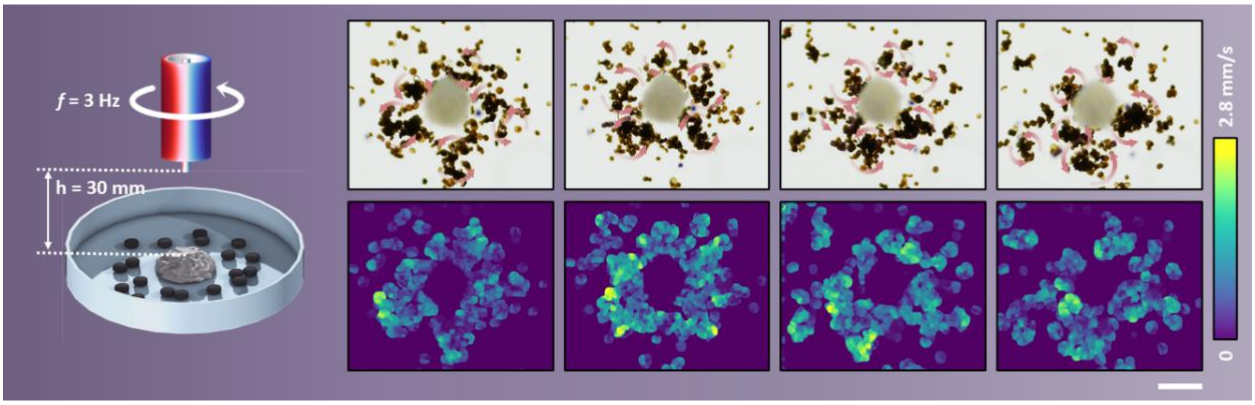

fig. S31. Experiments of magnetically controlled DMCG clusters swirling near the tumor spheroid. (left) Schematic of the experimental setup. **(top right)** Representative bright-field images of the swirling clusters in close contact with the tumor spheroid. **(bottom right)** Corresponding optical-flow analysis showing the boundary velocities of the clusters near the tumor. Scale bar, 500 μm.

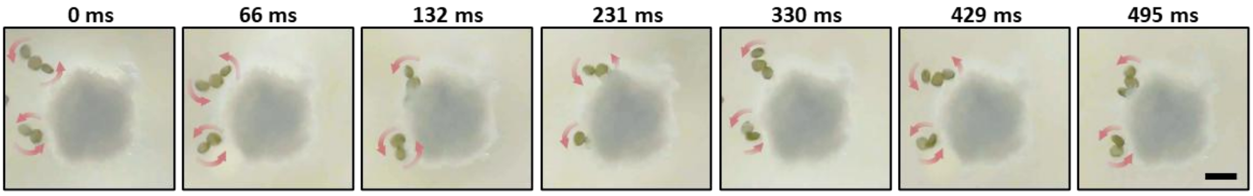

fig. S32. Demonstration of the non-adhesive behavior of DMCG clusters upon collision with the tumor spheroid surface. Time-elapse sequence shows sustained mobility. Scale bar, 200 μm.

Near-wall swarm swirling dynamics under typical operating conditions with a much larger "tumor" model (3D-printed with polylactic acid, hemispherical *ca.* 5 mm, beyond the size limit of cultured tumor spheroid). Optical-flow analysis revealed distinct velocity distributions (**fig. S33**):

Case 1: (Magnet distance 30 mm, rotation frequency 1 Hz) The DMCGs form a tightly packed aggregate, and the swarm maintains its position in situ throughout the rotation (**fig. S33A**). The velocity contour shows that flow velocities within the swarm is fairly uniform, indicating that the fluidic forces generated at such low rotation frequency were weak and possibly counteracted by the magnetic dipole forces, which results in a stable swarm.

Case 2: (Magnet distance 30 mm, rotation frequency 5 Hz) The DMCGs form a loosely structured aggregate, and the swarm gradually moves toward the tumor wall (**fig. S33b**). Upon contact with the wall, individual DMCGs near the wall leave and rejoin the main body through internal flow circulation of the swarm. Additionally, there is a clear velocity difference between the near-wall and far-wall regions. Although the structure of the swarm is overall sparse, inherent magnetic dipole interactions between individual clusters still manage to maintain the swarm integrity as the swarm approaches the tumor, preventing it from being disassembled by flow disruptions or wall collisions.

Case 3: (Magnet distance 40 mm, rotation frequency 3 Hz) The DMCGs form multiple small aggregates in coordinated motion, and the swarm steadily approaches the wall (**fig. S33C**). Upon contact with the tumor, a certain degree of reduction in the swarm velocity occurs, presumably due to energy dissipation caused by local collisions. Nonetheless, the overall swarm maintains coordinated motion without being disrupted.

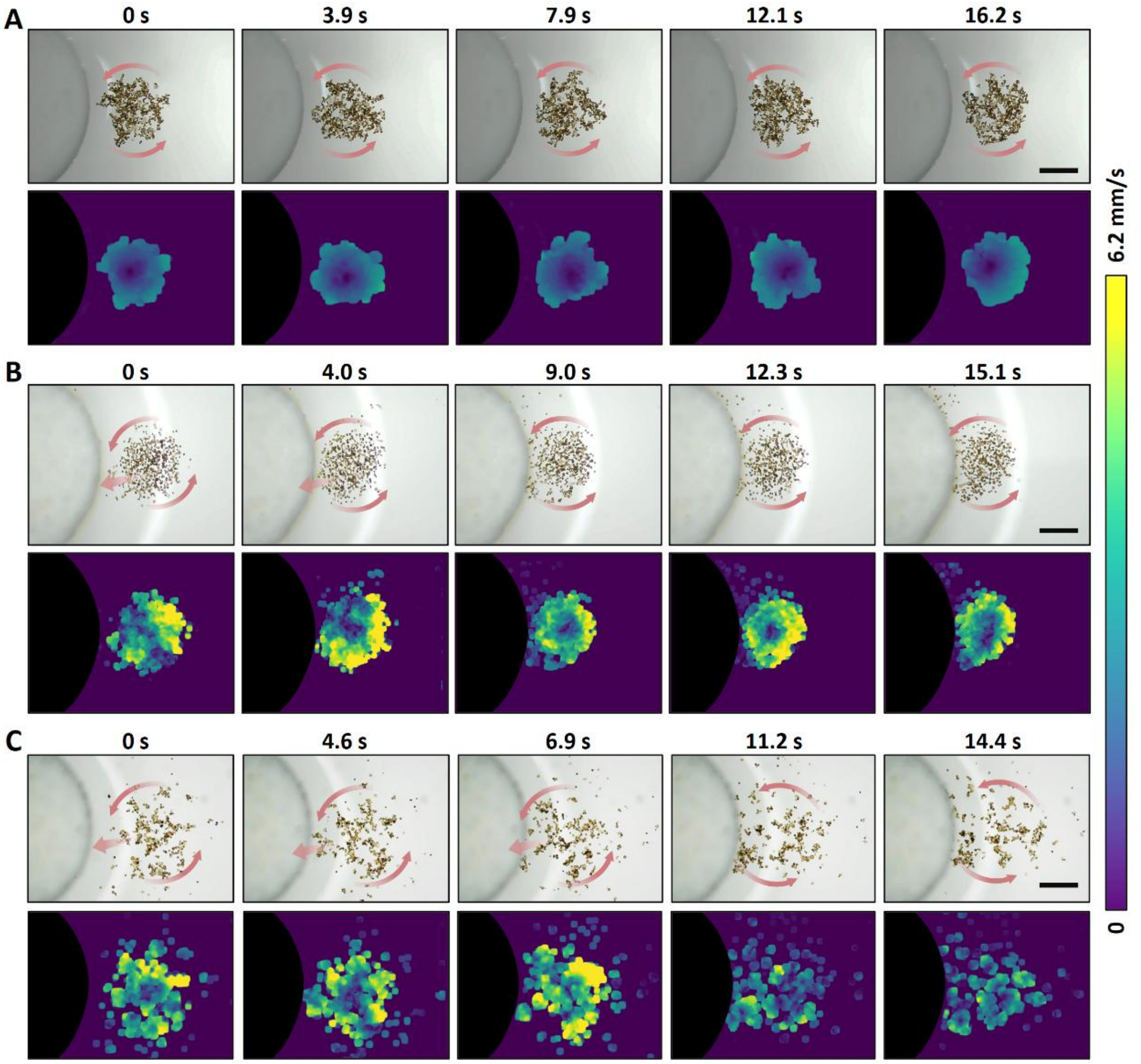

fig. S33 Stability of DMCG swirling near a simulated tumor boundary under varying operating conditions. (A) Tightly-packed single-swarm swirling. **(B)** Loosely-connected single-swarm swirling. **(C)** Coordinated-swarm swirling of multiple small clusters. Scale bar, 1 mm.

Section S19. Cytotoxicity assay

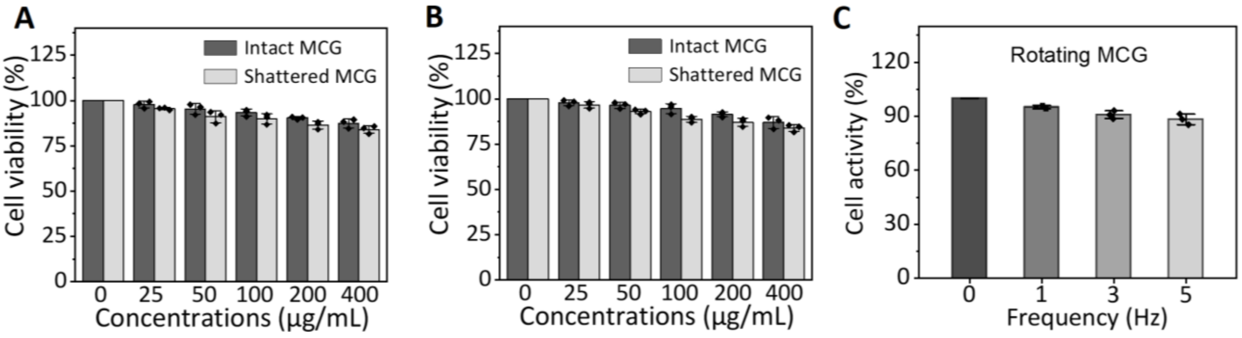

fig. S34. Evaluation of DMCG cytotoxicity and its motion-induced mechanical damage. Cytotoxicity assays for (A) bladder cancer tumor cells (MTB-2 G3) and (B) immortalized human ureteral epithelial cells (SV-HUC-1). (C) Cell viability subject to MCG rotation treatment with rotation frequencies of 0, 1, 3, and 5 Hz. Data are presented as mean ± s.d. from n = 3 independent experiments.

Cell culture: Bladder cancer tumor cells (MTB-2 G3) and immortalized human ureteral epithelial cells (SV-HUC-1) were used as model cells for the cytotoxicity assay of MCG. MTB-2 G3 cells were cultured in RPMI-1640 medium containing 10% FBS and dual antibiotics, and SV-HUC-1 cells were cultured in DMEM/F12K medium with 10% FBS and dual antibiotics. Both cell types were maintained in a 37°C incubator with 95% oxygen and 5% CO_2 until reaching 85%-90% confluence for subsequent experiments.
Cytotoxicity assay: *In vitro* cytotoxicity studies were performed using the model cells cultured above. Concentration gradients (25, 50, 100, 200, and 400 μg/mL) of intact and fragmented MCGs, alongside a control group without MCGs, were established in a 96-well plate with six duplicate wells for each concentration. Cells (10,000 cells/μL) were seeded in 100 μL per well. The plate was then placed in a 5% CO_2 cell culture incubator at 37°C for 12-24 hours to ensure cell adhesion. Subsequently, the culture medium was aspirated, resulting in a mixture containing intact or shattered MCGs for the experimental groups and a MCG-free pure medium for the control group. The 96-well plate was incubated for additional 24 hours at 37°C in a 5% CO_2 cell culture incubator.
MCG motion-induced cell mechanical damage: The MTB-2 G3 cells were seeded with a density of 10,000 cells/μL in ten wells of an Elisa plate, each containing 100 μL. Subsequently, 100 μL of MCG-PBS solution (1 mg/mL) was added to each well. The plate was then positioned within the workspace of the Helmholtz coil (**fig. S3**) and a rotating magnetic field of 10 mT was generated to induce DMCG rotation. The rotation frequency of the rotating magnetic field was varied in the range of (0, 1, 3, 5 Hz), with each group exposed for 20 minutes.
Cell viability evaluation: To evaluate the cell viability, 10 μL CCK-8 solution was directly added to each well. The culture plate with CCK-8 was then placed in a 37°C, 5% CO_2 incubator and incubated for 1-4 hours. Subsequently, a microplate reader was employed to measure the concentration of each well at a wavelength of 450 nm (OD value). Then the data were processed, and a proliferation curve was generated to analyze cell survival rate. As shown in **fig. S34**, the cell viability of MTB-2 G3 (**fig. S34A** and SV-HUC-1 (**fig. S34B**) cultured with both intact and fragmented MCGs at stationary state exceeded 85%, indicating robust biosafety of the MCG. After treatment with the rotating MCGs, the cell survival rate still surpassed 85% (**fig. S34C**), suggesting that it has no significant impact on cellular activity.

Section S20. Ultrasound signal intensity of DMCG

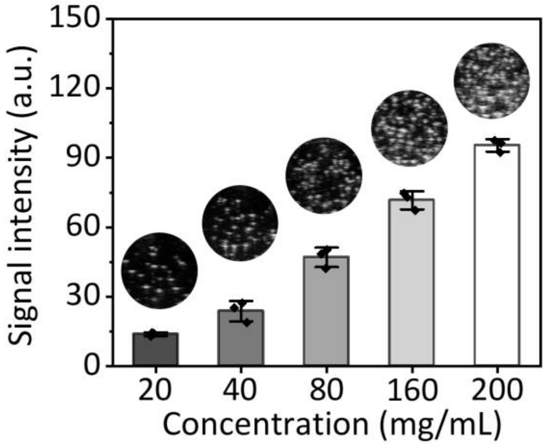

fig. S35. Ultrasound signal intensity of DMCG at different concentrations. n = 3 independent samples per group.

Section S21. Robotic magnet system with nRSM mounted

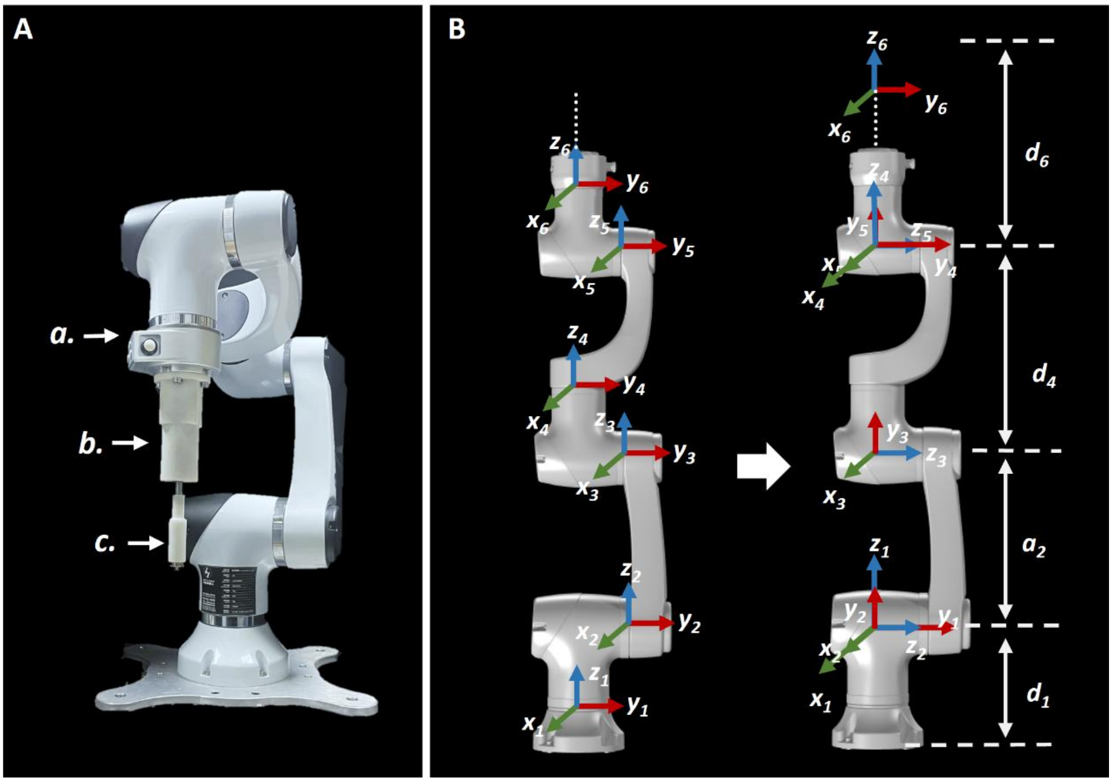

fig. S36. Robotic magnet system (RMS). (A) Physical components of the RMS. **(B)** Schematic illustration of the coordinate transformation for the robotic arm.

The RMS (**fig. S36A**) consists of **a**) a commercial six-axis robotic arm (E03, Hansrobot, China), **b**) an *in-house* wifi-communicating DC motor, and **c**) an *in-house* permanent magnet setup (nRSM, see **fig. S9**). We transform the coordinates of the robotic arm to obtain a simplified model (**fig. S36B**) and the associated Denavit-Hartenberg (D-H) parameters (**Table. S3**).

Table S3. D-H parameters of the robotic arm

Joint i	a_{i-1}	α_{i-1}	d_i	θ_i
**1**	0	90°	d_1	θ_1
**2**	a_2	0	0	θ_2
**3**	0	90°	0	θ_3
**4**	0	-90°	d_4	θ_4
**5**	0	90°	0	θ_5
**6**	0	0	d_6	θ_6

The D-H parameters typically involve four values: a_{i-1}, α_{i-1}, d_i, and θ_i. a_{i-1} is the distance along the common normal between the current joint axis i and the previous joint axis $i-1$, measured to the next joint axis. α_{i-1} is the angle about the common normal between the current joint axis i and the previous joint axis $i-1$. d_i is he distance along the current joint axis i between the common normal and the point where the next joint axis $i+1$ intersects the common normal. θ_i is the angle about the current joint axis i between the common normal and the line of intersection with the next joint axis $i+1$. To achieve programmable motion of the robotic arm, we plan the desired end coordinates of the arm. Subsequently, the corresponding changes of angle for each joint are determined through inverse kinematics operations.

The motion of the RMS is represented as

$$ {}^{1}_{6}T \begin{bmatrix} \boldsymbol{n}_x & \boldsymbol{o}_x & \boldsymbol{a}_x & p_x \\ \boldsymbol{n}_y & \boldsymbol{o}_y & \boldsymbol{a}_y & p_y \\ \boldsymbol{n}_z & \boldsymbol{o}_z & \boldsymbol{a}_z & p_z \\ 0 & 0 & 0 & 1 \end{bmatrix} = {}^{0}_{6}T = {}^{0}_{1}T \times {}^{1}_{2}T \times {}^{2}_{3}T \times {}^{3}_{4}T \times {}^{4}_{5}T \times {}^{5}_{6}T \quad (37) $$

where $\boldsymbol{n}$, **o**, **a** represent the direction vectors of x-, y- and z-axes of the end coordinate system in the base coordinate system, and $\boldsymbol{p}$ represents the end coordinate of the robotic arm in the base coordinate system. To simplify the expression of formula, we abbreviate $sin\,\theta_i$ as s_i, $cos\,\theta_i$ as c_i, and $sin\ (\theta_i + \theta_j)$ as s_{ij}, so the below can be derived:

$$
\begin{cases}
\boldsymbol{n}_x = s_1c_4s_6 - c_1c_6s_4s_{23} - c_1c_6s_5s_{23} + c_5c_6s_1s_4 + c_1c_{23}c_4c_5c_6 \\
\boldsymbol{n}_y = -s_1c_6s_5s_{23} - c_1s_4c_5c_6 + s_1c_{23}c_4c_5c_6 - s_6c_1c_4 - s_1s_4s_6c_{23} \\
\boldsymbol{n}_z = -c_{23}s_5c_6 - s_{23}c_4c_5c_6 + s_4s_6s_{23} \\
\boldsymbol{o}_x = s_1c_6c_4 - s_1s_4c_5s_6 - c_1c_{23}s_4c_6 + s_5s_6c_1s_{23} - c_5s_6c_4c_1c_{23} \\
\boldsymbol{o}_y = s_6s_5s_1s_{23} + s_6c_5c_1s_4 - s_6c_5c_4s_1c_{23} - c_6c_1c_4 - c_6s_4s_1c_{23} \\
\boldsymbol{o}_z = c_{23}s_5s_6 - s_1s_4s_5 - c_{23}c_4c_1s_5 \\
\boldsymbol{a}_x = -s_1s_4s_5 - c_1c_{23}c_4s_5 - c_1s_{23}c_5 \\
\boldsymbol{a}_y = s_4s_5c_1 - c_5s_1s_{23} - s_1c_{23}c_4s_5 \\
\boldsymbol{a}_z = c_4s_5s_{23} - c_{23}c_5 \\
p_x = a_2c_1c_2 - d_4c_1s_{23} - d_6s_1s_4s_5 - d_6c_1c_{23}c_4s_5 - d_6c_1s_{23}c_5 \\
p_y = a_2s_1c_2 - d_4c_1s_{23} - d_6c_1s_4s_5 - d_6s_1c_{23}c_4s_5 - d_6s_1s_{23}c_5 \\
p_z = d_1 - a_2s_2 - d_4c_{23} - d_6c_5c_{23} + d_6c_4s_{23}s_5
\end{cases}
\tag{38}
$$

and angle of each joint of the arm can be calculated as

$$\theta_1 = arctan(p_y - d_6\boldsymbol{a}_y/p_x - d_6\boldsymbol{a}_x) \tag{39}$$

$$\theta_2 = arctan\frac{(d_1-p_z+d_6)\boldsymbol{a}_z}{\pm\sqrt{(a_2-d_4s_3)^2+(d_4c_3)^2-((d_1-p_z+d_6)\boldsymbol{a}_z)^2}} - arctan\frac{d_4c_3}{a_2-d_4s_3} \tag{40}$$

$$\theta_3 = arctan\frac{\sqrt{d_4^2-(d_4s_3)^2}}{-d_4s_3} \pm \frac{\pi}{2} \tag{41}$$

$$\theta_4 = arctan\frac{\boldsymbol{a}_xs_1-\boldsymbol{a}_yc_1}{c_{23}(\boldsymbol{a}_xc_1+\boldsymbol{a}_ys_1)-\boldsymbol{a}_zs_{23}} \tag{42}$$

$$\theta_5 = -\,arctan\frac{\boldsymbol{a}_zs_{23}c_4-\boldsymbol{a}_x(c_1c_{23}c_4+s_1s_4)-\boldsymbol{a}_y(s_1c_{23}c_4-c_1s_4)}{\boldsymbol{a}_xc_1s_{23}+\boldsymbol{a}_ys_1s_{23}+\boldsymbol{a}_zc_{23}} \tag{43}$$

$$\theta_6 = arctan\frac{\boldsymbol{n}_zs_{23}s_4-\boldsymbol{n}_x(c_1c_{23}s_4-s_1c_4)-\boldsymbol{n}_y(s_1c_{23}s_4+c_1c_4)}{\boldsymbol{o}_zs_{23}s_4-\boldsymbol{o}_x(s_4c_1c_{23}-s_1c_4)-\boldsymbol{o}_y(c_{23}s_1s_4-c_4c_1)} \tag{44}$$

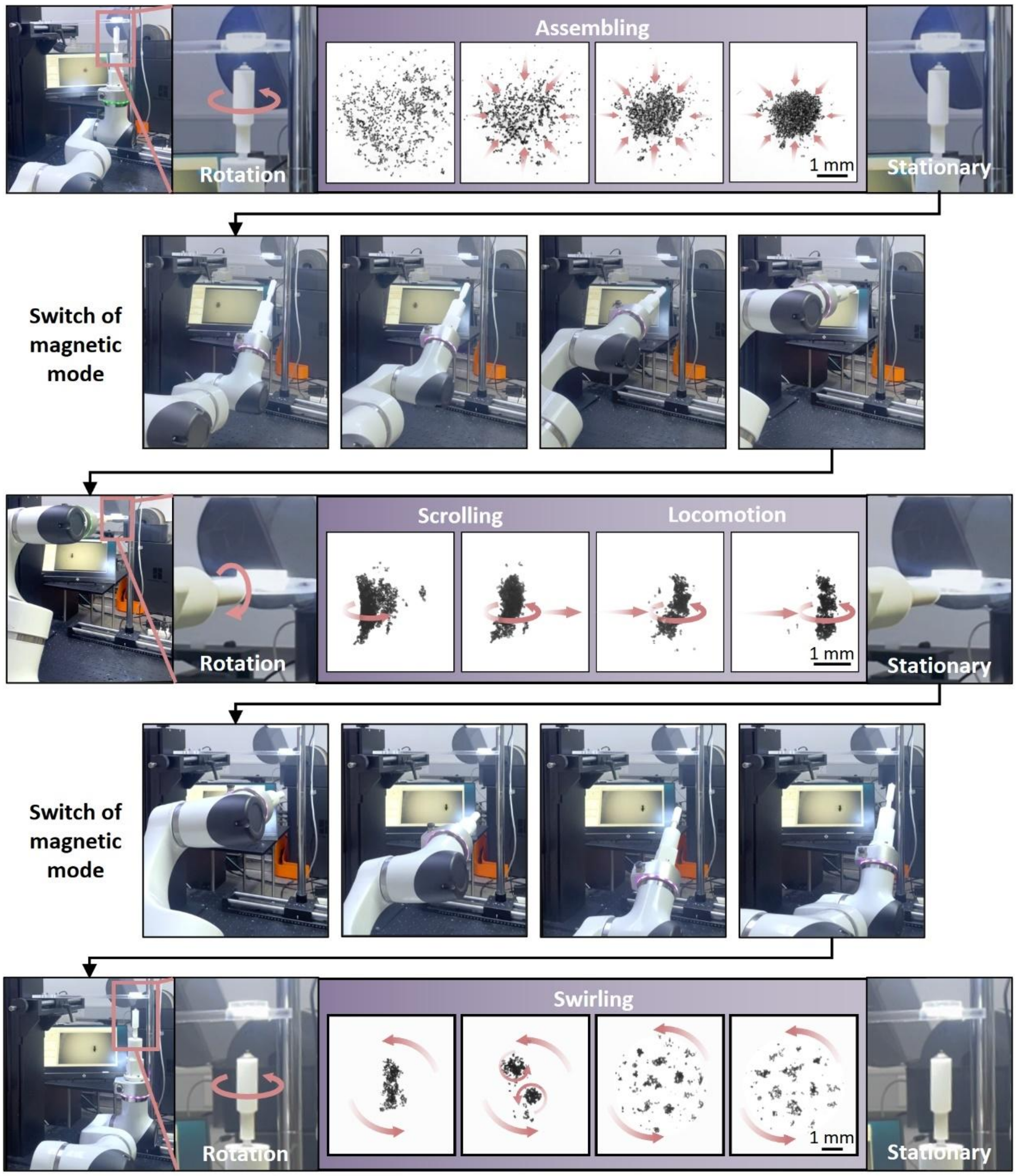

fig. S37 Time-lapse sequence of the robotic magnet system (RMS) pose and gait for actuation and multimodal control of the DMCG swarm's reconfiguration and locomotion.

Section S22. Framework of image-feedback machine-intelligent control

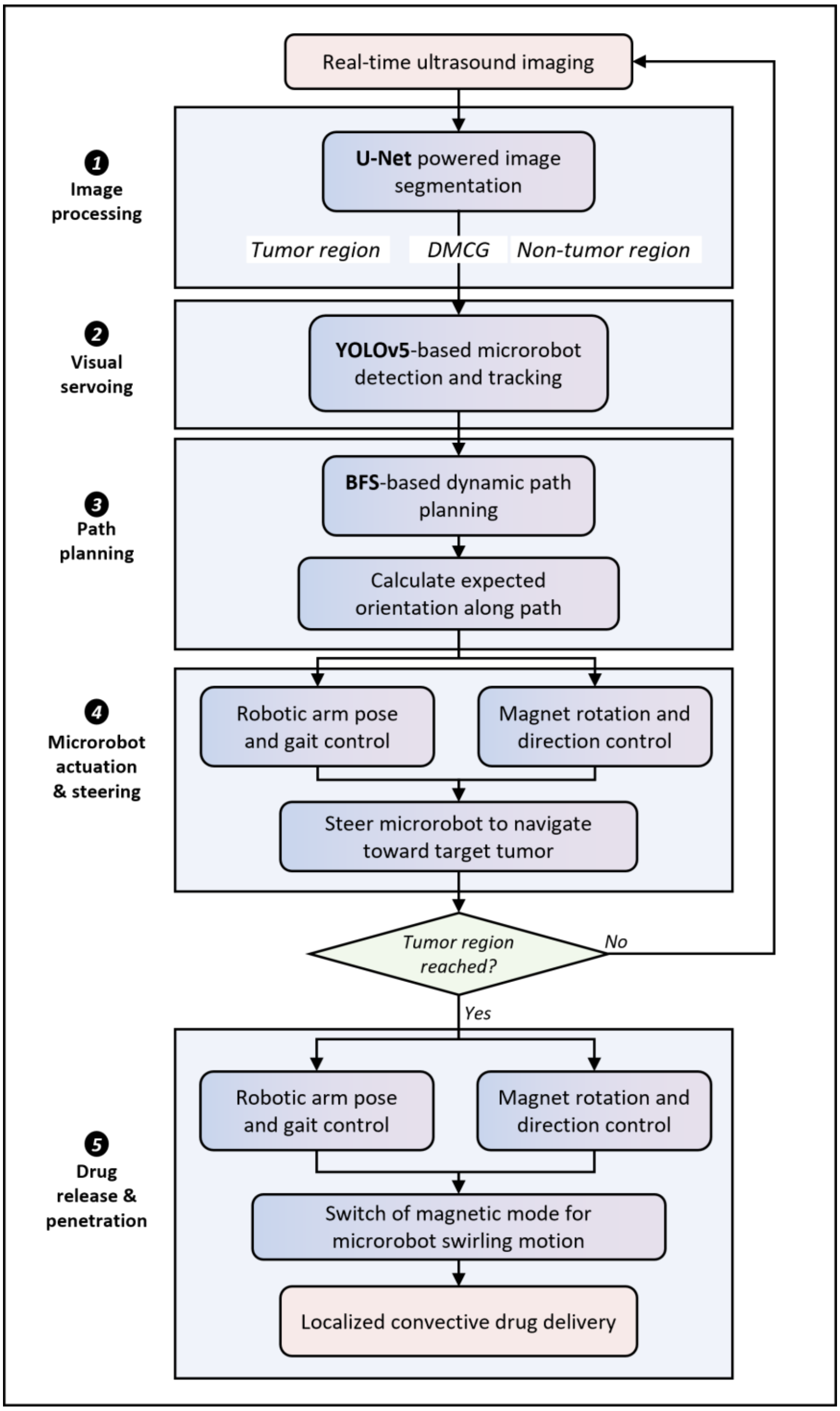

fig. S38. Workflow for machine-learning empowered intelligent control of DMCG microrobots for tumor targeting and drug delivery.

The learning-based framework integrates (1) real-time ultrasound image acquisition & processing, (2) visual servoing, (3) path planning, (4) magnetic actuation and steering, and (5) drug release and penetration for active drug delivery to targeted tumor.

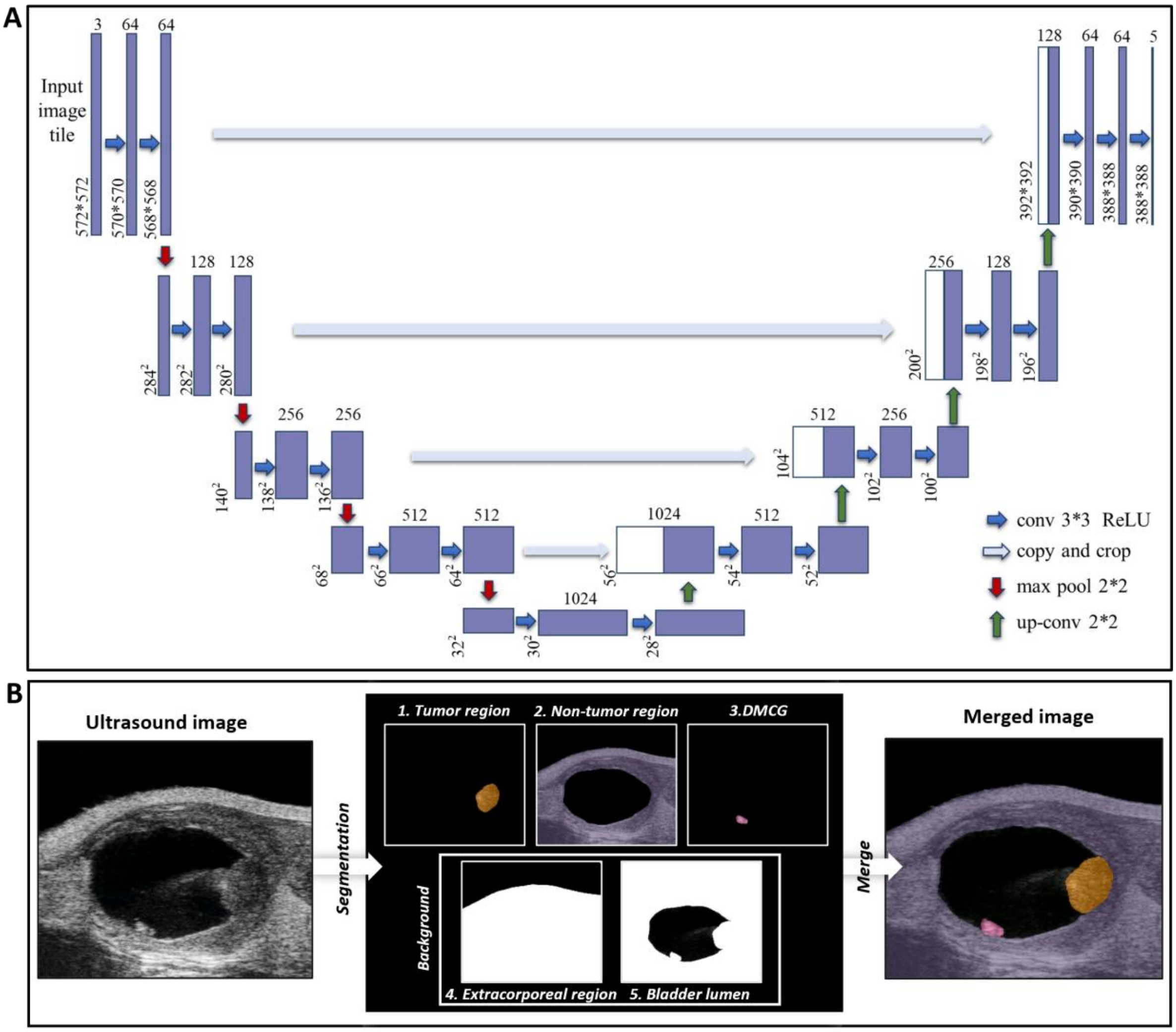

fig. S39. U-Net algorithm for ultrasound image segmentation. (A) Schematic of the U-Net architecture. **(B)** Exemplary process for the image segmentation.

(1) Image acquisition and preprocessing: In the first stage, real-time ultrasound imaging monitors the location of the DMCG swarm relative to the tumor site. A customized data transmission system is employed to ensure synchronization of imaging data with the host computer processing the data. A U-Net algorithm is then applied to segment the acquired ultrasound images.

The U-Net algorithm (**fig. S39A**) adopts a symmetric encoder-decoder architecture design for biomedical image segmentation. It consists of a contracting path (encoder) and an expanding path (decoder), with skip connections that bridge corresponding levels to preserve spatial information. In the contracting path, the input image undergoes successive applications of convolutional layers (3×3, ReLU activation) and max-pooling operations (2×2) to capture high-level semantic features. The downsampling steps reduce the spatial dimensions while increasing the feature channels, to enhance the algorithm ability for capturing complex structures, e.g., tumor or DMCG swarm. The expanding path then performs up-convolution (2×2) to restore the spatial resolution. The skip connections directly transfer feature maps from the encoder to the decoder, allowing the algorithm to combine low-level spatial details with high-level semantic information, which is crucial for accurate boundary delineation in ultrasound images (fairly low signal-to-noise ratio). For bladder ultrasound segmentation, the

algorithm outputs a multi-channel probability map, assigning each pixel a likelihood score assigned to one of five categories: tumor region, non-tumor region (i.e., healthy perivesical tissue), DMCG swarm, extracorporeal region and bladder lumen (**fig. S39B**). For clarity of visualization, only the tumor region, non-tumor region, and DMCG swarm are labeled here in colored masks. The masked images are merged into the processing pipeline as the input for downstream analysis.

(2) Visual servoing: The DMCG swarm is detected and tracked in real time using a YOLOv5-based algorithm. Through time-elapse comparison of the microrobot position with the target tumor location, the system updates the microrobot motion and posture in a closed-loop manner to facilitate precise control of the microrobot locomotion under dynamic conditions.

(3) Path planning: A breadth-first search (BFS)–based algorithm is employed for dynamic path planning. The algorithm takes in the up-to-date segmentation information for rapid response to changes in the environment (e.g., boundaries or obstacles) through real-time calculation and refinement of optimal routes and expected orientation angles.

(4) Microrobot actuation and steering: A robotic arm provides pose and gait control upon commands to facilitate precise positioning of the microrobot. Concurrently, the external magnetic field generated by the permanent magnet mounted on the robotic arm enables required microrobot maneuvers such as forward locomotion or swirling in situ.

(5) Drug release and penetration: Once the tumor site is reached, localized drug delivery is activated through switching the magnetic control mode so the DMCG swarm performs swirling for convective drug release and penetration in situ.

Section S23. *In vitro* assessment of convection-enhanced drug penetration

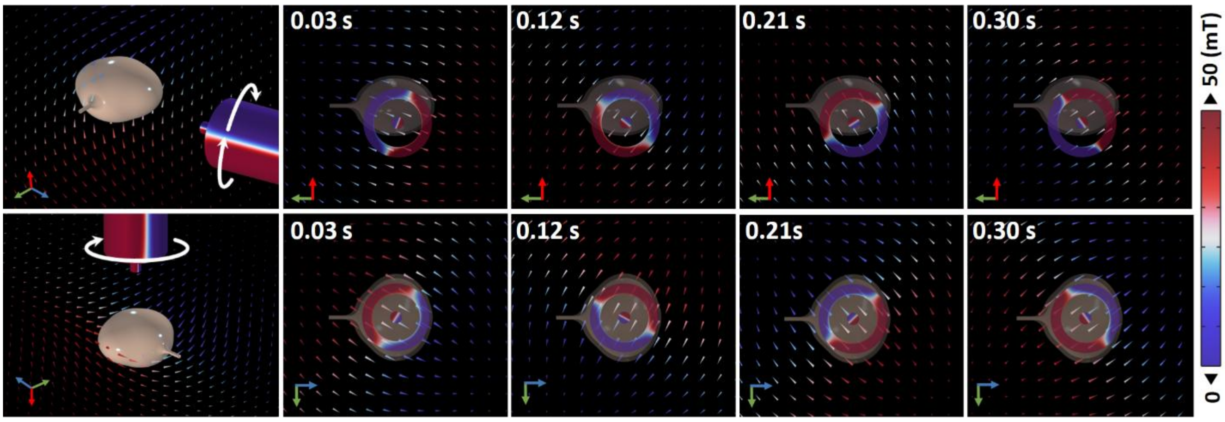

fig. S40. Simulation of the magnetic potential scalar field arising from a rotating permanent magnet across an artificial mouse bladder model.

This figure provides the simulation data of the magnetic potential scalar field generated by a rotating permanent magnet. When the magnet is located perpendicular aside or above the 3D bladder, its rotation generating a rotating magnetic field which in the central section of the bladder resembles that of B_{t3} generated by the Helmholtz coils presented earlier (**fig. S4**). Such resemblance suggests the feasibility of utilizing permanent magnets mounted on a robotic arm as an alternative of electromagnetic coils for more flexible actuation and control of DMCG to perform similar rolling and rotating motions in large workspace.

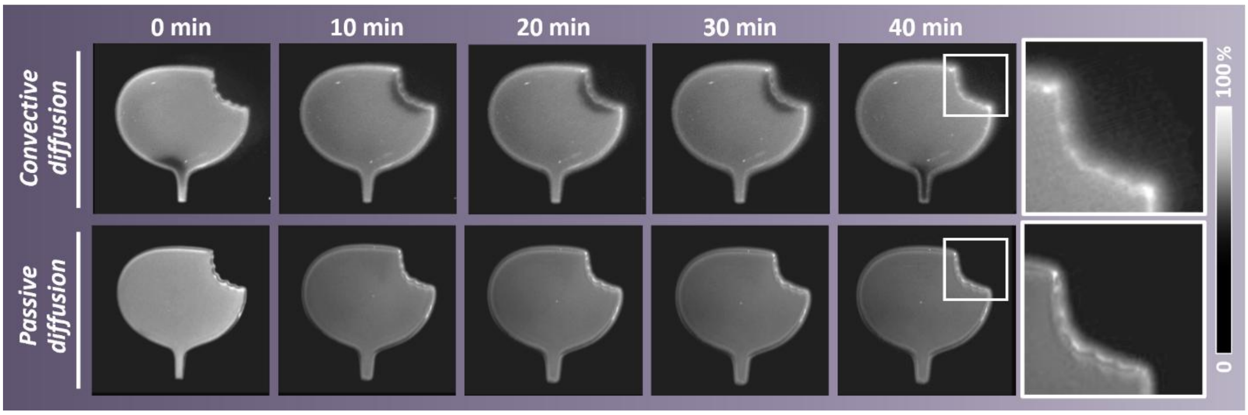

fig. S41. Assessment of DMCG-mediated drug penetration in a hydrogel-based artificial bladder-tumor model. NIR-II fluorescence image sequence of (top) DMCG-mediated convective diffusion versus (bottom) DMCG-free passive diffusion of ICG (serving as a model drug) penetrating across the cavity-shaped lesion site.

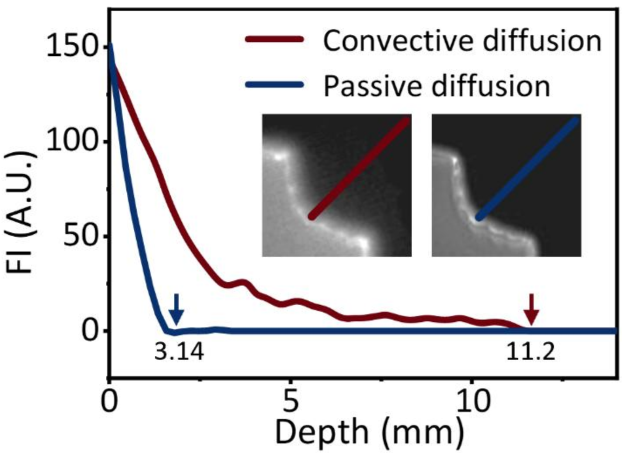

fig. S42 Fluorescence intensity profile of ICG penetrating across artificial bladder-tumor. Penetration depth of ICG by DMCG-mediated convective diffusion (11.2 mm as indicated by the red line) and DMCG-free passive diffusion (3.14 mm as indicated by the blue line).

Section S24. *In vivo* evaluation of convection-enhanced drug diffusion

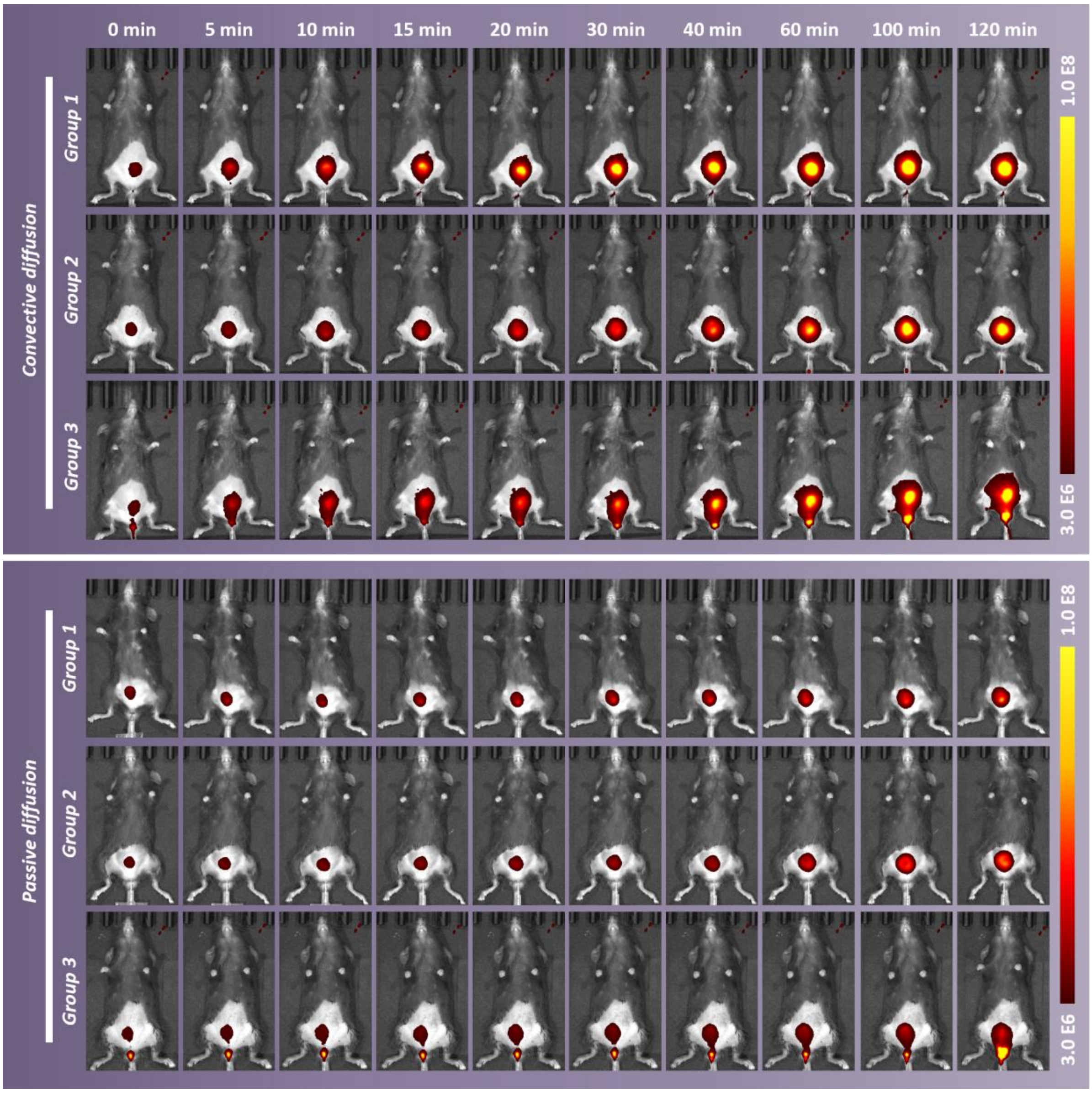

fig. S43. Supplementary fluorescence images of drug diffusion assessment in mouse bladder. Fluorescence image sequence of (top) DMCG-mediated convective diffusion versus (bottom) passive diffusion of high-concentration free-ICG solution (200 µg/mL) within the mouse bladder. n = 3 biologically independent mice per group.

The choice of ICG (FDA-approved molecule for clinical use) as a model drug in the fluorescence study was due to spectral interference: mouse tissue autofluorescence, which can broadly span 400–650 nm owing to contributions from lipofuscin and diet-derived pigments, overlaps with the emission of DOX (560–600 nm) and will compromises *in vivo* fluorescence readout. In contrast, ICG offers a higher signal-to-noise ratio in the near-infrared band (800–860 nm) and supports temporal fluorescence observation.

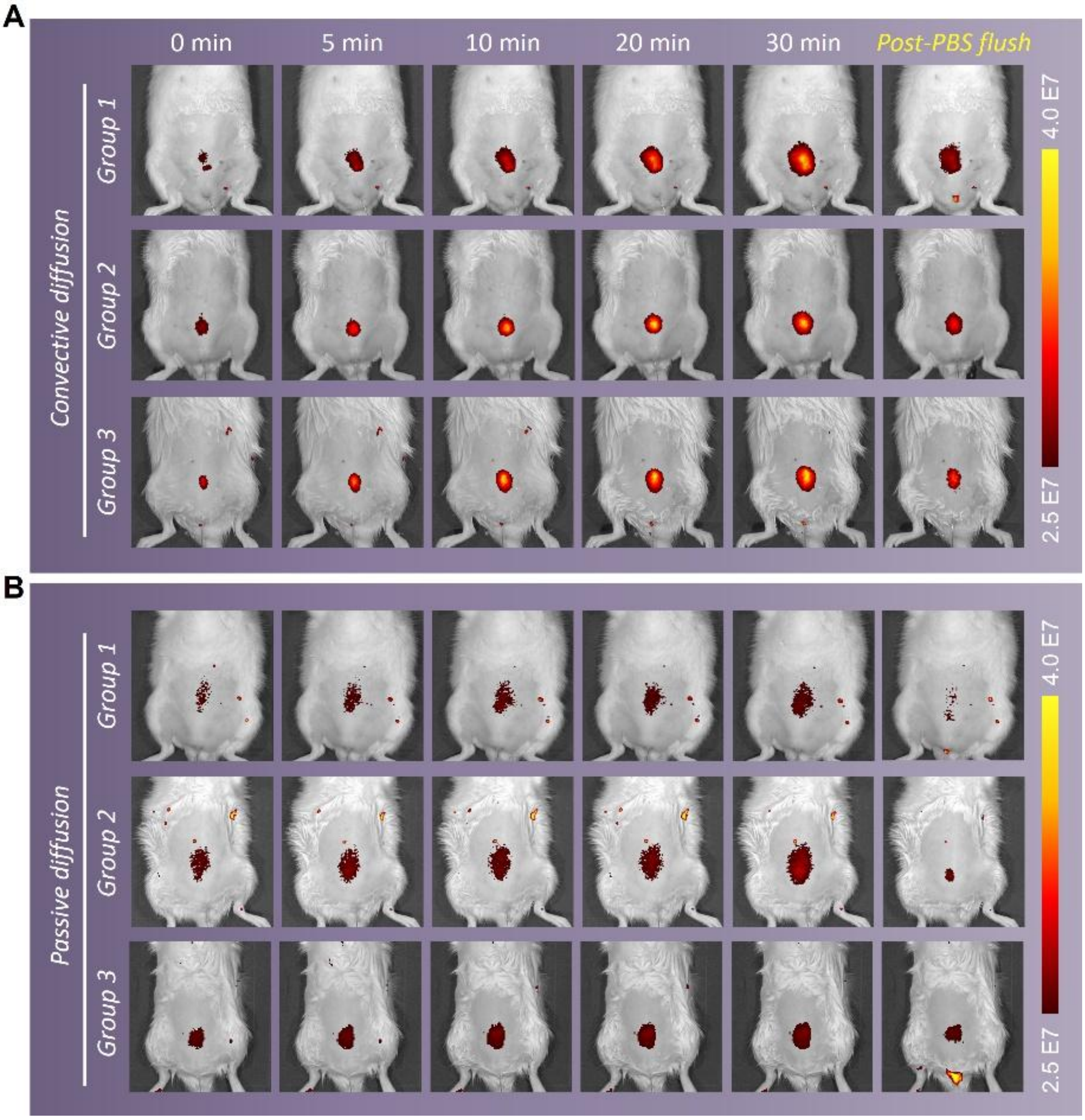

fig. S44. *In vivo* fluorescence imaging of ICG diffusion over 30 min and residual fluorescence after PBS rinsing in unfilled rat bladders. (A) Convective diffusion induced by ICG-loaded DMCG swarm. **(B)** Passive diffusion of a free ICG solution. n = 3 biologically independent rats per group.

Section S25. Evaluation of DOX permeation in bladder-tumor tissue sections

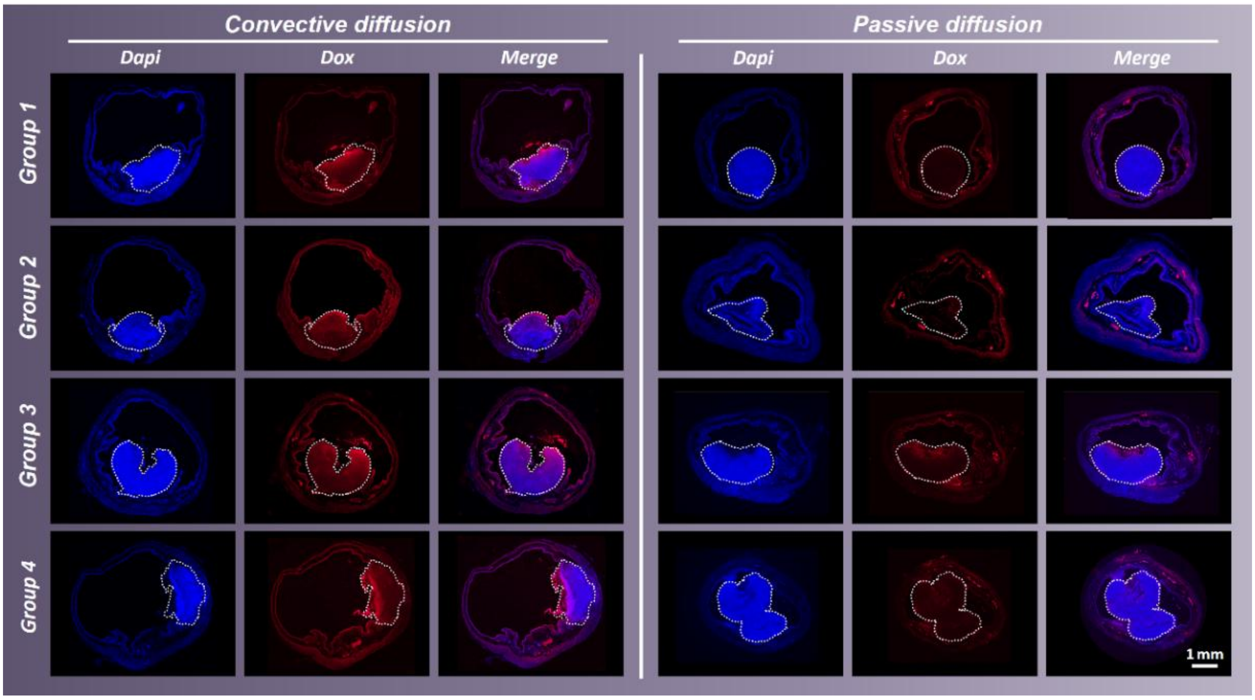

fig. S45. Fluorescence imaging of DOX permeation in tumor-bearing mouse bladder tissues. (left) DMCG-mediated permeation versus **(right)** passive permeation of clinical-dose DOX into the bladder tumor. n = 4 biologically independent mice per group.

This figure provides supplementary data for **Fig. 6b** with repeated experiments of DOX permeation in mouse bladder tumor. The tissue sections were stained with DAPI to locate the normal bladder and tumor tissues, with the tumor region outlined by dashed lines.

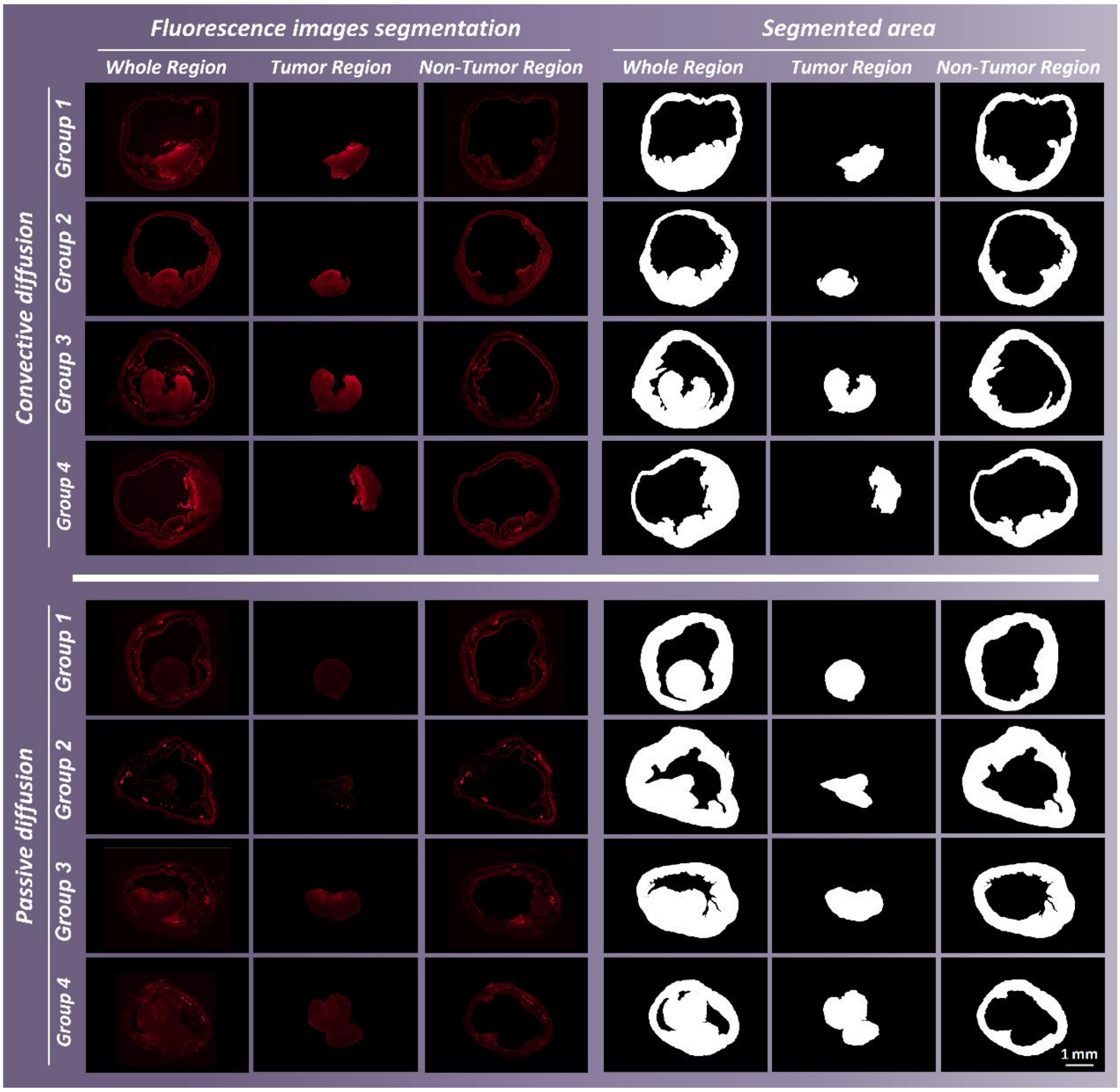

fig. S46. Segmentation of DOX fluorescence in tumor/non-tumor regions. (top) DMCG-mediated permeation versus **(bottom)** passive permeation of clinical-dose DOX within the bladder. n = 4 biologically independent mice per group.

This figure presents additional data for segmentation of DOX fluorescence in the tissue slices. The segmentation was performed based on the DAPI fluorescence images (**fig. S45**), which were used to locate the bladder tissue. The DOX fluorescence images were segmented into the whole region (WR), tumor region (TR), and non-tumor region (N-TR). The resulting segmented fluorescence images were then imported into ImageJ software for total fluorescence intensity (TFI) analysis, where the three regions were converted into binary images with the white pixels representing the segmented area. These binary images were also used for calculation of the area A_{region} of each region, thereby allowing for the calculation of mean fluorescence intensity (MFI) in each of the three regions through the formula: $\mathrm{MFI}=\mathrm{TFI}/A_{region}$.

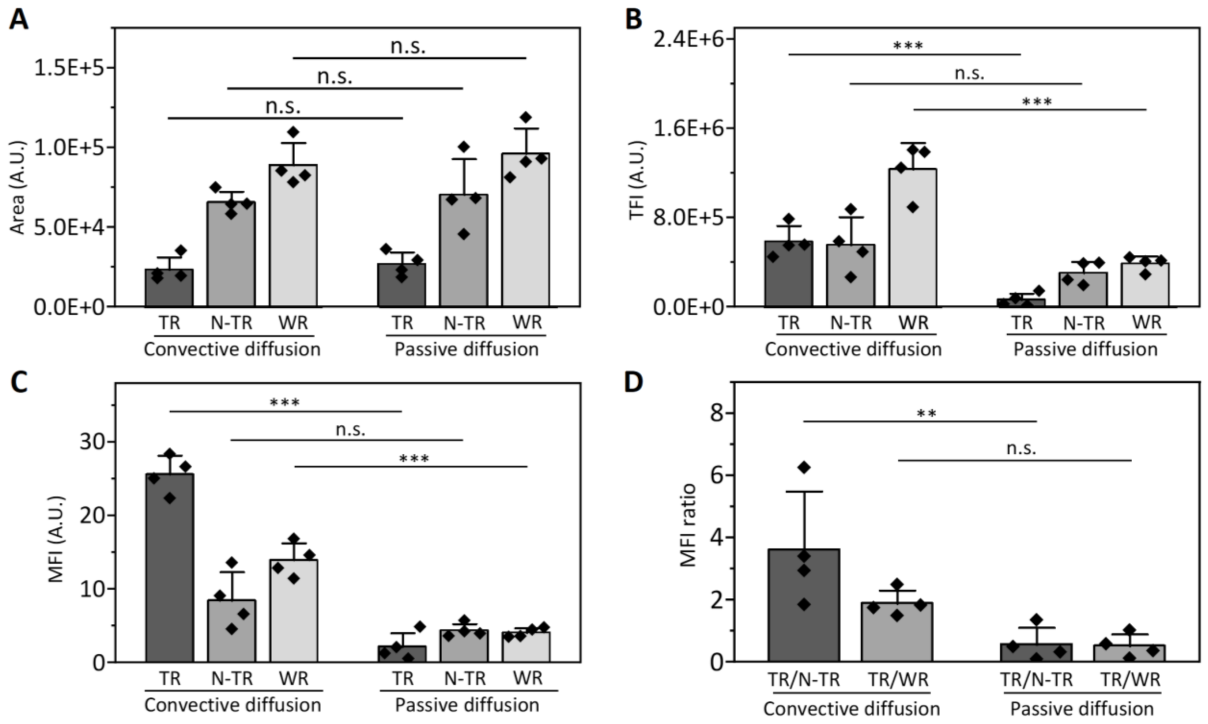

fig. S47. Quantification of DOX permeation in tumor-bearing mouse bladder tissues (based on DOX fluorescence data in fig. S45). Comparative analysis of **(A)** the calculated region-specific area, **(B)** total fluorescence intensity (TFI), and **(C)** mean fluorescence intensity (MFI) in the tumor region (TR), non-tumor region (N-TR), and whole region (WR) of bladder tissue sections. **(D)** compares the MFI ratio (indicator of tissue permeation specificity) between the DMCG group and free-DOX group. Data are mean ± s.d. (n = 4 biologically independent mice). Statistical significance was assessed by two-way repeated-measures ANOVA with Sidak's multiple-comparisons test. **A**: TR, P = 0.9805; N-TR, P = 0.9490; WR, P = 0.8553; **B**: TR, P = 0.0008; N-TR, P = 0.1257; WR, P < 0.0001; **C**: TR, P < 0.0001; N-TR, P = 0.0659; WR, P < 0.0001; **D**: TR vs N-TR, P = 0.0024; TR vs WR, P = 0.1577. Significance levels: *P<0.05, ** P<0.01, *** P<0.001, and n.s. for not significant.

This figure provides supplementary data for **Fig. 6c**. There is no significant difference in the calculated area of the TR, N-TR, and WR regions between the experimental group (DMCG-mediated convective diffusion) and the control group (free-DOX passive diffusion) as shown in **fig. S47A**, therefore confirming the validity of the average fluorescence intensity (MFI) data in **Fig. 6c**. Compared to the control group, the total fluorescence intensity (TFI) in the experimental group increased by 832.9% in the TR, 218.0% in the WR and 82.3% in the N-TR (**fig. S47B**). Further analysis of the MFI ratios (**fig. S47D**) between different regions reveals that the MFI ratio of TR/N-TR in the experimental group is approximately 3.6 versus 0.56 in the control group, indicating a six-fold increase in drug permeation selectivity.

Section S26. Histological examination and biochemical analysis of treated tumor

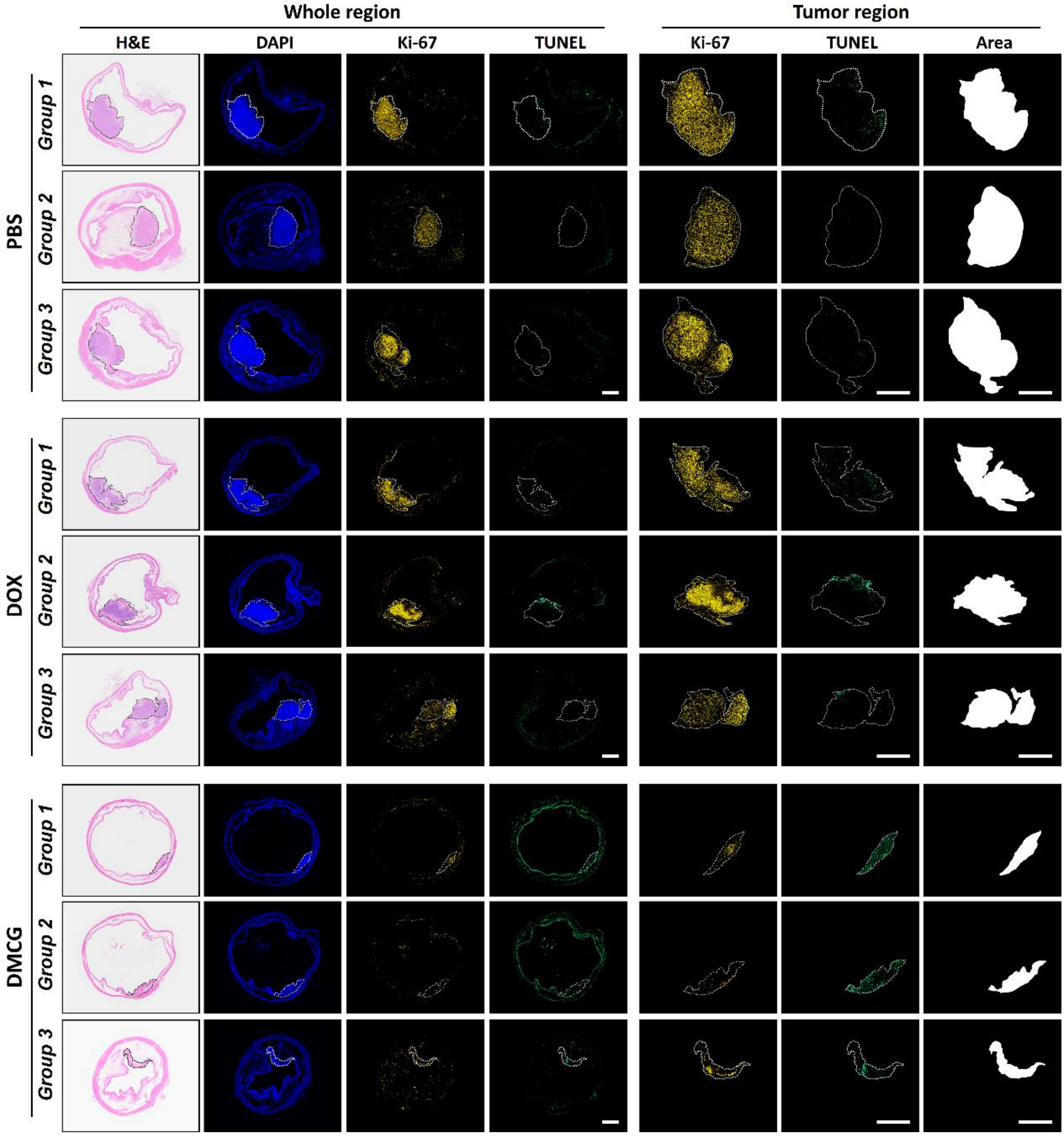

fig. S48. Histological analysis of mouse bladder tumor tissues after treatment. Tissue sections of **(left)** whole-bladder H&E staining and fluorescence labeling of DAPI (blue), Ki-67 (yellow), TUNEL (green); **(right)** segmented tumor-region fluorescence of Ki-67 immunostaining and TUNEL assay. The mice were treated with PBS, DOX, and DMCG, respectively. Scale bar, 500 μm. n =3 biologically independent mice per group.

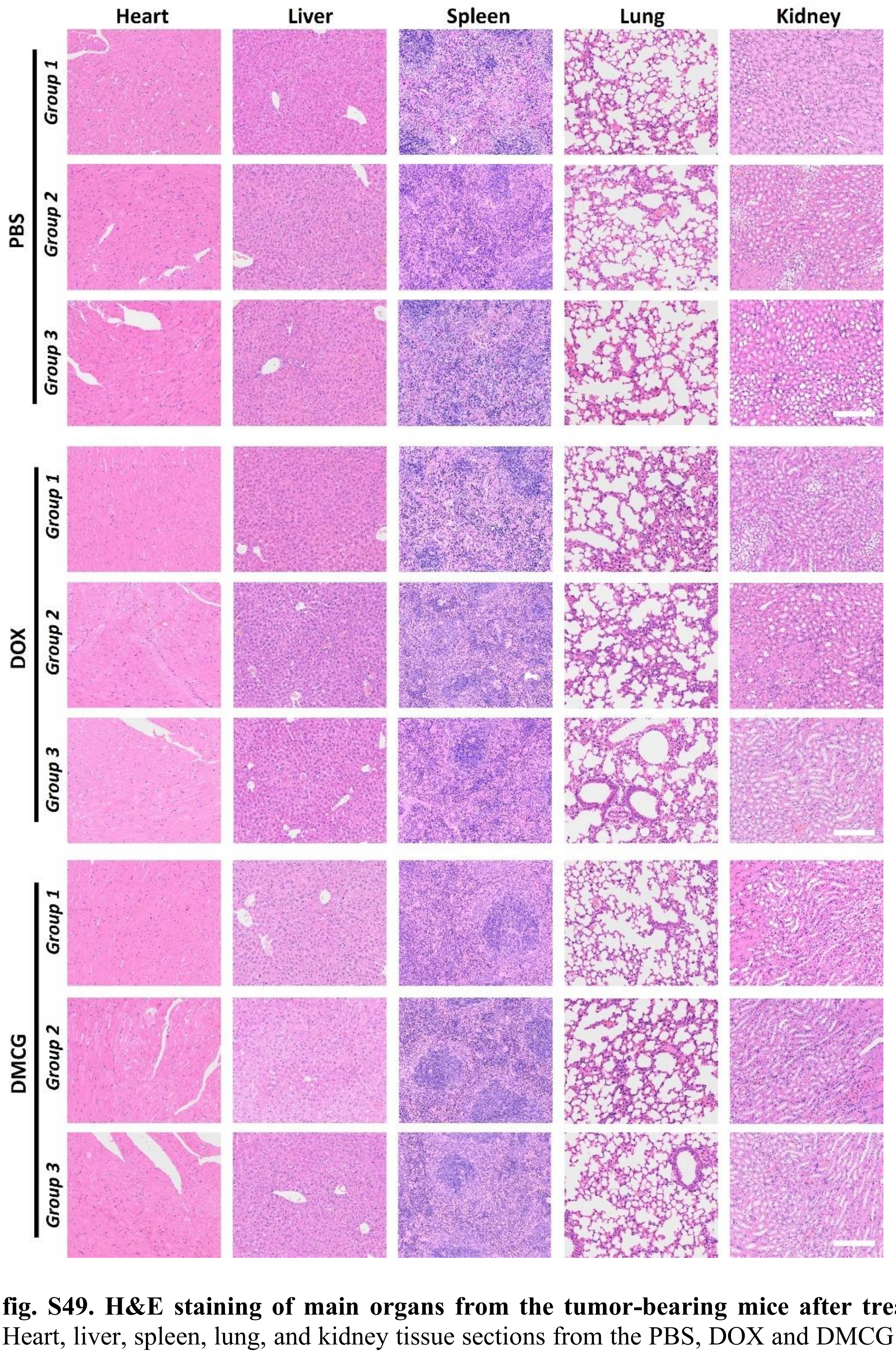

fig. S49. H&E staining of main organs from the tumor-bearing mice after treatment. Heart, liver, spleen, lung, and kidney tissue sections from the PBS, DOX and DMCG groups. Scale bar, 100 μm. n = 3 biologically independent mice per group.

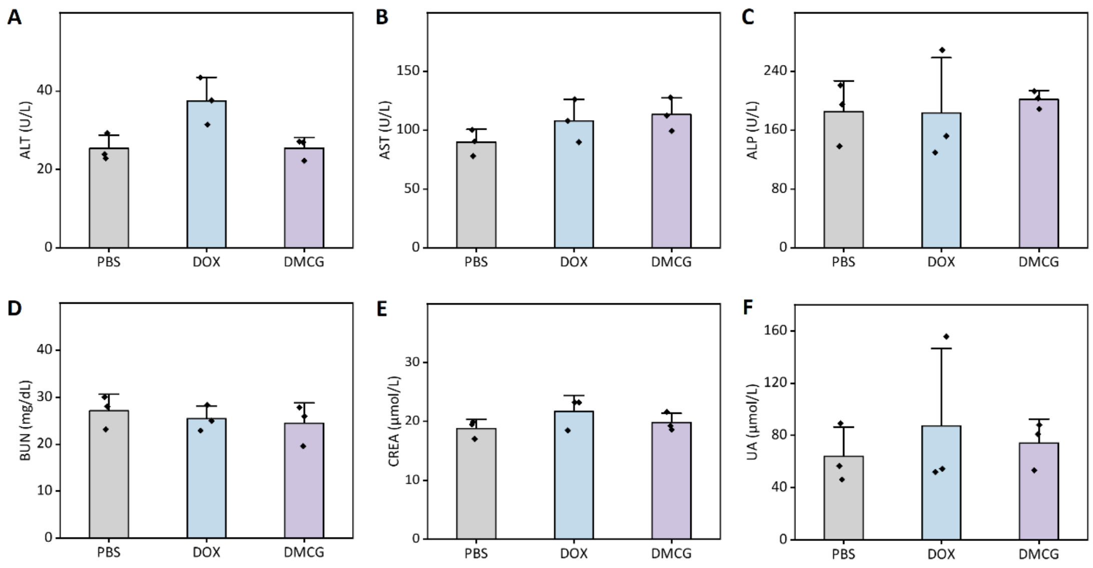

fig. S50. Serum biochemical analysis of hepatic and renal function after the treatment. **(A)** Alanine aminotransferase (ALT), **(B)** aspartate aminotransferase (AST), and **(C)** alkaline phosphatase (ALP) indicate the hepatic function. **(D)** Blood urea nitrogen (BUN), **(E)** creatinine (CREA), and (F) uric acid (UA) indicate the renal function. The mice were treated with PBS, DOX, and DMCG respectively. Data are mean ± s.d. from n =3 biologically independent mice per group.

Table S4. Comparative analysis of strategies for enhancing drug penetration into bladder tumor tissues. *Derived from the tissue section fluorescence data in (*58*). Acronyms: RITE, radio-frequency-induced thermochemotherapy; EMDA, electromotive drug administration; UPLC, ultra-performance liquid chromatography； DMSO, dimethyl sulfoxide.

Strategy	Drug/agent	Targeted delivery	Release control	Time (h)	Drug permeation enhancement		Evaluation method	Ref.
					Whole bladder	Tumor region		
RITE	Mitomycin C	No	No	1	173%	943%	UPLC	(*49*)
EMDA	Mitomycin C	No	No	0.5	289%	/	UPLC	(*50*)
DMSO	Paclitaxel	No	No	2	60%	/	UPLC	(*51*)
	Cisplatin	No	No	1	45%	/	Atomic absorption spectrometry	(*52*)
Nano-carrier	DOX	No	No	72	742%	120%	Urine evaluation	(*53*)
	10-hydroxycamptothecin (HCPT)	No	No	2	160%	/	Bladder tissue section fluorescence	(*54*)
	10-hydroxycamptothecin (HCPT)	No	No	2	120%	/	Bladder tissue section fluorescence	(*55*)
Nano-motor	CuO_2 nanoparticles	No	No	24	144%	/	Bladder tissue section fluorescence	(*56*)
	Fluorescein isothiocyanate isomer I (FITC)	No	No	12	45%	/	Bladder tissue section fluorescence	(*57*)
	siRNA	No	No	2	26%*	/	Bladder tissue section fluorescence	(*58*)
	Radioactive ^{18}F	No	No	3	/	150%	Radiopharmaceutical labeling	(*28*)
DMCG	DOX	Yes	Yes	0.5	242.3%	1083.6 %	Bladder tissue section fluorescence	this work

Supplementary Algorithms

Algorithm 1: Closed-loop control of ultrasound-guided navigation

---

Input : Continuous ultrasound stream V, control mode $\mathsf{Mode} \in \{\mathsf{COIL}, \mathsf{RMS}\}$
Output: Microrobot trajectory $Path_t$, magnetic control commands
Load UNet $\mathcal{M}_{\text{UNet}}$ and YOLO
Initialize control per Mode; microrobot, target positions R_0, T_0
while *stream V is active* **do**
    Acquire frame I_t ▷ ticks depend on actual frame acquisition
    if $I_t = \varnothing$
        HandleException(LOST_FRAME);**continue** ▷ skip frame if lost
    end

    $S_t \leftarrow \mathcal{M}_{\text{UNet}}(I_t)$ ▷ discrete class map
    $M_t \leftarrow \mathsf{NavMask}(S_t)$ ▷ morph. open on lumen; close on obstacles
    $I_{\text{color}} \leftarrow \mathsf{COLORIZE}(S_t)$ ▷ palette used in YOLO training
    $\{B_i, C_i\} \leftarrow \mathcal{M}_{\text{YOLO}}(I_{\text{color}})$ ▷ NMS inside
    $R_t \leftarrow \mathsf{Centroid}\big(\arg\max_{i:C_i=C_2} \text{conf}(B_i)\big)$ ▷ update microrobot position
    $T_t \leftarrow \mathsf{Centroid}\big(\arg\max_{j:C_j=C_1} \text{conf}(B_j)\big)$
    $Path \leftarrow \mathsf{BFS}(M_t, R_t, T_t)$ ▷ Algorithm 4
    if $Path \neq \varnothing$
        foreach *next waypoint* $N \in Path$ **do**
            if *Mode=COIL*
                $\mathsf{CoilExec}(R_t, N)$ ▷ Algorithm 5
            else
                $\mathsf{RMSExec}(R_t, N)$ ▷ Algorithm 6
            end
            $R_t \leftarrow N$ ▷ update pose estimate
            if *TerminationFlag*
                HandleException(MANUAL_STOP);**break** ▷ manual stop
            end
        end
    else
        HOLD(); **continue** ▷ no path; hold / replan next tick
    end
    ▷ Optional stream overlays (segmentation, path, pose)
End While

Algorithm 2: Segmentation post-processing (UNet-based)

Input : Ultrasound stream V
Output: Segmentation map S_t; navigation mask M_t

▷ UNet Classes: C_1: Target; C_2: Microrobot; C_3: Navigable space; C_4: Obstacle; C_5: Background
while *stream V is active* **do**
  Acquire frame I_t
  if $I_t = \varnothing$
    HandleException(LOST_FRAME); $S_t \leftarrow S_{t-1}$; **continue**
  end
  $U_t \leftarrow \mathcal{M}_{\text{UNet}}(I_t)$ ▷ Forward pass to get logits
  $S_t \leftarrow \arg\max(\text{Softmax}(U_t))$ ▷ Convert to discrete class map
  $T_{\text{nav}} \leftarrow \mathbf{1}\{S_t = C_3\}$; $T_{\text{obs}} \leftarrow \mathbf{0}\{S_t = C_4\}$ ▷ Binary masks for navigable space & obstacle
  $T_{\text{nav}} \leftarrow \text{MorphOpen}(T_{\text{nav}})$; $T_{\text{obs}} \leftarrow \text{MorphClose}(T_{\text{obs}})$ ▷ Noise removal & gap filling
  $M_t \leftarrow T_{\text{nav}} \wedge \neg T_{\text{obs}}$ ▷ Final navigation mask
  if *TerminationFlag*
    HandleException(MANUAL_STOP); **break** ▷ Emergency abort
  end
End While

Algorithm 3: Microrobot and target detection (YOLO-based)

Input : Segmentation map S_t from Algorithm 2
Output: Microrobot position R_t and target position T_t

▷ $\mathcal{M}_{\text{YOLO}}$ pretrained on COLORIZE(S_t) renders; Classes: C_1 Target, C_2 Microrobot
while *streaming* **do**
  if $S_t = \varnothing$
    HandleException(MISSING_SEGMENTATION); **continue**
  end
  $I_{\text{color}} \leftarrow$ COLORIZE(S_t)
  $\hat{D} \leftarrow \mathcal{M}_{\text{YOLO}}(I_{\text{color}})$
  $D \leftarrow \text{NMS}(\hat{D}, \text{conf} \geq \tau_c, \text{iou} \leq \tau_i)$ ▷ Non-maximum suppression, thresholds τ_c and τ_i set empirically
  if $\exists B_i \in D : C_i = C_2$
    $R_t \leftarrow \text{Centroid}\big(\arg\max_{i:C_i=C_2} \text{conf}(B_i)\big)$
  else
    $R_t \leftarrow$ NULL
  end
  if $\exists B_j \in D : C_j = C_1$
    $T_t \leftarrow \text{Centroid}\big(\arg\max_{j:C_j=C_1} \Phi(B_j)\big)$ ▷ Φ: tie-break (largest area or nearest to previous target)
  else
    $T_t \leftarrow$ NULL
  end
  if *TerminationFlag*
    HandleException(MANUAL_STOP); **break**
  end
End While

Algorithm 4: BFS-based path planning

Input : Navigation mask M_t from Algorithm 2, start $s = R_t$, goal $t = T_t$

Output: Shortest path $Path_t$ from s to t (if exists)

while *navigation session active* **do**
    if $M_t = \varnothing$ ***or*** $s =$ *NULL* ***or*** $t =$ *NULL*
        HandleException(MISSING_INPUT); $Path_t \leftarrow \varnothing$;**continue**
    end
    $s \leftarrow$ ProjectToFree(s, M_t); $t \leftarrow$ ProjectToFree(t, M_t)
    ▷ Use 4-neighbour connectivity to avoid diagonal corner-cutting. If 8-neighbour is required, forbid diagonals when both adjacent orthogonals are obstacles.
    $Q \leftarrow \langle\rangle$;
    $\pi \leftarrow \{\}$;
    $\text{dist}[v] \leftarrow \infty \ \forall v$;
    $\text{dist}[s] \leftarrow 0$;
    Enqueue(Q, s)
    while $Q \neq \varnothing$ **do**
        $u \leftarrow$Dequeue(Q);
        if $u = t$
            break
        end
        foreach $v \in Adj_4(u) \cap M_t$ **do**
            if $\pi[v] =$ ***null***
                $\pi[v] \leftarrow u$;
                $\text{dist}[v] \leftarrow \text{dist}[u] + 1$;
                Enqueue(Q, v)
            end
        end
    End While
    if $\pi[t] =$ ***null***
        $Path_t \leftarrow \varnothing$
    else
        $Path_t \leftarrow []$;
        $u \leftarrow t$;
        while $u \neq$ ***null*** **do**
            $Path_t$.Append(u);
            $u \leftarrow \pi[u]$
        End While
        $Path_t \leftarrow$ Reverse$(Path_t)$
    end
    return $Path_t$ // $\mathcal{O}(HW)$ on $H \times W$ grid
End While

Algorithm 5: Magnetic control execution I (Coil-based; image-frame control)

Input : BFS $Path = \{N_0, \ldots, N_k\}$ (pixels), current microrobot R_t

Output: Coil commands (yaw α) drive microrobot along $Path$

Initialize: Set serial port (e.g., COM1) to 115200 baud; set magnetic drive frequency f, yaw bias α_{bias}, and pitch angle β in accord with experimental calibration.

while *Path not empty* **do**

    $N_{\text{next}} \leftarrow$ next node in $Path$

    $\Delta x \leftarrow N_{\text{next}}[0] - R_t[0]$; $\Delta y \leftarrow N_{\text{next}}[1] - R_t[1]$

    Compute desired angles:

        $\alpha \leftarrow \text{atan2}(\Delta y, \Delta x)$ ▷ Yaw angle in image frame

    Send coil command:

        Transmit(α, β) ▷ Continuous stream to magnetic controller

    Update current position: $R_t \leftarrow N_{\text{next}}$

    Remove node from path: PopFront$(Path)$

    if *TerminationFlag*

        HandleException(`MANUAL_STOP`); **break**

    end

End While

Algorithm 6: Magnetic control execution II (RMS; world-frame pure pursuit)

Input : BFS path $Path = \{N_0, \ldots, N_k\}$ (pixels), current microrobot R_t (world), calibration matrix H

Output: RMS commands driving microrobot along $Path$

Initialize: Connect to RMS controller (e.g., `CCClient`); Set override speed, initial target T_0 and incremental step size r in accord with experimental calibration.

while *Path not empty* **do**

  $N_{\text{next}} \leftarrow$ next node in $Path$; $N_{\text{world}} \leftarrow \mathsf{Pixel2World}(N_{\text{next}}, H)$

  $\Delta x \leftarrow N_{\text{world}}[0] - R_t[0]$; $\Delta y \leftarrow N_{\text{world}}[1] - R_t[1]$; $\theta \leftarrow \text{atan2}(\Delta y, \Delta x)$

  $T_{\text{current}} \leftarrow T_{\text{current}} + r \cdot (\cos\theta, \sin\theta)$

  `cps.moveL`(T_{current}); `cps.waitMoveDone`()

  $R_t \leftarrow N_{\text{world}}$; PopFront($Path$)

  if *TerminationFlag (debounced 2 frames)*

    HandleException(`MANUAL_STOP`);**break**

  end

End While

Supplementary Videos

Video S1. Workflow for applying machine-intelligent multimodal DMCG microrobots toward efficient targeted intracavitary chemotherapy.

Video S2. Magnetic control and motion regimes of single DMCG.

Video S3. Directed motion of single DMCG along predefined paths.

Video S4. Autonomous navigation and real-time path-planning in complex mazes.

Video S5. Multi-body dynamics and controlled locomotion of multiple DMCGs.

Video S6. Multi-modal control and reconfigurable pattern of DMCG swarms.

Video S7. Simulation of the flow field perturbed by single-body and multi-body rotating DMCGs.

Video S8. Simulation of the drug-release concentration field and convective/diffusive fluxes by single-body and multi-body rotating DMCGs.

Video S9. Simulation of the drug-penetration concentration field and convective/diffusive fluxes by single-body and multi-body rotating DMCGs.

Video S10. *In vitro* assessment of drug release/penetration efficiency in an artificial bladder-tumor model.

Video S11. *In vivo* validation of intracavitary chemotherapy with DMCG microrobots in a mouse bladder-tumor model.
