# Supplementary material for: Machine-intelligent multimodal algebot for intracavitary chemotherapy
Source: Nat Nanotechnol. 2026 Jun 22;21(7):996–1007. doi: 10.1038/s41565-026-02195-0 (PMC13379317; doi:10.1038/s41565-026-02195-0)
Supplement: Supplementary file 2 — Reporting Summary [file 41565_2026_2195_MOESM2_ESM.pdf]

Corresponding author(s): Qi Zhou, Xiaohui Yan

Last updated by author(s): May 4, 2026

Reporting Summary

Nature Portfolio wishes to improve the reproducibility of the work that we publish. This form provides structure for consistency and transparency in reporting. For further information on Nature Portfolio policies, see our Editorial Policies and the Editorial Policy Checklist.

Statistics

For all statistical analyses, confirm that the following items are present in the figure legend, table legend, main text, or Methods section.

n/a	Confirmed	
☐	☒	The exact sample size (*n*) for each experimental group/condition, given as a discrete number and unit of measurement
☐	☒	A statement on whether measurements were taken from distinct samples or whether the same sample was measured repeatedly
☐	☒	The statistical test(s) used AND whether they are one- or two-sided<br>*Only common tests should be described solely by name; describe more complex techniques in the Methods section.*
☐	☒	A description of all covariates tested
☐	☒	A description of any assumptions or corrections, such as tests of normality and adjustment for multiple comparisons
☐	☒	A full description of the statistical parameters including central tendency (e.g. means) or other basic estimates (e.g. regression coefficient) AND variation (e.g. standard deviation) or associated estimates of uncertainty (e.g. confidence intervals)
☐	☒	For null hypothesis testing, the test statistic (e.g. *F*, *t*, *r*) with confidence intervals, effect sizes, degrees of freedom and *P* value noted<br>*Give P values as exact values whenever suitable.*
☒	☐	For Bayesian analysis, information on the choice of priors and Markov chain Monte Carlo settings
☒	☐	For hierarchical and complex designs, identification of the appropriate level for tests and full reporting of outcomes
☒	☐	Estimates of effect sizes (e.g. Cohen's *d*, Pearson's *r*), indicating how they were calculated

Our web collection on statistics for biologists contains articles on many of the points above.

Software and code

Policy information about availability of computer code

Data collection	Data were collected using manufacturer-provided acquisition software integrated with imaging systems, including Zeiss Observer 7 fluorescence microscopy, LSM 980 confocal microscopy (Carl Zeiss AG), Olympus VS200 slide scanning system, Vevo 2100 ultrasound imaging system (VisualSonics), and IVIS Lumina III-2 imaging system (PerkinElmer). Motion videos were recorded using a MER-503-36U3C camera (Daheng Imaging). Numerical simulation data were generated using COMSOL Multiphysics (version 6.1).
Data analysis	Image analysis and particle tracking were performed using Fiji (ImageJ) with the TrackMate plugin. Flow field quantification was performed using standard particle image velocimetry (PIV) analysis. The post-processing of numerical simulation was carried out in COMSOL Multiphysics (version 6.1). Data processing and visualization were performed using Microsoft Excel (Microsoft 365) and OriginPro (version 2021). Statistical analyses were performed using GraphPad Prism (version 9.4).

For manuscripts utilizing custom algorithms or software that are central to the research but not yet described in published literature, software must be made available to editors and reviewers. We strongly encourage code deposition in a community repository (e.g. GitHub). See the Nature Portfolio guidelines for submitting code & software for further information.

Data

Policy information about availability of data

All manuscripts must include a data availability statement. This statement should provide the following information, where applicable:

- Accession codes, unique identifiers, or web links for publicly available datasets
- A description of any restrictions on data availability
- For clinical datasets or third party data, please ensure that the statement adheres to our policy

All data supporting the findings of this study are available in the article and its Supplementary Information. Source data are available via Zenodo at https://doi.org/10.5281/zenodo.20009871. Apart from algorithms and pseudocode made available in the Supplementary Information, custom code developed for the deep-learning based intelligent navigation framework is available at GitHub: https://github.com/linlin-rylynn/Machine-Intelligent-Multimodal-Algebot-for-Intracavitary-Chemotherapy.git.

Research involving human participants, their data, or biological material

Policy information about studies with human participants or human data. See also policy information about sex, gender (identity/presentation), and sexual orientation and race, ethnicity and racism.

Reporting on sex and gender	This study did not involve human participants, and no human data or samples were collected. Only an established immortalized human cell line (SV-HUC-1) was used for in vitro experiments.
Reporting on race, ethnicity, or other socially relevant groupings	No human participants were involved.
Population characteristics	No human participants were involved.
Recruitment	No human participants were involved.
Ethics oversight	No human participants were involved.

Note that full information on the approval of the study protocol must also be provided in the manuscript.

Field-specific reporting

Please select the one below that is the best fit for your research. If you are not sure, read the appropriate sections before making your selection.

☒ Life sciences ☐ Behavioural & social sciences ☐ Ecological, evolutionary & environmental sciences

For a reference copy of the document with all sections, see nature.com/documents/nr-reporting-summary-flat.pdf

Life sciences study design

All studies must disclose on these points even when the disclosure is negative.

Sample size	No statistical methods were used to pre-determine sample sizes but our sample sizes are similar to those reported in previous publications (57, 58). The group size for each experiment is indicated in the corresponding figure legends or the Methods section.
Data exclusions	No animals or data points were excluded from the analyses unless explicitly noted.
Replication	All experiments were independently repeated at least three times with similar results unless otherwise stated in the figure legends. Biological and technical replicates were distinguished where appropriate.
Randomization	Animals were randomly assigned to experimental and control groups before treatment. Randomization was performed manually without dedicated randomization software. In vitro experiments did not involve allocation procedures requiring randomization.
Blinding	Data collection and analysis were not performed blind to the conditions of the experiments. Potential bias was minimized by using pre-specified, uniform acquisition and quantification settings, together with objective outcome metrics.

Reporting for specific materials, systems and methods

We require information from authors about some types of materials, experimental systems and methods used in many studies. Here, indicate whether each material, system or method listed is relevant to your study. If you are not sure if a list item applies to your research, read the appropriate section before selecting a response.

Materials & experimental systems

n/a	Involved in the study	
☐	☒	Antibodies
☐	☒	Eukaryotic cell lines
☒	☐	Palaeontology and archaeology
☐	☒	Animals and other organisms
☒	☐	Clinical data
☒	☐	Dual use research of concern
☒	☐	Plants

Methods

n/a	Involved in the study	
☒	☐	ChIP-seq
☒	☐	Flow cytometry
☒	☐	MRI-based neuroimaging

Antibodies

Antibodies used	Rabbit anti-Ki-67 primary antibody (Servicebio Co., Ltd., Wuhan, China; catalog no. GB111141), validated for mouse tissue, was used for immunofluorescence staining. The corresponding secondary antibody was CY5-conjugated goat anti-rabbit IgG (Servicebio Co., Ltd.; catalog no. GB27303).
Validation	The primary antibody against Ki-67 (Servicebio Co., Ltd., catalog no. GB111141) is validated by the manufacturer for immunofluorescence applications in mouse tissue, including tests for species reactivity and specificity. In this study, the staining pattern was consistent with the expected nuclear localization of proliferating cells.

Eukaryotic cell lines

Policy information about cell lines and Sex and Gender in Research

Cell line source(s)	SV-HUC-1 cells and MB49 cells were purchased from Cellverse Co., Ltd. (Shanghai, China). MB49-LUC cells were generated by stable lentiviral transduction of MB49 cells with a luciferase reporter construct at the State Key Laboratory of Vaccines for Infectious Diseases, Xiamen University. MTB-2 G3 murine bladder cancer cells were obtained from BeNa Culture Collection Co., Ltd. (Beijing, China). SV-HUC-1 cells are of human origin, whereas MB49, MB49-LUC and MTB-2 G3 cells are of murine origin.
Authentication	SV-HUC-1 cells were authenticated by short tandem repeat (STR) profiling by the supplier prior to purchase. MB49 and MTB-2 G3 cells were authenticated by species identification testing by the respective suppliers. MB49-LUC cells were derived from authenticated MB49 cells.
Mycoplasma contamination	All cell lines tested negative for mycoplasma contamination prior to use.
Commonly misidentified lines (See ICLAC register)	No commonly misidentified cell lines were used in this study.

Animals and other research organisms

Policy information about studies involving animals; ARRIVE guidelines recommended for reporting animal research, and Sex and Gender in Research

Laboratory animals	Female C57BL/6 mice (6 weeks old) and female Sprague–Dawley (SD) rats (12 weeks old) were used in this study.
Wild animals	This study did not involve wild animals.
Reporting on sex	Only female animals were used in this study, as bladder catheterization and intravesical administration are technically more feasible in female rodents. Sex was therefore controlled by design.
Field-collected samples	This study did not involve field-collected samples.
Ethics oversight	All animal procedures were approved by the Animal Care and Use Committee of Xiamen University (Approval Nos. XMULAC20190065 and XMULAC20250009) and conducted in accordance with institutional guidelines.

Note that full information on the approval of the study protocol must also be provided in the manuscript.

Plants

Seed stocks	This study did not involve plant materials.
Novel plant genotypes	This study did not involve plant materials.
Authentication	This study did not involve plant materials.
